# Supplementary material for: Dynamic mechanochemical feedback between curved membranes and BAR protein self-organization
Source: Nat Commun. 2021 Nov 12;12:6550. doi: 10.1038/s41467-021-26591-3 (PMC8589976; doi:10.1038/s41467-021-26591-3)
Supplement: Supplementary file 1 — Supplementary Information [file 41467_2021_26591_MOESM1_ESM.pdf]

Supplementary Information to

Dynamic mechanochemical feedback between curved membranes and BAR protein self-organization

Supplementary Note 1

1 Introduction

In previous work[1], we presented a general continuum framework to study the dynamics of curved protein-membrane interaction, highlighting the role of protein curvature and self-interaction on their curvature-sensing and generation capability. This theory accounted for protein diffusion and for membrane elasticity and hydrodynamics. We have also recently proposed a mean field theory to compute from statistical mechanics first principles the free energy and angular distribution of a collection of curved and elongated proteins, which interact anisotropically with the membrane, on surfaces of fixed curvature[2]. Here, to model the dynamical two-way interplay between shape and molecular organization of elongated and curved proteins, we combine these two theories, and include adsorption dynamics as discussed in[3]. Such continuum model can be numerically discretized to produce simulations reaching the micron scales of the observed protrusions and the seconds to minutes-long events of reshaping observed in our experiments, out of reach for most molecular simulations. However, compared to atomistic simulations, our model lacks molecular specificity, and as such it is difficult to map precise molecular features to mean-field parameters or functional forms. This is further discussed in Section 6.

With this extended theory, we can predict the coupled dynamics of protein area fraction ϕ, the shape of the membrane surface Γ parametrized by $\boldsymbol{x}(u, v, t)$, and nematic order. Orientational order is quantified by the traceless and symmetric tensor

$$\boldsymbol{Q} = S\left(\boldsymbol{\lambda}\otimes\boldsymbol{\lambda} - \frac{1}{2}\boldsymbol{I}\right), \tag{1}$$

capturing two important pieces of information: the order parameter S taking values between 0 and 1, where 0 corresponds to an isotropic organization of proteins and 1 to the maximum degree of order, and the net protein orientation given by the unit vector $\boldsymbol{\lambda}$. $\boldsymbol{I}$ is the identity tensor on the surface. In the following, we denote by $\boldsymbol{k}$ the second fundamental form or curvature of the surface, characterizing the curvature of the surface along any given direction.

2 Modeling the state prior to protein exposure

Prior to protein exposure, we model the formation of membrane protrusions following the conceptual and computational approach in[4] under the assumption of axisymmetry. We consider an inextensible membrane patch Γ of radius 2 μm interacting with a support through an interaction energy density per unit surface area $U(z)$, where z is the separation between the membrane and the substrate. The free energy of the membrane is

$$\mathcal{F}_{\text{mem}}[\boldsymbol{x}] = \int_\Gamma \frac{k_0}{2}H^2\, dS + \int_\Gamma U(z)\, dS, \tag{2}$$

where k_0 is the bending stiffness of the membrane and $H = (\text{tr}\,\boldsymbol{k})/2$ is the mean curvature and dS is the area element of Γ. This model does not account for the bilayer architecture, which despite the small radii of some membrane tubular protusions studied here, was shown to have a small impact on the mechanics since bilayer asymmetry can be relaxed through inter-monolayer sliding for a protrusion connected to a SLB acting as a reservoir[4].

To model the dynamics and as described elsewhere[5,6,7], we introduce a dissipation potential accounting for membrane viscosity $D_{\text{mem}}[\boldsymbol{v}]$, and obtain the governing equations of the system by minimizing the Rayleighian functional

$$\frac{d}{dt}\mathcal{F}_{\text{mem}}[\boldsymbol{v}] + D_{\text{mem}}[\boldsymbol{v}] + \int_\Gamma \sigma\, \text{tr}\, \boldsymbol{d}\, dS + p\int_\Gamma \boldsymbol{v}\cdot\boldsymbol{n}\, dS \tag{3}$$

with respect to the membrane velocity $\boldsymbol{v}$, where $\boldsymbol{d}$ is the rate-of-deformation tensor of the membrane, $\boldsymbol{n}$ is the outer normal to the surface, and the surface tension σ and pressure p are Lagrange multipliers that enforce the local inextensibility of the membrane and global incompressibility of the fluid enclosed between the membrane and the substrate. We numerically approximated these equations under the assumption of axisymmetry using a Lagrangian formulation and B-Spline basis functions.

Excess membrane resulting from lateral compression and excess enclosed volume resulting from osmotic imbalances lead to a variety of equilibrium membrane protrusions, which include long tubules, spherical buds and shallow spherical caps as mapped in[4]. All of these protrusions are observed in our experimental system. To study the effect of proteins on each of these types of structures, we prepared protrusions in equilibrium by laterally compressing the flat membrane and increasing the enclosed volume V with respect to V_0, the reference volume for a planar membrane at the equilibration separation z_0. Upon compression, the membrane delaminates to form a shallow spherical cap. By further increasing lateral compression and/or the enclosed volume, we obtained equilibrium structures consisting of spherical buds connected to the supported part of the bilayer by a narrow neck, or long tubular protrusions, see Figure. 2g in main text.

3 Modeling the protein-membrane interaction dynamics

Proteins on the membrane are represented by two fields, their area fraction ϕ and their nematic tensor $\boldsymbol{Q}$, which in our axisymmetric setting can be represented by the order parameter S and the angle θ with respect to the azimuthal direction.

Given the free-energy of the elongated and curved proteins on the membrane $\mathcal{F}_{\text{prot}}[\boldsymbol{x}, \phi, S, \theta]$, to be specified later and which depends on $\boldsymbol{x}$ through the curvature of the membrane, we can define the total free energy of the system as

$$\mathcal{F}[\boldsymbol{x}, \phi, S, \theta] = \mathcal{F}_{\text{mem}}[\boldsymbol{x}] + \mathcal{F}_{\text{prot}}[\boldsymbol{x}, \phi, S, \theta] + \int_\Gamma \frac{\Lambda_\phi}{2a_p}|\nabla\phi|^2\, dS + \int_\Gamma \frac{\Lambda_S}{2a_p}|\nabla S|^2\, dS + \int_\Gamma \frac{\Lambda_\theta}{2a_p}|\nabla\theta|^2\, dS, \quad (4)$$

where the last three terms regularize the phase boundaries between regions of different protein coverage, order and orientation.

We can write the Rayleighian functional of the membrane-protein system as

$$\frac{d}{dt}\mathcal{F}[\boldsymbol{v}, \boldsymbol{w}, \dot{S}, \dot{\theta}] + D_{\text{mem}}[\boldsymbol{v}] + D_{\text{prot}}[\boldsymbol{w}, \dot{S}, \dot{\theta}] + \int_\Gamma \sigma \operatorname{tr} \boldsymbol{d}\, dS + p \int_\Gamma \boldsymbol{v} \cdot \boldsymbol{n}\, dS, \quad (5)$$

where $\boldsymbol{w}$ is the net diffusive velocity of proteins relative to the lipids and $D_{\text{prot}}[\boldsymbol{w}, \dot{S}, \dot{\theta}]$ is the dissipation potential of proteins accounting for drag due to changes in position and nematic order of the proteins. σ is the Lagrange multiplier enforcing local membrane inextensibility and can be interpreted as the tension of the lipid membrane, whereas p is a pressure difference imposing a fixed volume of fluid enclosed between the membrane and the support. Minimization of the Rayleighian with respect to $\boldsymbol{v}$ leads to the equations of mechanical equilibrium governing shape dynamics and lipid flow. Minimization with respect to $\boldsymbol{w}$ leads to a generalized Fick's law relating $\boldsymbol{w}$ to the gradient of the chemical potential of the proteins, whereas minimization with respect to $\dot{S}$ and $\dot{\theta}$ leads to configurational balance equations. Here, we assume that S and θ relax much faster than $\boldsymbol{x}$ and ϕ. Combining Fick's law with the equation of balance of mass for proteins

$$\frac{\partial\phi}{\partial t} + \phi \operatorname{tr} \boldsymbol{d} + \nabla \cdot (\phi \boldsymbol{w}) = r, \quad (6)$$

where r is the sorption rate, we obtain a nonlinear diffusion-reaction equation for the protein density. All these equations are self-consistently coupled in this formalism. We refer to[1] for a full account of this formulation and of its computational axisymmetric implementation using a Galerkin finite element method based on B-Spline approximations. As boundary conditions, we imposed no protein flux at the edge of the membrane patch. The mechanical boundary conditions are discussed in Section 8.1 of this document.

We model sorption with a modified Langmuir model given by[3]

$$r = k_A c_{\text{bulk}}(\phi_{\max} - \phi)e^{-\beta\mu_{\text{mech}}} - k_D\phi, \tag{7}$$

where k_A is an adsorption rate constant, c_{bulk} the bulk concentration of proteins, μ_{mech} is the mechanical part (associated with their bending elasticity) of the chemical potential of proteins on the membrane, explicitly defined in Eq. (10), $1/\beta$ is the thermal energy, and k_D is a desorption rate constant. The exponential part of the adsorption term models an adsorption mechanism by which a curved molecule in solution must conform to the membrane curvature by a thermal fluctuation to become a membrane-bound protein, analogously to the case of binding of flexible adhesion molecules[8]. This has kinetic and thermodynamic consequences, as adsorption becomes faster and equilibrium coverage higher when the membrane curvature is close to the spontaneous curvature of the protein.

4 Free-energy of the elongated and curved proteins on a membrane

To understand the interaction between elongated curved proteins and a curved membrane, we developed in a companion paper[2] a new mean field density functional theory accounting for protein area coverage, orientational order and membrane curvature. This theory corrects in a curved and 2D setting Onsager's classical theory of isotropic-to-nematic transitions for non-spherical particles to provide quantitative prediction at high densities and moderate particle aspect ratio. It also accounts for the curvature energy of the proteins adsorbed on a curved surface. It is summarized next.

In[2],membrane curvature is considered as fixed. By coupling this mean field model with one of membrane shape dynamics, see Section 3, here we develop a self-consistent model for the two-way interplay between shape, molecular coverage and orientational order.

4.1 Mean field density functional theory

Following a mean field approximation and a passage to the continuum limit, the free-energy of the ensemble of elongated molecules is expressed in terms of the position-dependent number density of proteins ψ, related to the area fraction by $\phi = a_p\psi$ where a_p is the area of a protein, and the angular distribution f of proteins as

$$\mathcal{F}_{\text{prot}}[\boldsymbol{x}, \psi, f] = \frac{1}{\beta}\int_\Gamma \psi \ln\psi \, dS + \frac{1}{\beta}\int_\Gamma \psi \left\{ \int_{\mathbb{S}} f\left[\ln f - \ln g\right] d\gamma \right\} dS + \int_\Gamma \psi \int_{\mathbb{S}} f U^b \, d\gamma \, dS, \tag{8}$$

where $1/\beta = k_B T$ is the thermal energy and the set $\mathbb{S} = [-\pi, \pi)$ represents all possible orientations of molecules. The first term models the positional entropy of proteins, the second term accounts for orientational entropy and the excluded area though the function $g(\psi, \gamma) = 1 - \psi[c - dS P_2(\cos\gamma)]$ (with c and d parameters that depend on the geometry of the particles and $P_2(x) = x^2 - 1/2$), and the last term models the bending elasticity of proteins. The function $U^b(\boldsymbol{k}, \gamma)$ is the bending energy of an adsorbed protein oriented along the tangential vector $\boldsymbol{\ell}$ forming an angle γ with a fixed direction and is given by

$$U^b(\boldsymbol{k}, \gamma) = \frac{\kappa_p a_p}{2}(k_{\boldsymbol{\ell}} - \bar{C})^2, \tag{9}$$

where κ_p is its bending rigidity (with units of energy), $\bar{C}$ is its preferred curvature along the long axis, and $k_{\boldsymbol{\ell}} = \boldsymbol{\ell}\cdot\boldsymbol{k}\cdot\boldsymbol{\ell}$ is the normal curvature of the surface along the long direction of the protein. Equation (8) allows us to identify the mechanical part of the chemical potential of proteins as

$$\mu_{\text{mech}} = \int_{\mathbb{S}} f U^b \, d\gamma. \tag{10}$$

We do not consider here attractive interactions between proteins. We argue that if these were very strong, they would lead to aggregation even at low curvature and coverage.

Minimization of $\mathcal{F}_\mathrm{p}$ with respect to the angular distribution f yields an effective free energy depending only on the nematic tensor

$$\boldsymbol{Q} = \int_{\mathbb{S}} f(\gamma)\, \boldsymbol{\ell}(\gamma) \otimes \boldsymbol{\ell}(\gamma)\, d\gamma - \frac{1}{2}\boldsymbol{I}. \tag{11}$$

Since $\boldsymbol{Q}$ is traceless and symmetric, it can be expressed as in Eq. (1). In the axisymmetric setting considered here, $\boldsymbol{Q}$ can be parametrized by S and the angle θ between the nematic direction $\boldsymbol{\lambda}$ and the azimuthal direction. Denoting by k_1 and k_2 the principal curvatures of the surface at any point (which in the axisymmetric setting considered here are along symmetry directions,), we can express the free energy of the proteins as

$$\mathcal{F}_\mathrm{prot}[\boldsymbol{x}, \psi, S, \theta] = \int_\Gamma F_\mathrm{prot}(\psi, S, \theta, k_1, k_2)\, dS, \tag{12}$$

where the evaluation of the areal free-energy density $F_\mathrm{prot}(\psi, S, \theta, k_1, k_2)$ involves the solution of a nonlinear system of algebraic equations with two unknowns, see[2].Importantly, the only material parameters in this theory are the long and short axes of the ellipse modeling a protein, its preferred curvature $\bar{C}$ and its bending stiffness $\kappa_p a_p$, for which estimates are available.

This theory allows us to evaluate the free energy of proteins and study the isotropic-to-nematic transition on membranes adopting simple geometric motifs observed in our experiments, such as spheres and cylinders of various radii. On spheres, the free energy above is independent of θ whereas on cylinders it is minimized for $\theta = 0$ (proteins aligned with the direction of curvature) as long as the cylinder radius is larger than $1/\bar{C}$. Minimization with respect to θ allows us to compute the free-energy profile as a function of area coverage ϕ and order S alone, see Figure 2 and Supplementary Figure 3. These figures show that the free-energy landscape exhibits an order- and coverage-dependent forbidden region due to crowding effects as further discussed in[2]. It also shows that, for a planar and a spherical configuration, the model predicts a sharp and discontinuous isotropic-nematic phase transition with a range of intermediate protein coverages exhibiting coexistence of the two phases. The landscape on cylindrical surfaces is different in several ways. There, the isotropic-to-nematic transition is continuous and the isotropic phase is ordered even at low ϕ, particularly for thin tubes, due to the bias introduced by anisotropic curvature.

4.2 Explicit parametrization of the theory

This mean field theory connects the microscopic statistical physics with continuum physics and predicts the density- and curvature-dependent isotropic-to-nematic transition of proteins, but is cumbersome to evaluate and integrate in the computational framework described in Section 3 and in[1]. For this reason, we fit the free energy of proteins given by the mean field theory, $\mathcal{F}_\mathrm{prot}$, to an explicit functional form that we denote as $\hat{\mathcal{F}}_\mathrm{prot}$. Replacing $\mathcal{F}_\mathrm{prot}$ by $\hat{\mathcal{F}}_\mathrm{prot}$ is done for purely practical reasons. The explicit parametrization of the mean-field model is used in all simulations where molecular organization and membrane shape are solved for in a coupled way. The mean field model is only used Figure 2, b-f, but as shown in Supplementary Figure 3 both models agree very well.

To identify an ansatz for the functional form of $\hat{\mathcal{F}}_\mathrm{prot}$, we examine Eq. (8). Focusing first on the first two integrals in this equation, which do not depend explicitly on θ, and noting that $\langle P_2(\cos\gamma)\rangle = S/2$ where $\langle\ \rangle$ denotes the average with respect to f[2], we postulate the entropic part of the ansantz as

$$\hat{\mathcal{F}}_\mathrm{prot,entropy}[\boldsymbol{x}, \phi, S] = \frac{A_1}{\beta a_p}\int_\Gamma \left\{\phi\ln\phi - \phi\ln\left[1 - \phi\left(c - \frac{S^2}{2}d\right)\right]\right\} dS + \frac{A_2}{\beta a_p}\int_\Gamma \phi S^2 dS - \frac{A_3}{\beta a_p}\int_\Gamma \phi\ln(1-S) dS, \tag{13}$$

where A_1, A_2 and A_3 are non-dimensional fitting coefficients. The first integral accounts for positional entropy and excluded area, the second integral is a quadratic approximation to the order entropy, and the last integral allows us to fit the fast increase in the free-energy landscape for large ϕ and S, Supplementary Figure 3a1,b1.

Focusing now on the last term of Eq. (8), to propose an explicit functional form for the curvature energy of proteins, we note the following identity

$$\begin{aligned}\int_{\mathbb{S}} f(\gamma) U^b \, d\gamma &= \frac{\kappa_p a_p}{2} \int_{\mathbb{S}} f(\gamma) \left(\boldsymbol{k} : \boldsymbol{\ell} \otimes \boldsymbol{\ell} - \bar{C} \right)^2 d\gamma \\ &= \frac{\kappa_p a_p}{2} \int_{\mathbb{S}} f(\gamma) \left(\boldsymbol{k} \otimes \boldsymbol{k} :: \boldsymbol{\ell} \otimes \boldsymbol{\ell} \otimes \boldsymbol{\ell} \otimes \boldsymbol{\ell} - 2\bar{C} \boldsymbol{k} : \boldsymbol{\ell} \otimes \boldsymbol{\ell} + \bar{C}^2 \right) d\gamma \\ &= \frac{\kappa_p a_p}{2} \left[\boldsymbol{k} \otimes \boldsymbol{k} :: \langle \boldsymbol{\ell} \otimes \boldsymbol{\ell} \otimes \boldsymbol{\ell} \otimes \boldsymbol{\ell} \rangle - 2\bar{C} \boldsymbol{k} : \langle \boldsymbol{\ell} \otimes \boldsymbol{\ell} \rangle + \bar{C}^2 \right].\end{aligned} \tag{14}$$

where : denotes the double contraction of second-order tensors, :: the contraction of fourth-order tensors, and $\langle \; \rangle$ the average with respect to f. We note that $\boldsymbol{A} = \langle \boldsymbol{\ell} \otimes \boldsymbol{\ell} \rangle$ can be expressed exactly in terms of the nematic tensor as $\boldsymbol{A} = \boldsymbol{Q} + \frac{1}{2}\boldsymbol{I}$. To express $\langle \boldsymbol{\ell} \otimes \boldsymbol{\ell} \otimes \boldsymbol{\ell} \otimes \boldsymbol{\ell} \rangle$ in terms of $\boldsymbol{Q}$, we need to make an approximation, e.g. invoking the Doi closure[9]according to which $\boldsymbol{C} :: \langle \boldsymbol{\ell} \otimes \boldsymbol{\ell} \otimes \boldsymbol{\ell} \otimes \boldsymbol{\ell} \rangle \approx \boldsymbol{C} :: \langle \boldsymbol{\ell} \otimes \boldsymbol{\ell} \rangle \otimes \langle \boldsymbol{\ell} \otimes \boldsymbol{\ell} \rangle$ where $\boldsymbol{C}$ is a fourth-order tensor. Inserting this approximation into Eq. (14), we can rewrite the curvature part of the proteins free energy as

$$\begin{aligned}\int_{\Gamma} \psi \int_{\mathbb{S}} f U^b \, d\gamma \, dS &\approx \int_{\Gamma} \phi \frac{\kappa_p}{2} \left(\boldsymbol{k} : \boldsymbol{A} - \bar{C} \right)^2 dS = \int_{\Gamma} \phi \frac{\kappa_p}{2} \left(H + \boldsymbol{k} : \boldsymbol{Q} - \bar{C} \right)^2 dS \\ &= \int_{\Gamma} \phi \frac{\kappa_p}{2} \left[(1 - S) H + S k_{\lambda} - \bar{C} \right]^2 dS \\ &= \int_{\Gamma} \phi \frac{\kappa_p}{2} \left[H + S \frac{k_1 - k_2}{2} (2\cos^2\theta - 1) - \bar{C} \right]^2 dS,\end{aligned} \tag{15}$$

where $k_{\lambda} = \boldsymbol{\lambda} \cdot \boldsymbol{k} \cdot \boldsymbol{\lambda}$ is the normal curvature along the nematic direction. We checked that this approximation to the curvature part of the free energy was insufficient to closely fit the free energy of cylinders (particularly the minimum energy paths and isotropic-to-nematic transition in Supplementary Figure 3a) and for this reason we consider an expanded ansantz for the curvature free energy of proteins of the form

$$\begin{aligned}\hat{\mathcal{F}}_{\text{prot,curv}}[\boldsymbol{x}, \phi, S] = &\int_{\Gamma} \phi \frac{\kappa_p}{2} \left[H + S \frac{k_1 - k_2}{2} (2\cos^2\theta - 1) - \bar{C} \right]^2 dS \\ &+ \int_{\Gamma} \phi \frac{\kappa_p}{2} S \sum_{i=0}^{3} \frac{B_i}{\bar{C}^{i-2}} \left(\frac{k_1 - k_2}{2} (2\cos^2\theta - 1) - \bar{C} \right)^i dS,\end{aligned} \tag{16}$$

where B_i are nondimensional fitting parameters. Whereas the functional form of the first line of this ansatz for the curvature free-energy of proteins is justified by the Doi closure, the second line is purely phenomenological and its merit lies only in its ability to accurately fit the mean field theory. We note that the new term in the second line only adds a constant unless curvature is anisotropic. Combining Eqs. (13,16), we obtain an explicit form of the protein free-energy functional $\hat{\mathcal{F}}_{\text{prot}} = \hat{\mathcal{F}}_{\text{prot,entropy}} + \hat{\mathcal{F}}_{\text{prot,curv}}$ approximating the mean field functional $\mathcal{F}_{\text{prot}}$ and amenable to numerical calculations.

To fit the parameters A_i, we first focused on the purely entropic interaction of elliptical proteins on a flat membrane, which we evaluated with the mean field model in Eq. 8. We then fitted the functional proposed in Eq. 13 to the mean field landscape using a nonlinear least-squares method. In a second step, we included the bending energy in the mean field model, computed the free-energy landscape for flat, spherical and cylindrical configurations with different curvatures, see Supplementary Figure 3a, and used these free-energy landscapes to fit B_i using nonlinear leasts-squares.

See Supplementary Figure 3 for a comparison of the free-energy profiles obtained with both models. Although there are noticeable differences, these are small and the approximate functional $\hat{\mathcal{F}}_{\text{prot}}$ captures quantitatively the most salient features of the mean field model including the curvature-dependent isotropic-to-nematic transition.

We note that if the radius of curvature of a cylindrical membrane is larger than that of the protein $1/\bar{C}$, then the free energy is minimized for $\theta = 0$[2].Thus, although we fitted the mean fit model to include

the smaller radii, in all our simulations radii of curvature were larger than $1/\bar{C}$, and thus the free-energy functional can be simplified by setting $2\cos^2\theta - 1 = 1$.

5 Model parameters

The model parameters are reported in Supplementary Table 1. Following[4], we assume a Morse potential for $U(z)$ with a membrane-support equilibrium distance of z_0 and adhesion energy $-U(z_0)$. Once the membrane has delaminated, this potential essentially controls how easy it is to separate the adhered membrane from its substrate, and hence the ability of the protrusion to exchange enclosed water volume with the adhered part of the membrane. The mechanical properties of the lipid membrane are the bending stiffness k_0 and the membrane 2D viscosity η. We consider N-BAR proteins to be elliptical with semi-axes lengths a and b as reported in the table, leading to the non-dimensional constants $c = 15.66$ and $d = 6$ appearing in the expression for the free-space as a function of density and order in Eq. (13). We assume that these proteins have an intrinsic curvature of $1/\bar{C}$, an area on the membrane of a_p, and a protein bending rigidity of $\phi_{\max} k_p$ at saturation coverage based on the rigidity of the membrane-protein compound of 40 k_BT[10,11,12]. D_p is the diffusion coefficient for proteins on the membrane.

The fitting procedure of the functional given by Eqs. (13,16) results in the non-dimensional coefficients $A_1 = 1.25$, $A_2 = 0.7$, $A_3 = 0.5$, $B_0 = 1.61$, $B_1 = -2.49$, $B_2 = 1.32$ and $B_3 = -0.43$. We consider an adsorption rate of k_A and a desorption rate to k_D in the order of that considered in previous works in a related system[13].

In the absence of measurements, we choose Λ_ϕ/a_p, Λ_S/a_p and Λ_θ/a_p large enough so that, when phase separation occurs, domain boundaries have a finite thickness and simulations are devoid of numerical oscillations indicating ill-conditioning of the equations, and small enough so that the dynamics of the problem are not affect by these parameters.

We finally discuss the selection of tube diameter. To estimate it, we noted that according to our model the number of pearls per unit tube length at the onset of reshaping is a function of tube diameter since pearl diameter is commensurate to tube diameter. As mentioned in the main text, it was not possible to measure tube diameter experimentally. However, we could estimate tube length and count the number of pearls at the onset of reshaping. By collecting experimental images of pearling events for tubes of different lengths and comparing them with simulations using different tube diameters and lengths, we found that a tube diameter of 600 nm could consistently recover the observed number of pearls.

Membrane-support equilibrium distance	z_0	4.4 nm	
Adhesion energy density	$U(z_0)$	0.075, 0.75, 1.5, 2.25 mJ/m^2	[4]
Spring constant in membrane area ensemble	k_{spring}	0, 0.32 mN/nm^2, $+\infty$	
Membrane viscosity	η	$5 \cdot 10^{-9}$N s/m	[14]
Protein semi-axis length	a, b	$a = 7.5$ nm, $b = 2.5$ nm	[15]
Intrinsic curvature of proteins	$1/\bar{C}$	15 nm^{-1}	Suppl. Fig. 2b exp. data
Area of proteins on the membrane	a_p	58 nm^2	[15]
Diffusion coefficient for proteins on the membrane	D_p	0.13 µm^2/s	[1]
Saturation protein area fraction	$\phi_{\max}$	≈ 0.75	[2]
Membrane bending rigidity	k_0	20 k_BT	[16]
Protein bending rigidity	k_p	27 k_BT	[17,18,19]
Adsorption rate	k_A	1/6 µM^{-1}s^{-1}	> [13]
Desorption rate	k_D	1/1800 s^{-1}	[13]
Phase boundary regularization for ϕ	Λ_ϕ/a_p	10 k_BT	
Phase boundary regularization for S	Λ_S/a_p	1 k_BT	
Phase boundary regularization for θ	Λ_θ/a_p	1 k_BT	

Supplementary table 1: Model parameters

6 Mapping between the model and the mechanisms coupling protein coverage to membrane curvature

The model described above describes the coupling between Amphiphysin and membrane curvature in a coarse-grained way, with an emphasis on the scaffolding by the BAR domain. However, there are other coupling mechanisms resulting from domains partially inserting on the bilayer or from bulky disordered domains. Although a continuum model such as the one used here lacks molecular specificity, it is possible to discuss the mapping between molecular mechanisms and effective parameters and functional forms in the continuum description.

At the level of our coarse-grained description of protein-membrane interactions, the wedge insertion mechanism cannot be distinguished from the scaffolding effect because it should impinge an orientation-dependent spontaneous curvature, and hence, it is included in our effective material parameters.

Insertions can also couple to curvature because they can modify the lipid packing asymmetrically in one monolayer and significantly influence protein-membrane interactions, as shown in small vesicle assays in[20]. We argue that this effect is not likely to play a major role in the present system where the upper monolayer of protrusions is connected to a lipid reservoir (the flat part of the monolayer) and hence packing differentials of lipids can be relaxed by inter-monolayer sliding[4,21].

Finally, disordered bulky domains can mediate in couplings between Amphiphysin and membrane curvature. At low coverage, in the curvature sensing regime, the coupling is due to the dependence of chain entropy on membrane curvature[22]. In this regime, protein organization is isotropic, and as shown in[23] the effect of the disordered domain can be mapped to a spontaneous curvature. Thus, we can consider that it is effectively included in our model.

At high coverage, bulky disordered domains couple to curvature through a crowding effect of these domains at a distance from the membrane surface[24]. This effect should be largely isotropic in a regime, high-coverage, where BAR domains tend to align and have an anisotropic contribution to the bending free-energy. Thus, unlike other mechanisms discussed above, the effect of crowding of bulky disordered domains cannot be assumed to be included in our model. To examine the impact of this contribution as compared to that of the BAR domain, we turned to the model in[1] for the coupling between membrane curvature and the coverage of such bulky disordered domains. For simplicity, we assume that proteins have a BAR domain and a disordered domain modeled as a spherical blob of radius d. The disordered domains effectively interact at a plane a distance d away from the lipid membrane as shown in Supplementary Figure 9. As shown in[1] the effective bending energy of a system composed of an ensemble of the disordered domains and a membrane is of the form

$$\int_\Gamma \frac{1}{2}\left[k_0 H^2 - 2k_0 H C(\phi)\right] dS. \tag{17}$$

The spontaneous curvature imposed by disordered domains takes the form

$$C(\phi) = \frac{d}{a_p k_0}\left\{\frac{\chi}{2}\phi^2 - k_B T \phi_m \left[\log(\phi_m - \phi) + \log\frac{a_d}{a_p}\right]\right\},$$

where a_d is the projected area of the disordered domain, k_0 the bending stiffness of the membrane, χ the specific interaction between disordered domains and ϕ_m the saturation area fraction of proteins. We note that in this expression, ϕ is the area fraction of the membrane-bound BAR domain.

To describe the bending free-energy of BAR proteins endowed with bulky disordered domains, we combine the above functional with that presented in Section 4 to obtain

$$\mathcal{F}_{\mathrm{d}} = \int_\Gamma \frac{1}{2}\left[k_0 H^2 - 2k_0 H C(\phi)\right] dS + \mathcal{F}_{\mathrm{prot,curv}}. \tag{18}$$

To quantify the role of disordered domains, we compare this bending free-energy with that ignoring the presence of disordered domains

$$\mathcal{F}_{\mathrm{no-d}} = \int_\Gamma \frac{1}{2} k_0 H^2 \, dS + \mathcal{F}_{\mathrm{prot,curv}}. \tag{19}$$

We assume that disordered domains only interact entropically ($\chi = 0$), that their characteristic distance to the membrane is $d \approx 6$ nm, that the area per disordered domain is $a_d = 75$ nm^2 [22], that the maximum area fraction is $\phi_m \approx 0.75$ and that the membrane bending rigidity is $k_0 = 20\ k_B T$. The comparison of $\mathcal{F}_{\rm d}$ and $\mathcal{F}_{\rm no-d}$ on fixed cylindrical and spherical shapes of different radii and for different protein area fractions ϕ (and the corresponding S minimizing $\mathcal{F}_{\rm prot,curv}$), Supplementary Figure 9, shows that the contribution of bulky disordered domains does not significantly affect the bending energy. The effect is noticeable at high coverage and for small curvatures. We further examine the effect of ϕ_m by considering a smaller value, which should increase the role of bulky domains, but their contribution is still very small and is neglected for the purpose of our study.

7 Simulation protocol for protein-membrane interaction and discussion of dynamics

To computationally examine the effect of BAR proteins on pre-existing membrane protrusions, we start from tubular or spherical protrusions in mechanical equilibrium as those in Figure 2g and following the computational protocol described in Section 2 of this supplement. Then, we prescribe the protein concentration in the medium, $c_{\rm bulk}$, and computationally track the dynamics of the system as described in Section 3 of this supplement with the explicit free energy of proteins described in Section 4.2 of this supplement. As discussed in the main text, this leads to reshaping dynamics much faster than those observed experimentally.

7.1 Time-scales involved in reshaping, membrane diffusion and adsorption

To understand this discrepancy, we first examined the major time-scales involved in our model. We have already mentioned that we assume that orientational order relaxes very fast. At the small scales of our protrusions, smaller than the Saffman-Delbrück lengthscale, the main dissipative mechanism controlling shape dynamics is the membrane viscosity. With this in mind, we approximate the time required for membrane shape dynamics as $\tau_m \sim \bar{S}\eta/k_0$ where $\bar{S}$ is the typical surface area of a geometric feature[21], leading to the rough estimate $\tau_m \approx 0.01$ s. The timescale for protein diffusion is $\tau_p \sim \ell^2/D_{\rm p}$ where ℓ is either the radius of a spherical bud or the length of a tube where proteins diffuse. For the membrane protrusions studied here, we estimate τ_p to be a few seconds. The timescale for protein adsorption τ_A strongly depends on the bulk protein concentration $c_{\rm bulk}$ and on shape due to the coupling between adsorption kinetics and mechanics of the adsorbed proteins, see Eq. 7. Using this equation, we estimated τ_A to be of about 4 seconds for a protein concentration of $c_{\rm bulk} = 0.35$ μM and of about 14 seconds for $c_{\rm bulk} = 0.1$ μM, with faster dynamics for thinner tubes. Based on these estimations, membrane relaxation is the fastest phenomenon, whereas protein diffusion on the membrane and adsorption can in principle compete. Since the former is size-dependent whereas the second one is not, diffusion should be faster below a length-scale.

7.2 Mechanism of enrichment at necks

Given the complexity of the adsorption and reshaping dynamics, these estimates can only provide a rough idea of the actual dynamics as captured in the simulations. In particular, it is unclear whether the enrichment of protein on the elongating neck of membrane buds is due to preferential adsorption at the neck, see Eq. (7), or to diffusion towards the neck from neighboring membrane compartments by curvature sensing. According to our discussion of time-scales, diffusion may dominate for short necks. However, the difference in protein chemical potential between the neck and neighboring regions has two competing components, mechanical and entropic, which makes it difficult to estimate the sign and magnitude of the driving force for diffusion. To establish the main mechanism of protein enrichment, we quantify in our simulations the mass balance of adsorbed proteins in the neck region by tracking the number of proteins over time as

$$N_{\rm neck}(t) = \frac{1}{a_p}\int_{\Gamma_{\rm neck}(t)} \phi\, dS,$$

where $\Gamma_{\text{neck}}(t)$ is the neck region. We also track the total number of proteins delivered between two time-instants $t_1 < t_2$ by adsorption as

$$\Delta N_{\text{a}}^{t_1 \to t_2} = \frac{1}{a_p} \int_{t_1}^{t_2} \int_{\Gamma_{\text{neck}}(t)} r \, dS \, dt.$$

This allows us to establish the fraction of proteins delivered to the neck by adsorption as

$$f^{t_1 \to t_2} = \Delta N_{\text{a}}^{t_1 \to t_2} / (N_{\text{neck}}(t_2) - N_{\text{neck}}(t_1)).$$

This analysis shows that enrichment at necks is overwhelmingly due to adsorption rather than diffusion, with fractions over 0.98 even at the initial phases of neck elongation.

7.3 Slow time-scale in the experiments: bulk diffusion

With the ingredients discussed above, the reshaping dynamics in our simulations take a few seconds, whereas those in the experiments take over a minute, in some instances over 10 minutes. To explain this, we turn to the bulk diffusion of protein in the droplet following injection, see Methods.

Proteins are delivered to the membrane by a micro-injection of a solution of high protein concentration c_{inj} in the medium droplet, with the volume of the injection such that the average protein concentration in the droplet is the nominal concentration c_{bulk} reported in the main text, e.g. in Figure 3. Thus, proteins need to diffuse in this droplet from the point of injection to the SLB. This introduces an additional time-scale τ_D in the problem, besides those of adsorption τ_A, diffusion on the membrane τ_p, and membrane shape relaxation τ_m. To estimate τ_D, we consider a 1D diffusion equation with diffusion constant $D = 75$ μm^2/s on a domain of length 0.1 mm, representative of the distance between the injection point and the SLB, no flux boundary conditions, and an initial conditions with a high-concentration injected volume in a small region of the domain consistent with the nominal concentration in equilibrium. This model provides an estimate of the concentration in the bulk solution in the vicinity of the supported lipid bilayer as a function of time, $c_{\text{bulk,SLB}}(t)$.

This model shows that diffusion in the bulk is the slowest process, Supplementary Figure 5a, except for the largest protein concentrations where it is comparable to the other processes. For instance, upon an instantaneous increase of $c_{\text{bulk,SLB}}$ by 0.1 µM, the dynamics including adsorption, membrane diffusion and shape relaxation take about 10 seconds, whereas 100 seconds are needed to reach such increase in $c_{\text{bulk,SLB}}$ by diffusion in the bulk. Thus, the protrusions rapidly equilibrate chemically and mechanically to a slowly varying bulk concentration at the SLB. Taking advantage of this separation of time-scales, we performed simulations in which $c_{\text{bulk,SLB}}$ was changed in steps and the system was equilibrated for each value of $c_{\text{bulk,SLB}}$, resulting in a sequence of equilibrium states for varying concentration, Supplementary Figure 5d and 6d. We then used the diffusion equation in the bulk, Supplementary Figure 5a and 6a, to assign time these quasi-equilibria, Supplementary Figure 5c and 6c, finding a very good agreement with the experiments. For instance, it predicts that the same degree of bud elongation should be observed after about 10 seconds for $c_{\text{bulk,SLB}}$ by 0.5 µM, after 200 seconds for $c_{\text{bulk,SLB}}$ by 0.35 µM, and after 700 seconds for $c_{\text{bulk,SLB}}$ by 0.25 µM.

8 Sensitivity to the size of protrusions and to the mechanical ensemble

To establish the generality of our conclusions, we varied several key parameters in our model. We considered buds of different diameter (500 nm, 1 µm, 1.5 µm), tubes of ≈ 600 nm in diameter and of different lengths (2 µm, 3 µm, 5 µm). These variations replicated the diversity of shapes observed experimentally and were obtained by modifying the excess membrane and the excess enclosed volume. See Supplementary Figure 10.

Another important aspect of the model that we varied was the mechanical ensemble of the protrusion under study during the reshaping dynamics. In the actual system with many membrane protrusions interacting with proteins, the reshaping of one protrusion may result in membrane area and enclosed water volume

exchange with the rest of the system, in particular with the adhered part of the membrane surrounding it. Since in our computational model we study one protrusion in isolation, we need to specify a mechanical ensemble controlling lipid and enclosed volume exchange between a protrusion and the adhered membrane. Since we do not have direct experimental access to the membrane and enclosed volume exchange between a reshaping protrusion and its surroundings, we examined how sensitive were the results to the choice of mechanical ensemble.

8.1 Area/tension ensemble

Regarding membrane area/tension, we interpolated between two extreme cases, (I) fixed tension, which permits membrane area exchange with the surroundings, and (II) fixed projected area of the membrane, which precludes such exchange. To achieve this, we considered a boundary condition according to which membrane tension at the edge of the simulation domain is given by $\sigma_0 + k_{\text{spring}}\delta$ where σ_0 is the equilibrium tension for the protrusion prior to protein addition, δ is the displacement of the edge of our domain and k_{spring} is a parameter such that $k_{\text{spring}} = 0$ corresponds to the fixed tension ensemble (I) and $k_{\text{spring}} = +\infty$ to (II). We varied this parameter as indicated in Supplementary Table 1.

8.2 Volume/pressure ensemble

In our simulations, we fix the volume trapped between the membrane and the substrate. Even if this is the case, a protrusion can exchange volume with its surroundings by taking up or expelling fluid into the interstitial space between the adhered part of the membrane and the substrate. The ease to do so is controlled by the adhesion potential $U(z)$, which once the protrusion has nucleated, has little influence on other aspects of the model. We varied the adhesion energy $U(z_0)$ as indicated in Supplementary Table 1, and additionally, considered a fixed pressure ensemble, facilitating the exchange of trapped fluid volume of the protrusion.

8.3 Sensitivity to size and mechanical ensemble

Most simulations were performed with a reference mechanical ensemble given by the adhesion energy density $U(z_0) = 1.5$ mJ/m^2 and $k_{\text{spring}} = 0$ (fixed tension), unless otherwise stated, and performed a number of simulations varying the area and volume ensemble to examine its effect. In general terms, we found that the size of buds and tubes or of mechanical ensemble had no effect on the mechanisms of reshaping for buds and tubes described in the main text. In all cases, the former developed high coverage and nematic order at the neck, which elongated into a tube connecting the vesicle to the supported membrane, whereas the latter developed a pearling instability at relatively low coverage, which enabled enrichment, nematic order, and elongation at the necks. As shown in Supplementary Figure 10 and further quantified in Supplementary Figure 7c, the area/tension ensemble had very little quantitative effect on our simulations, whereas volume confinement required slightly higher nominal concentrations and coverage for reshaping to occur. The only qualitative exception was the case of fixed pressure and fixed strain for tubes. In this case, tubes uniformly elongated without exhibiting pearling followed by the tube-bud coexistence, Supplementary Figure 11. This shows that some degree of volume confinement is required for the experimentally observed sequence of events of tube reshaping. We found that if volume confinement was too large, the bud did not significantly shrink during neck elongation as the volume inside the small tube is very small. Instead, the bud shrunk when volume exchange was eased (smaller value of $U(z_0)$), Supplementary Movie 8. Overall and despite these differences of behavior, our results were quite insensitive to the protrusion size and mechanical ensemble.

9 Effect of thermal fluctuations

Thermal fluctuations are implicitly accounted for in some of the aspect of our theory, such as the entropic free energy of proteins on the membrane or the effective mechanical properties of the membrane. Out-of-

plane membrane fluctuations are certainly present in our low-tension membrane protrusions, and should renormalize some of our model effective parameters, such as adsorption/desorption rates, although their relatively small size will restrict the available mode numbers and amplitudes. Experimentally, the most notorious fluctuations are shape fluctuations of thin tubules, which result in ostensively non-symmetric shapes. To assess the effect of such fluctuations on tubule strain and coverage fluctuations induced by tube bending, we noted that for a membrane tube of radius r, the persistence length is given by[25]

$$\ell_p = \frac{\pi r k_0}{k_B T} \approx 20\pi r, \tag{20}$$

for a bare lipid tubule, and higher for a stiffer coated tubule. Thermal fluctuations induce curvature fluctuations in the order of the inverse of the persistence length[26]. For a tubule of radius $r = 100$ nm, we estimate the persistence length to be of about $\ell_p \approx 6$ μm, whereas for $r = 20$ nm we find $\ell_p \approx 1$ μm, which is comparable to the radii of curvature observed in our experiments. According to these estimates, the relative local areal change in the inner/outer parts of the bent tube can be estimated as $\pm 1/(20\pi)$, which should lead to protein density fluctuations of less than 2%. We thus can assume that these shape changes will have a mild effect on the dynamics of protein adsorption and transport and on the mechanics of the membrane. Membrane fluctuations have also been shown to be important during symmetry-breaking self-organizations of BAR proteins on nominally flat membranes[27]. In most reshaping and molecular reorganization events described here, the initial membrane template strongly directs the process, e.g. the thin neck of buds, and therefore, we expect a less acute effect of thermal fluctuations. However, the pearling instability involved in the reshaping of tubes breaks symmetry, and hence, fluctuations may influence the threshold for this shape transition.

Supplementary figures

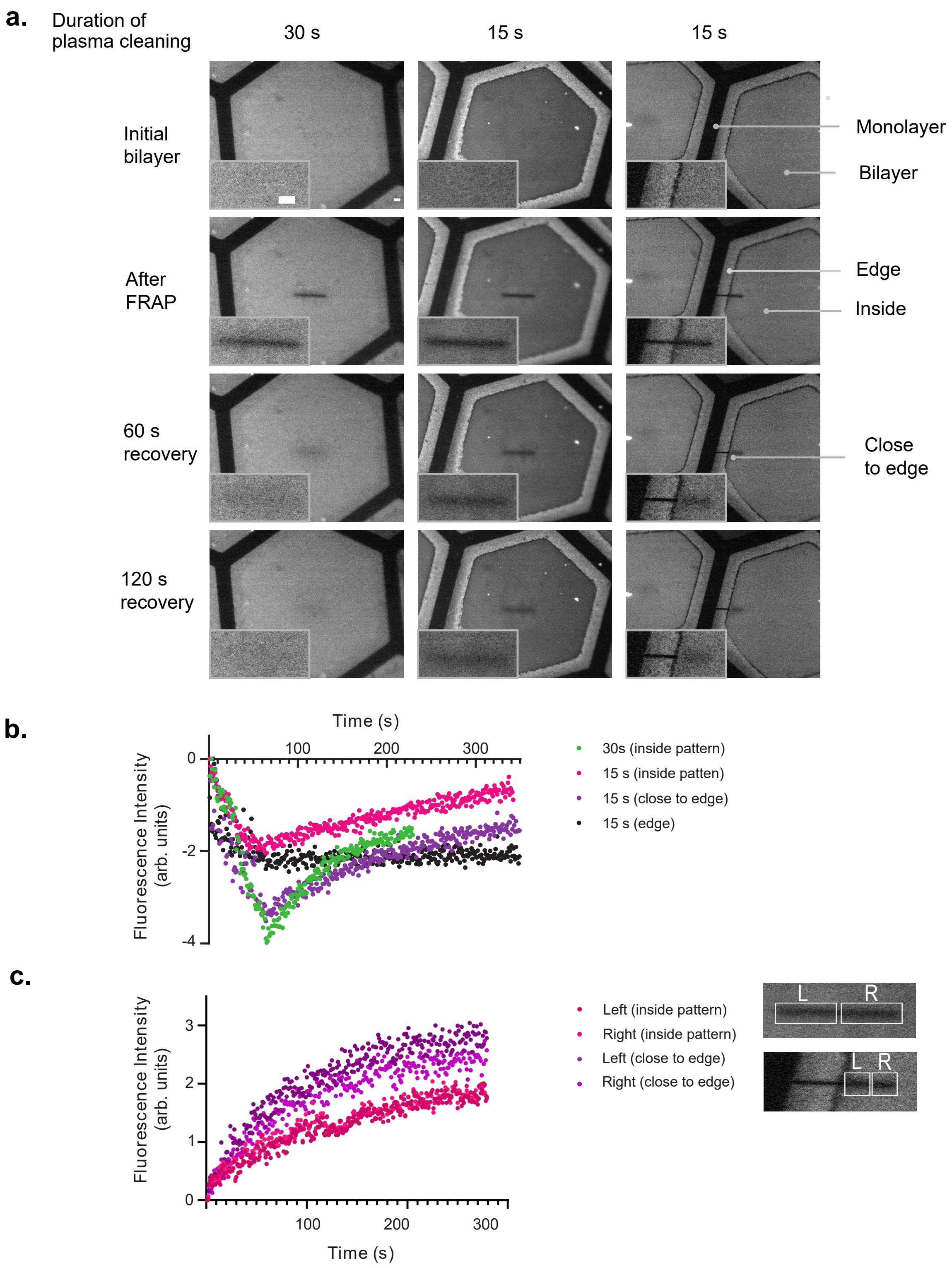

Supplementary Figure 1: Characterization of membrane fluidity.

a, Time-lapse images of the Patterned Supported Lipid Bilayers (pSLB) obtained after photobleaching a line. Results after different plasma cleaning times of the PDMS membrane are shown. Shorter plasma

cleaning time (15 s) lead to more liposomes sitting on top of the bilayer and at the edge of the hexagon. Fluorescent recovery after photobleaching (FRAP) experiments show that both pSLBs (15 s or 30 s plasma cleaning times) are fluid compared with the non-fluid border (right images). Scale bar, 5 μm. **b**, Recovery curves of the frapped areas of several pSLBs obtained either with a 15 s or a 30 s plasma cleaning time. Recovery is slower with a shorter plasma cleaning time, indicating a lower membrane fluidity. The edge does not show recovery. Source data are provided as a Source Data file. **c**, Recovery curves of the frapped areas of two pSLBs obtained either with a 15 s plasma cleaning time, where the left (L) and right (R) parts of the bleached line are quantified separately. Source data are provided as a Source Data file.

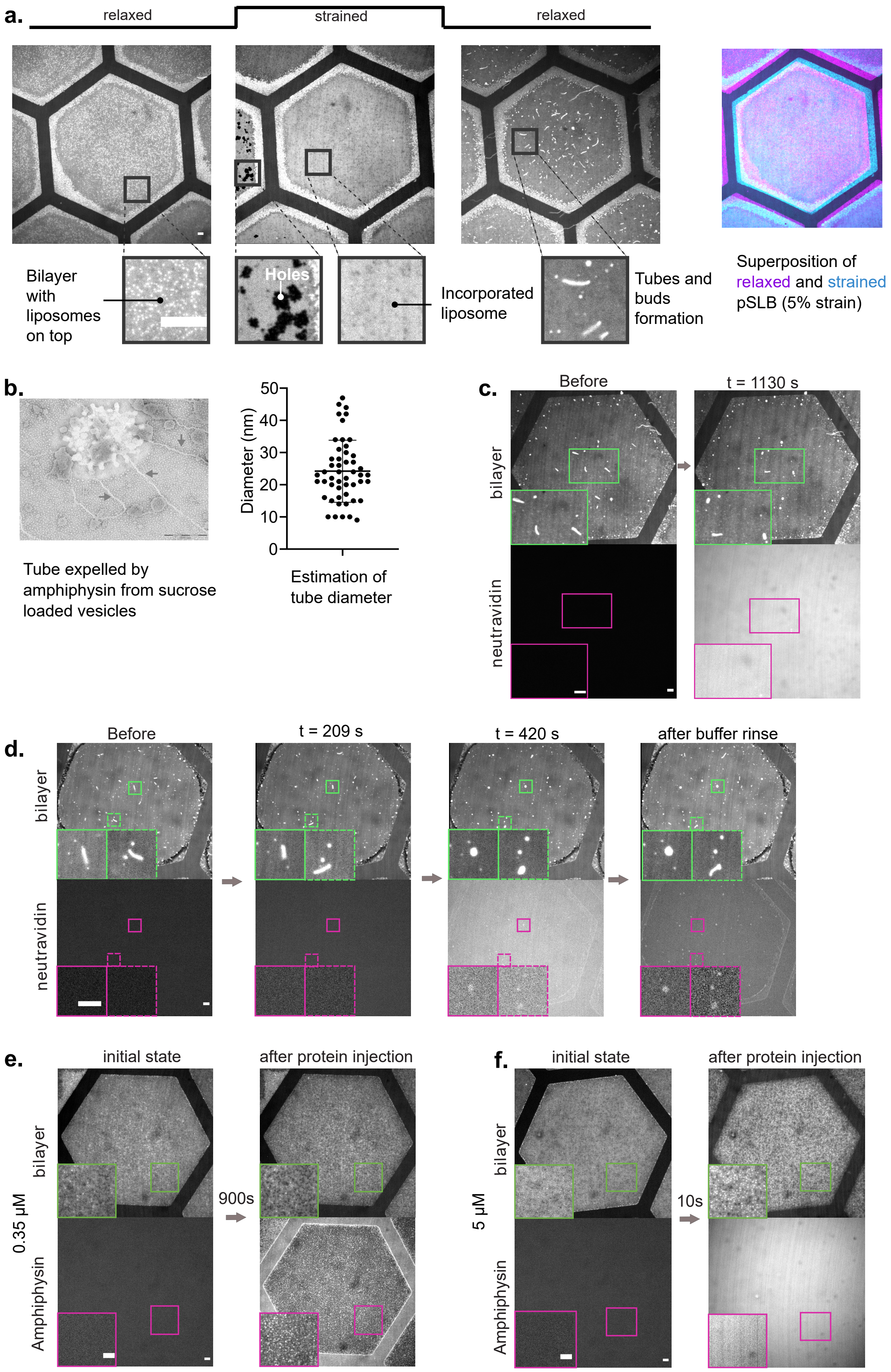

Supplementary Figure 2: Additional characterization of membrane reshaping.

a, (Left) Detailed example of the formation process of tubes and buds. At the relaxed initial state, liposomes stand in excess on top of the Patterned Supported lipid Bilayer (pSLB). With strain, the liposomes incorporate in the pSLB, and if not enough excess lipid is present, holes are formed and the naked PDMS membrane is exposed (dark holes). Upon release, excess lipids are expelled in the form or tubes or buds. (Right) Superposition of the relaxed bilayer (magenta) and strained bilayer (cyan). **b**, Estimation of the diameter of tubes reshaped by Amphiphysin using vesicles incubated with the protein and subsequently observed by transmission electron microscopy (TEM). Data are shown as mean ± s.d. Source data are provided as a Source Data file. **c**, Control in which 1 μM fluorescent Neutravidin is injected on top of the pSLB. The buds remain intact and the tubes slowly relax to buds, but no reshaping in the form of thin tubes is observed. **d**, Control in which 1 μM fluorescent Neutravidin is injected on top of the pSLB containing 1% biotinylated-PE. The buds remain intact and the tubes slowly relax to buds, but no reshaping in the form of thin tubes is observed. As the tubes are moving, they may attach to the bilayer (second panel) and readily confirm the tube-shape of such structures. **e, f,** Example of Amphiphysin injected on top of a non-stimulated pSLB at low (e) and very high (f) concentrations. At low concentration, no major effect is observed. At very high concentration, the bilayer is teared by the protein, leading to an immediate pSLB reshaping, in the form of bright dots and black holes. Scale bars, 5 μm.

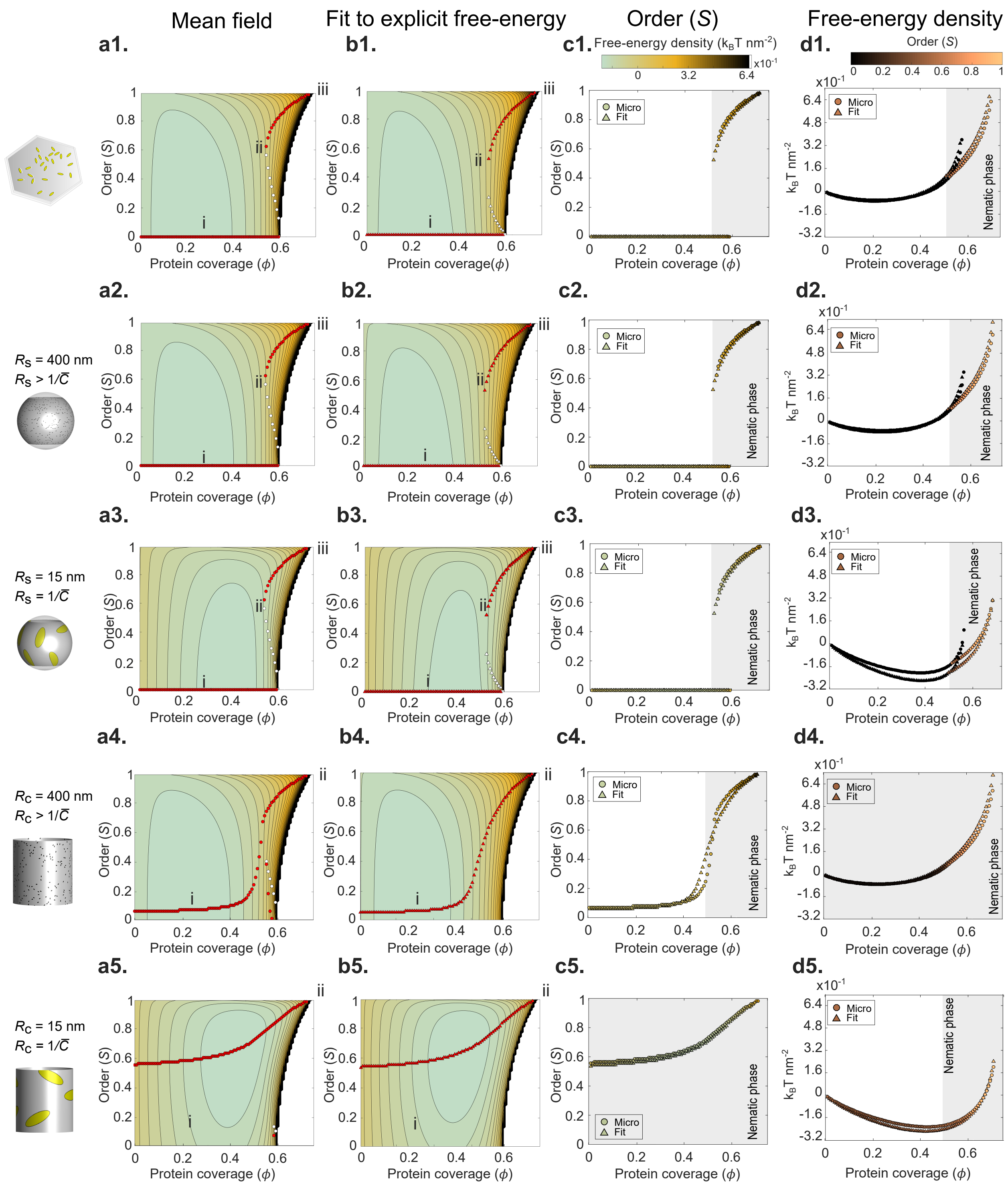

Supplementary Figure 3: Explicit parametrization of the mean field model.

a, Landscape of free-energy density per unit area computed with the mean field model in Eq. (12) in the Theoretical Model and described in detail in [51] for membranes of different curvature (flat, spherical and cylindrical with radii larger or equal to the intrinsic radius of a protein $1/\bar{C}$). **b**, Analogous landscapes of the free-energy density with the explicit model given by Eqs. (13,16) fitted to the mean field model. This explicit approximation $\hat{\mathcal{F}}_{\text{prot}} = \hat{\mathcal{F}}_{\text{prot,entropy}} + \hat{\mathcal{F}}_{\text{prot,curv}}$ is amenable to numerical calculations, see Supplementary Note 1. By minimizing the free-energy density with respect to S for a given protein covera-

ge ϕ we find equilibrium paths $\phi(S)$. Stable branches are marked with red dots and unstable ones by white dots in (a) and (b). **c**, Comparison of the stable branches in the $\phi - S$ plane with both models (color is free-energy density). **d**, Comparison of the stable branches in the ϕ - energy plane with both models (color is order).

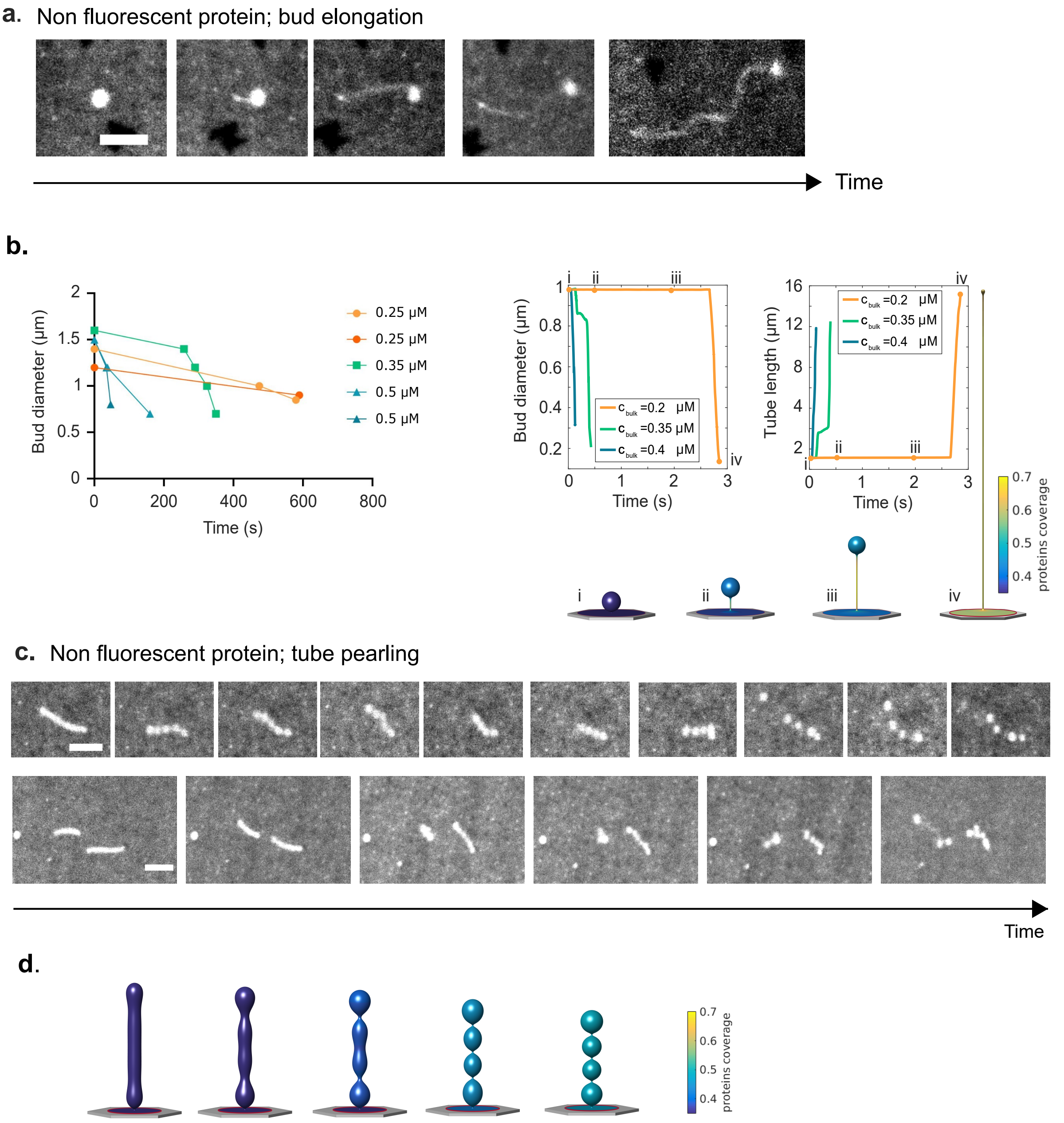

Supplementary Figure 4: Additional data regarding bud and tube reshaping.

a, Example of a bud elongated by non-fluorescent Amphiphysin upon 300 nM protein injection. Scale bars, 5 μm. **b**, (Left) Experimental quantification of bud diameter over time during elongation at different concentrations of Amphiphysin in the bulk. Source data are provided as a Source Data file. (Right) Theoretical prediction of bud diameter as buds elongate for a mechanical ensemble enabling easy volume exchange and no area exchange, $(U(z_0) = 0.075$ mJ/m^2 and $k_{spring} = +\infty$, that is fixed projected area) **c**, Example of a tube undergoing the pearling phase due to binding of non-fluorescent Amphiphysin (300 nm protein injection). Scale bars, 5 μm. **d**, Membrane reshaping for isotropic spontaneous curvature, simulated by prescribing isotropic orientational order, S=0, recovering the classical pearling instability but no further reshaping associated to nematic ordering.

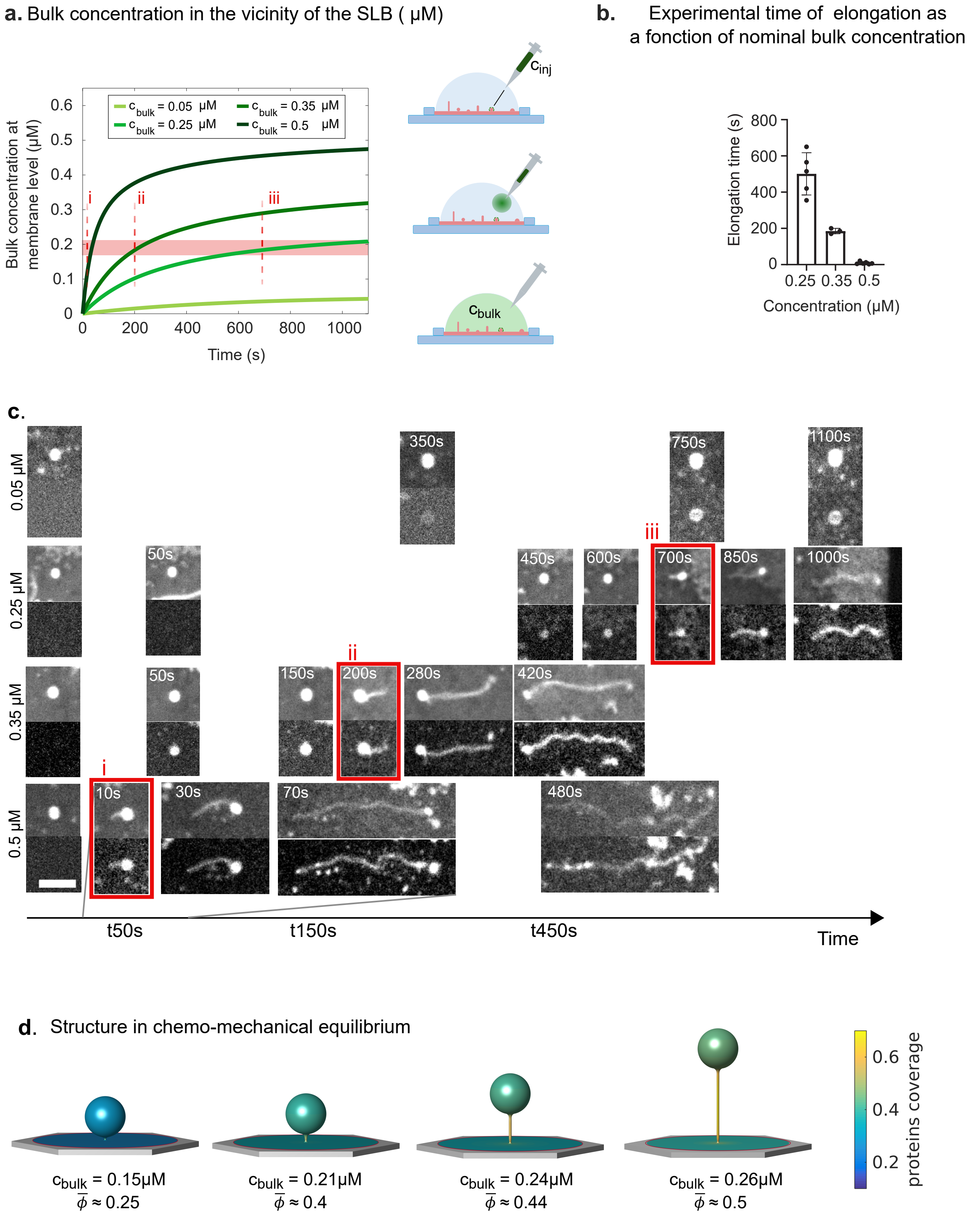

Supplementary Figure 5: Time evolution of bud reshaping is governed by bulk diffusion.

a, 1D protein diffusion in the bulk to simulate the experimental injection setup. The experimental time-instants when bud elongation starts for several nominal concentrations in the experiments (red dashed lines), see (c), are well-predicted by the theory as the instants when the bulk concentration required for bud

elongation in the numerical simulations (red-shaded region) is reached in the vicinity of the SLB by bulk diffusion. **b**, Times at which bud elongation starts as a function of concentration (n=5,3 and 6 for 0.25, 0.35 and 0.5 μM respectively). Data are shown as mean ± s.d. Source data are provided as a Source Data file. **c**, Experimental examples of buds reshaped at different nominal concentrations of Amphiphysin in the bulk. Pearling and elongation occur faster at higher concentration. Scale bar, 5 μm. **d**, Quasi-equilibrium states at increasing bulk concentration.

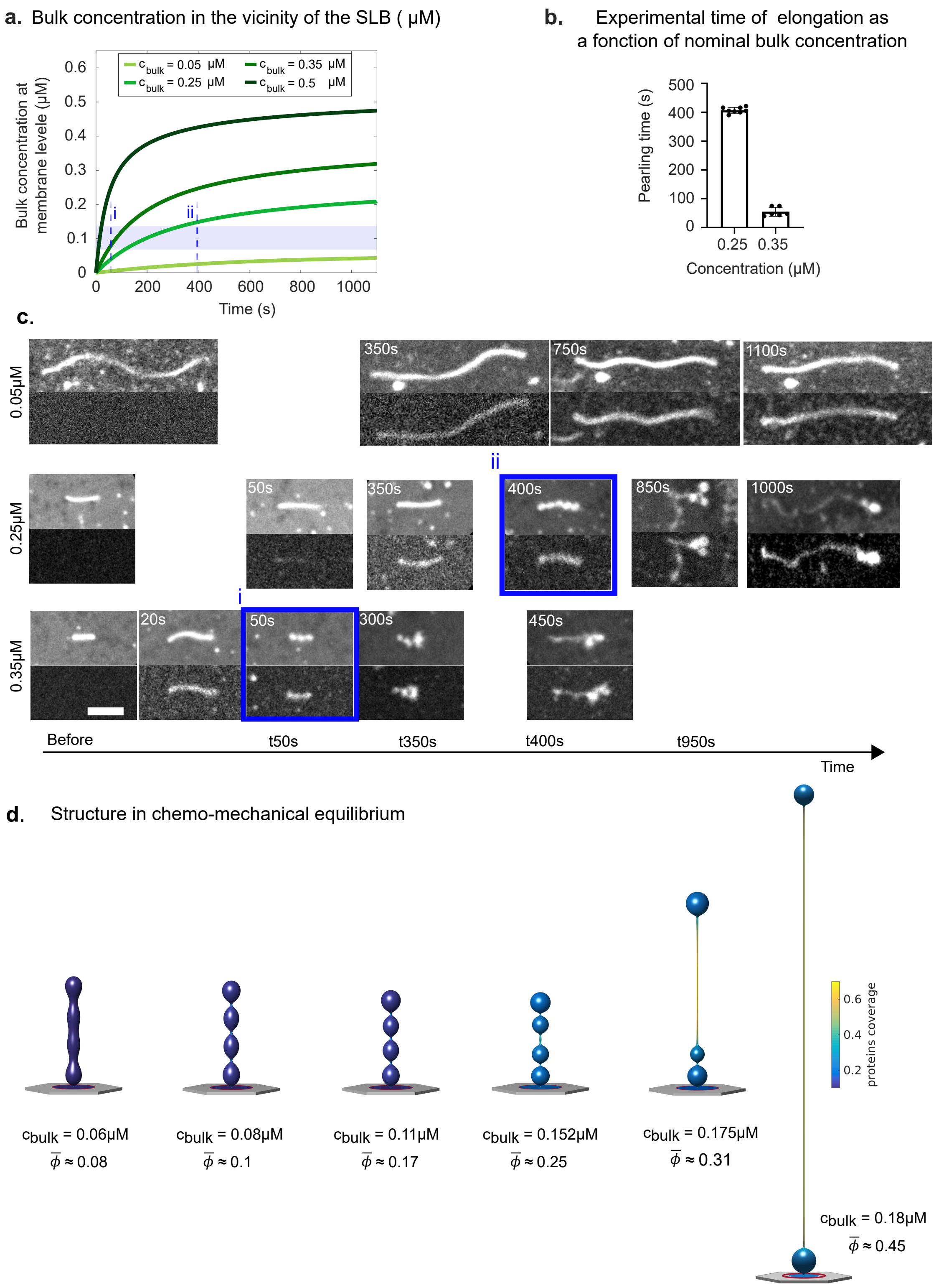

Supplementary Figure 6: Time evolution of tube reshaping is governed by bulk diffusion.

a, 1D protein diffusion in the bulk to simulate the experimental injection setup. The experimental time-instants when tube pearling starts for several nominal concentrations in the experiments (blue dashed lines), see (c), are well-predicted by the theory as the instants when the bulk concentration required for pearling in the numerical simulations (blue-shaded region) is reached in the vicinity of the SLB by bulk

diffusion. **b**, Times at which tube pearling starts as a function of concentration (n=5 and 6 for 0.25 and 0.35 μM respectively). Data are shown as mean ± s.d. Source data are provided as a Source Data file. **c,**. Experimental examples of tubes reshaped at different concentrations of Amphiphysin in the bulk. Elongation occurs faster at higher concentration. Scale bar, 5 μm. **d**, Quasi-equilibrium states at increasing bulk concentration.

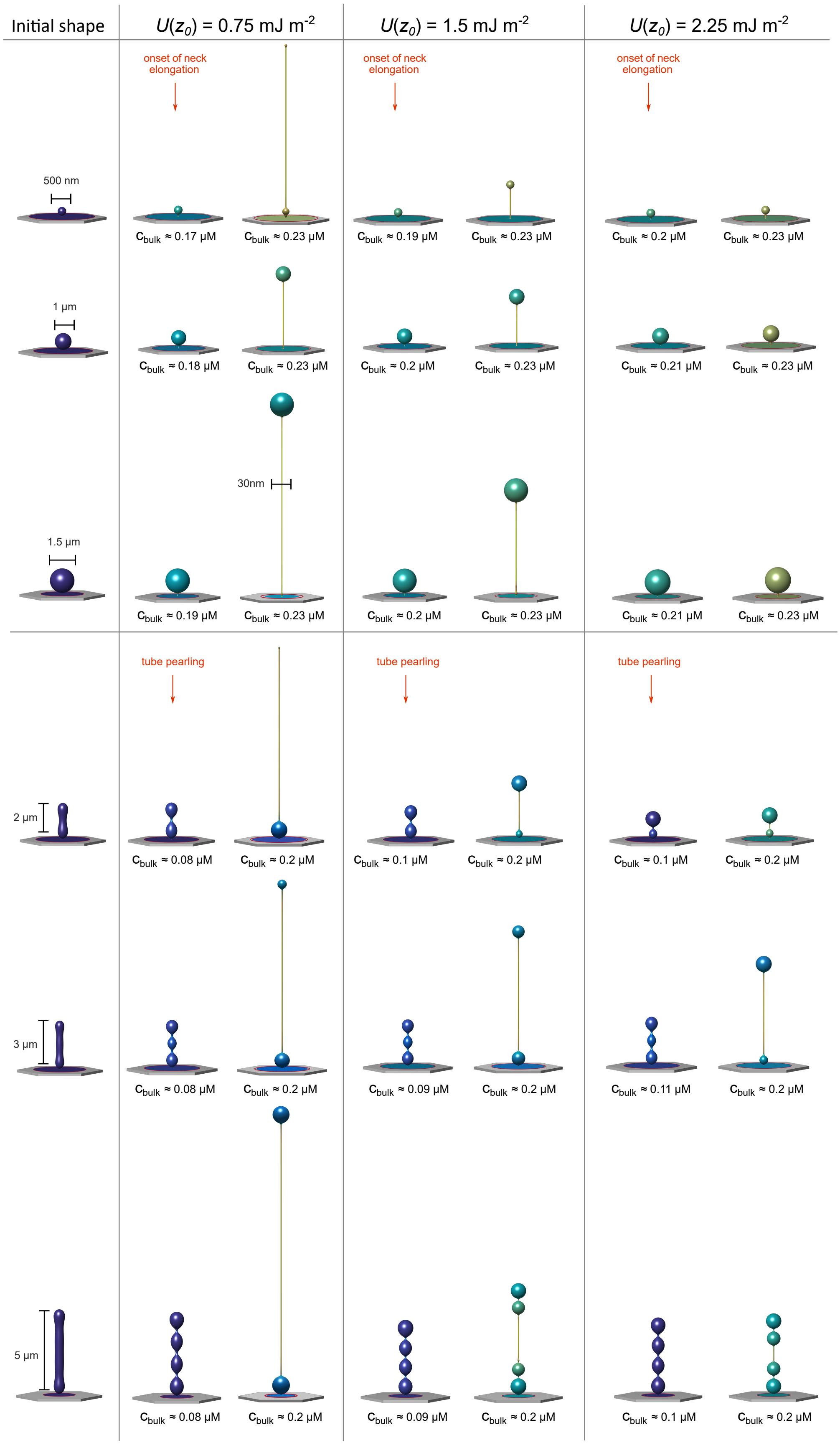

Supplementary Figure 7: Reshaping in response to different Amphiphysin bulk solutions for protrusions of different size and for various adhesion energies.

Representative simulations used in the sensitivity plots shown in Supplementary Figure 9, where in addition to the size of the protrusion and the adhesion energy density $U(z_0)$ (controlling the volume ensemble) shown here, we varied the membrane area ensemble (here we consider $k_{\text{spring}} = 0.32$ mN/nm^2). The figure shows equilibrium states of buds and tubes at different bulk concentrations, showing that while the reshaping mechanism is the same irrespective of size and ensemble, the bulk concentration required for reshaping is higher when adhesion energy is larger.

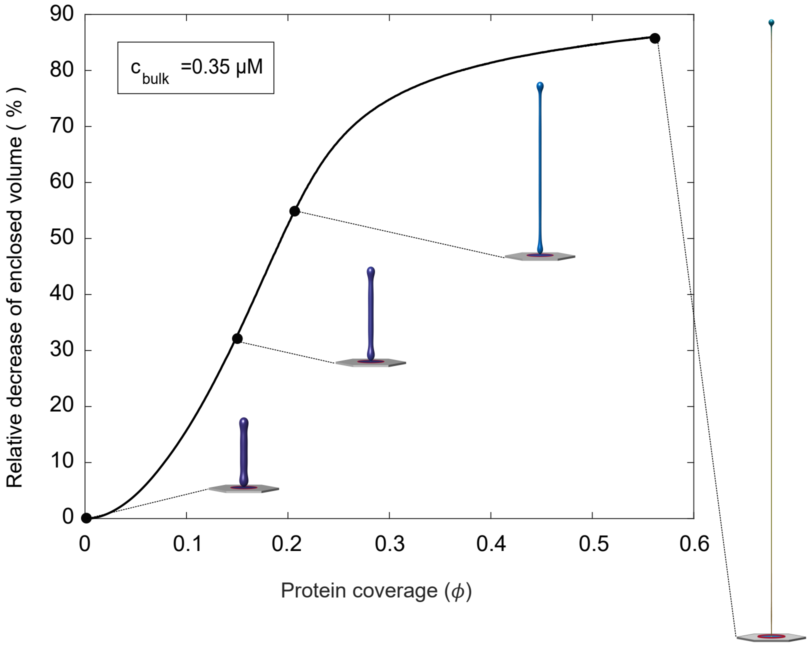

Supplementary Figure 8: Tube elongation in conditions where volume exchange is facilitated.

Tube reshaping in response to Amphiphysin under the condition of fixed pressure across the membrane and fixed projected area (no area exchange). The plot shows the enclosed volume decrease during the dynamics as a function of protein coverage, along with representative snapshots of the shape of the protrusion, showing that in conditions of easy volume exchange, the reshaping does not proceed by pearling and mixture of states.

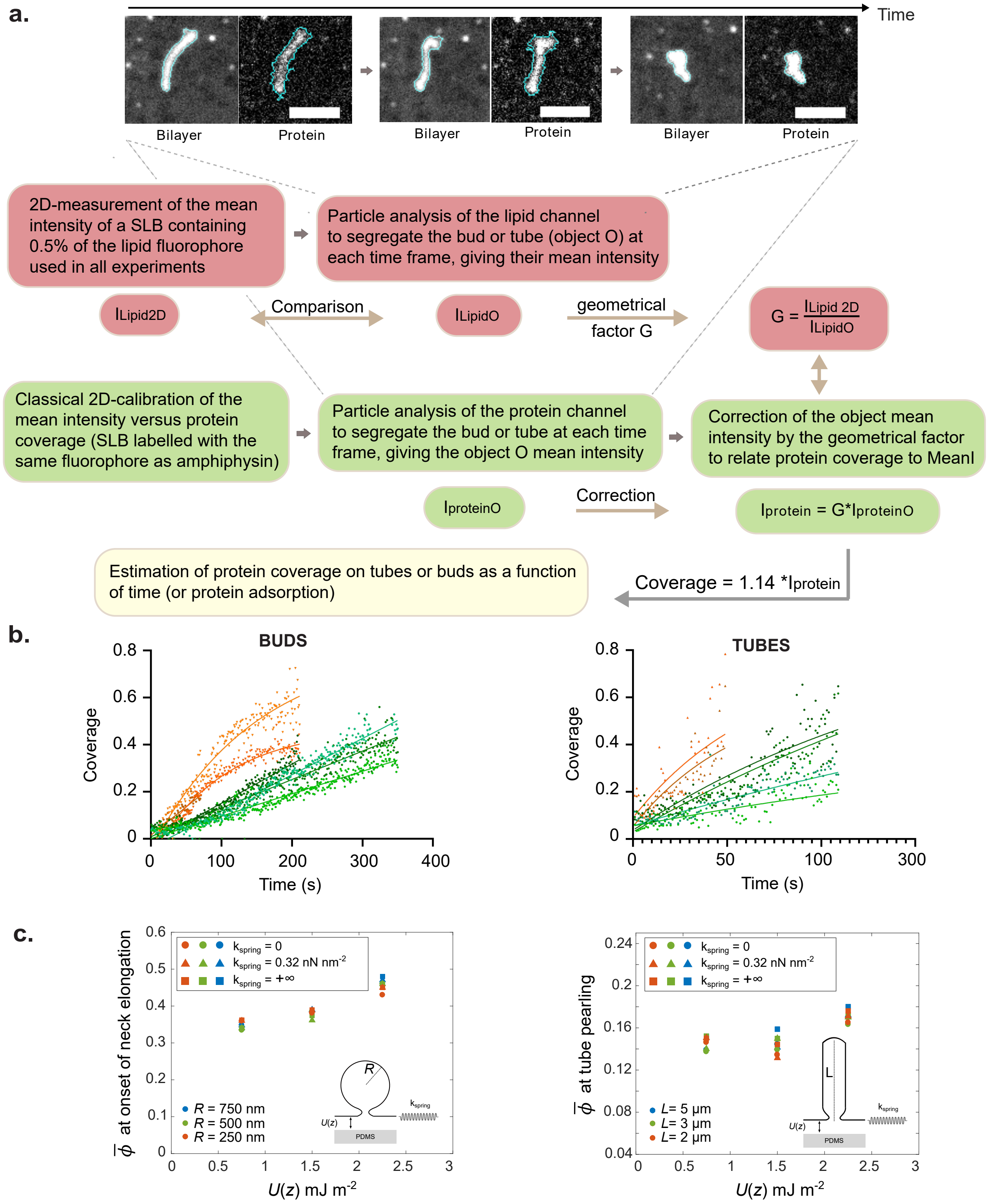

Supplementary Figure 9: Estimations of protein coverage.

a, (Top) Representative images of the particle analysis performed at each time frame in both fluorescence channels (contour of the particle in cyan). Scale bar, 5 µm. (Bottom) Protocol followed to estimate the protein coverage on the tubes or buds over time, in order to correct for the geometry of lipid structures.

b, Binding curves of the protein binding to several buds (left) or tubes (right) at 0.25 μM (green colours) or 0.35 μM (orange colours) bulk protein concentration. The protocol described in (a) has been used, enabling to plot the protein coverage on tubes or buds over time. Tube elongation from buds (left) starts when the plot ends, tube pearling (right) starts when the plot ends. Source data are provided as a Source Data file. **c**, (Left) Protein coverage at which bud elongation starts as a function of membrane-support adhesion energy $U(z_0)$, for different the membrane area ensembles (different values of k_{spring}) and buds of different radius. (Right) Analogous plot for protein coverage at which tube pearling is observed for a tubes of different lengths and radius $\approx$ 300 nm. Source data are provided as a Source Data file.

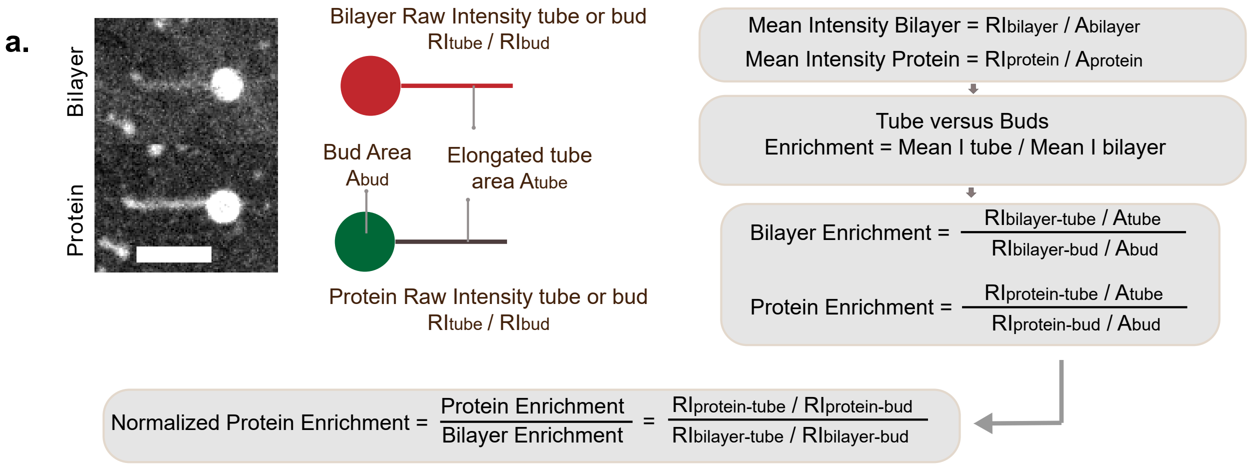

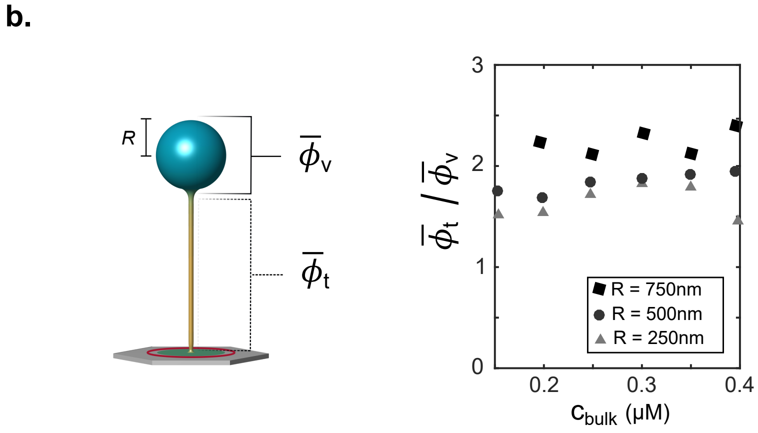

Supplementary Figure 10: Estimation of Amphiphysin enrichment.

a, Quantification of protein enrichment on the elongating tube. Scale bar 5 μm. (Left) Examples of bilayer and protein fluorescence images of an elongating tube. Raw intensities on the tube and bud are measured (and corrected from background) in both lipid and protein images at the same timepoint. (Right) Protein enrichment on the tube versus bud is defined as the ratio between the mean protein fluorescence intensity levels in tubes and buds. However, calculating mean protein intensities require calculating membrane areas, which is challenging. To circumvent this, we assume that real enrichment in the membrane bilayer channel is 1, that is, the concentration of membrane is the same in both tubes and buds. Then, we factor out membrane areas by normalizing enrichment in the protein channel by the same value in the membrane bilayer channel. **b**, Computational estimation of relative protein enrichment between mean coverage on the tube ($\bar{\phi}_t$) and mean coverage on the vesicle ($\bar{\phi}_v$) for buds of different sizes exposed to different protein concentrations. Source data are provided as a Source Data file.

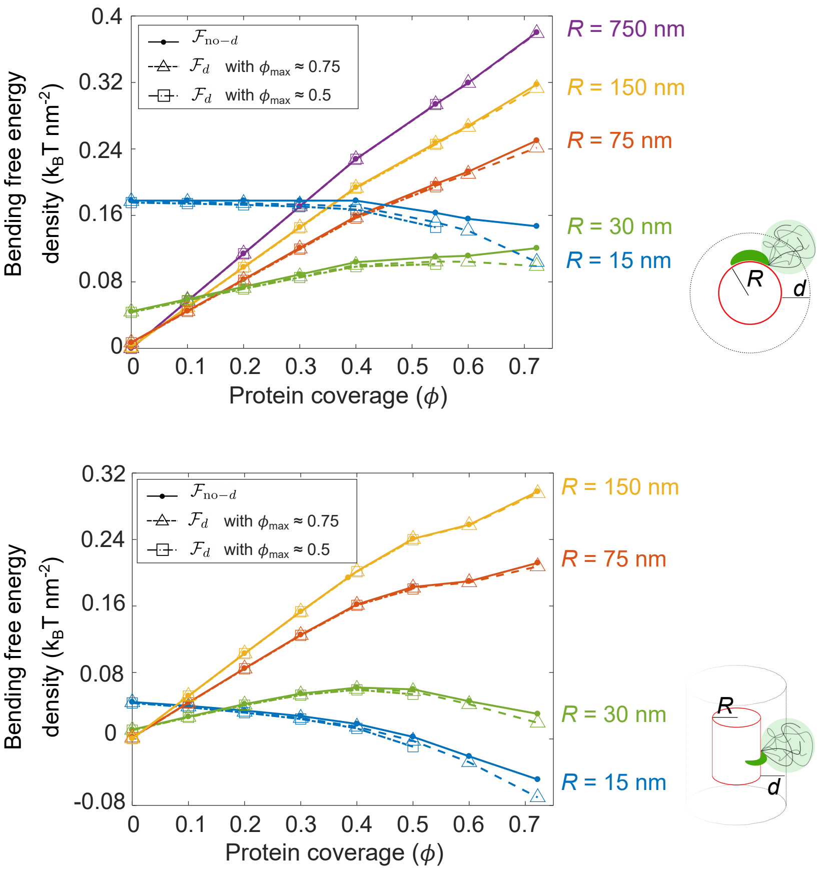

Supplementary Figure 11: Effect of bulky disordered domains.

Bending free-energy density on spheres (top) and tubes (bottom) of different radii as a function of protein coverage, without the curvature coupling caused by bulky disordered domains (continuous lines) and with this effect (dashed lines for two values of $\phi_{\max}$, see Supplementary Note 1). Source data are provided as a Source Data file.

Supplementary References

[1] Tozzi, C., Walani, N. & Arroyo, M. Out-of-equilibrium mechanochemistry and self-organization of fluid membranes interacting with curved proteins. New J. Phys.21, (2019).

[2] Tozzi, C., Walani, N., Le Roux, A. L., Roca-Cusachs, P. & Arroyo, M. A theory of ordering of elongated and curved proteins on membranes driven by density and curvature. Soft Matter17, 3367–3379 (2021).

[3] Arroyo, M., Walani, N., Torres-Sánchez, A. & Kaurin, D. Onsager's variational principle in soft matter: Introduction and application to the dynamics of adsorption of proteins onto fluid membranes. in CISM International Centre for Mechanical Sciences, Courses and Lectures577, 287–332 (2018). Proc. Natl. Acad. Sci. U. S. A.109, 173–178 (2012).

[4] Staykova, M., Arroyo, M., Rahimi, M. & Stone, H. A. Confined bilayers passively regulate shape and stress. Phys. Rev. Lett.110, 1–5 (2013).

[5] Isas, J. M., Ambroso, M. R., Hegde, P. B., Langen, J. & Langen, R. Tubulation by amphiphysin requires concentration-dependent switching from wedging to scaffolding. Structure23, 873–881 (2015).

[6] Prévost, C., Tsai, F. C., Bassereau, P. & Simunovic, M. Pulling membrane nanotubes from giant unilamellar vesicles. J. Vis. Exp.2017, 1–2 (2017).

[7] Ayton, G. S. et al. New insights into BAR domain-induced membrane remodeling. Biophys. J.97, 1616–1625 (2009).

[8] Kaurin, D. & Arroyo, M. Surface tension controls the hydraulic fracture of adhesive interfaces bridged by molecular bonds. Phys. Rev. Lett.123, (2019).

[9] Feng, J., Chaubal, C. V. & Leal,L. G. Closure approximations for the Doi theory: Which to use in simulating complex flows of liquid-crystalline polymers? J. Rheol. (N. Y. N. Y).42, 1095–1119 (1998).

[10] Noguchi, H. Membrane tubule formation by banana-shaped proteins with or without transient network structure. Sci. Rep.6, (2016).

[11] Bonazzi, F. & Weikl, T. R. Membrane Morphologies Induced by Arc-Shaped Scaffolds Are Determined by Arc Angle and Coverage. Biophys. J.116, 1239–1247 (2019).

[12] Simunovic, M. et al. How curvature-generating proteins build scaffolds on membrane nanotubes. Proc. Natl. Acad. Sci. U. S. A.113, 11226–11231 (2016).

[13] Sorre, B. et al. Nature of curvature coupling of amphiphysin with membranes depends on its bound density.

[14] Dimova, R. et al.A practical guide to giant vesicles. Probing the membrane nanoregime via optical microscopy. J. Phys. Condens. Matter18, (2006).

[15] https://www.rcsb.org/structure/3SOG (online repository for molecular stuctures).

[16] Evans, E. & Needham, D. Physical properties of surfactant bilayer membranes: Thermal transitions, elasticity, rigidity, cohesion, and colloidal interactions. Journal of Physical Chemistry91, 4219–4228 (1987).

[17] Simunovic, M., Prévost, C., Andrew, C. J. & Bassereau, P. Physical basis of some membrane shaping mechanisms. Philosophical Transactions of the Royal Society A: Mathematical, Physical and Engineering Sciences374, (2016).

[18] Prévost, C. et al.IRSp53 senses negative membrane curvature and phase separates along membrane tubules. Nat. Commun.6, (2015).

[19] Noguchi, H. Formation of polyhedral vesicles and polygonal membrane tubes induced by banana-shaped proteins. J. Chem. Phys.143, (2015).

[20] Larsen, J. B. et al. How Membrane Geometry Regulates Protein Sorting Independently of Mean Curvature. ACS Cent. Sci.6, 1159–1168 (2020).

[21] Rahimi, M. & Arroyo, M. Shape dynamics, lipid hydrodynamics, and the complex viscoelasticty of bilayer membranes. Phys. Rev. E -Stat. Nonlinear, Soft Matter Phys.86, (2012).

[22] Zeno, W. F. et al. Synergy between intrinsically disordered domains and structured proteins amplifies membrane curvature sensing. Nat.Commun.9, (2018).

[23] Lipowsky, R. Bending of membranes by anchored polymers. Epl30, 197–202 (1995).

[24] Busch, D. J. et al. Intrinsically disordered proteins drive membrane curvature. Nat. Commun.6, (2015).

[25] Ramakrishnan, N., Sunil Kumar, P. B. & Ipsen, J. H. Membrane-mediated aggregation of curvature-inducing nematogens and membrane tubulation.Biophys. J.104, 1018–1028 (2013).

[26] Wisanpitayakorn, P., Mickolajczyk, K. J., Hancock, W. O., Vidali, L. & Tüzel, E. Measurement of the Persistence Length of Cytoskeletal Filaments using Curvature Distributions. Biophys. J.112, 566a (2017).

[27] Simunovic, M. & Voth, G. A. Membrane tension controls the assembly of curvature-generating proteins. Nat. Commun.6, 1–8 (2015).
