# Supplementary material for: Dynamic mechanochemical feedback between curved membranes and BAR protein self-organization
Source: Nat Commun. 2021 Nov 12;12:6550. doi: 10.1038/s41467-021-26591-3 (PMC8589976; doi:10.1038/s41467-021-26591-3)
Supplement: Supplementary file 3 — Description and legends of supplementary files [file 41467_2021_26591_MOESM3_ESM.docx]

**Description of Additional Supplementary Files**

**File Name:** Supplementary Movie 1

**Description:** Time sequence of the patterned Supported Lipid Bilayer (pSLB) slowly destretching from 5 % strain to 0 %, leading to the formation of lipid tubes. The pSLB composition is DOPC:DOPS:DOPA 3:2:1, supplemented with 0.5 % mol LissRhod-DPPE. Images show the pSLB fluorescent lipid. The hexagonal region drawn in green shows the initial size of the stretched pattern. Note that some defocused frames are kept for the sake of timelapse understanding; as destretch occurs the PDMS membrane moves in the vertical direction and refocusing is required.

**File Name:** Supplementary Movie 2

**Description:** Short time sequence of the lipid tubes and buds formed through pSLB destretch. Images show the pSLB membrane marker. Inset shows a magnification of the region marked with a green square.

**File Name:** Supplementary Movie 3

**Description:** Longer time sequence of the lipid tubes and buds formed through pSLB destretch. The pSLB composition is DOPC:DOPS:DOPA 3:2:1, supplemented with 0.5 % mol LissRhod-DPPE. Note that tubes relax to buds with time. Images show the pSLB fluorescent lipid. Inset shows a magnification of the region marked with a green square.

**File Name:** Supplementary Movie 4

**Description:** Time sequence of the lipid tubes and buds formed through pSLB destretch before and after incubation with 1 μM fluorescent Neutravidin. The pSLB composition is DOPC:DOPS:DOPA 3:2:1, supplemented with 0.5 % mol LissRhod-DPPE. Images show the pSLB fluorescent lipid (left) and fluorescent Neutravidin marker (right). Insets show magnifications of the regions marked with a square.

**File Name:** Supplementary Movie 5

**Description:** Time sequence of the lipid tubes and buds formed through pSLB destretch before and after incubation with 0.25 μM fluorescent Neutravidin. The pSLB composition is DOPC supplemented with 5 % mol of Cap-Biotin. Images show the pSLB fluorescent lipid (left) and fluorescent Neutravidin (right). Insets show magnifications of the regions marked with a square.

**File Name:** Supplementary Movie 6

**Description:** Time sequence of a non-stretched pSLB before and after incubation with 0.25 μM fluorescent Amphiphysin. A~~a~~mphiphysin. The pSLB composition is DOPC:DOPS:DOPA 3:2:1, supplemented with 0.5 % mol LissRhod-DPPE. Images show the pSLB fluorescent lipid (left) and fluorescent Amphiphysin (right). Insets show magnifications of the regions marked with a square.

**File Name:** Supplementary Movie 7

**Description:** Time sequence of a non-stretched pSLB before and after incubation with 5 μM fluorescent Amphiphysin. The pSLB composition is DOPC:DOPS:DOPA 3:2:1, supplemented with 0.5 % mol LissRhod-DPPE. Images show the fluorescent lipid (note that to inject the protein at high concentration, the non-fluorescent Amphiphysin form was used). Inset shows magnifications of the regions marked with a green square.

**File Name:** Supplementary Movie 8

**Description:** Numerical simulation of the reshaping of a lipid bud of initial diameter 1.5 μm following protein binding from a protein bulk concentration of 0.35 μM. Color is protein coverage (left) and order (right). Membrane tension is fixed to that prior to protein exposure and the volume enclosed between the membrane and the substrate is fixed.

**File Name:** Supplementary Movie 9

**Description:** Time sequence of the reshaping of lipid tubes and buds formed through pSLB destretch, before and after incubation with 0.5 μM Amphiphysin. The pSLB composition is DOPC:DOPS:DOPA 3:2:1, supplemented with 0.5 % mol LissRhod-DPPE. Images show the fluorescent lipid (left) and fluorescent Amphiphysin (right). Insets show magnifications of the regions marked with squares.

**File Name:** Supplementary Movie 10

**Description:** Time sequence of the reshaping of lipid tubes and buds formed through pSLB destretch, after incubation with 0.25 μM Amphiphysin. The pSLB composition is DOPC:DOPS:DOPA 3:2:1, supplemented with 0.5 % mol LissRhod-DPPE. Images show the fluorescent lipid (left) and fluorescent Amphiphysin (right). Insets show magnifications of the regions marked with squares.

**File Name:** Supplementary Movie 11

**Description:** Time sequence of the reshaping of a lipid bud formed through pSLB destretch, after incubation with 0.3 μM non-fluorescent Amphiphysin. The pSLB composition is DOPC:DOPS:DOPA 3:2:1, supplemented with 0.5 % mol LissRhod-DPPE. Images show the pSLB fluorescent lipid. Insets show magnifications of the regions marked with squares.

**File Name:** Supplementary Movie 12

**Description:** Numerical simulation of the reshaping of a lipid bud of initial diameter 1 μm following protein binding from a protein bulk concentration of 0.35 μM. Color is protein coverage (left) and order (right). Membrane tension is fixed to that prior to protein exposure and the exchange of the volume enclosed by the protrusion is eased by considering a softer substrate interaction with U(z_0_) = 0.075 mJ/m^2^.

**File Name:** Supplementary Movie 13

**Description:** Numerical simulation of the reshaping of a lipid tube of initial diameter 0.6 μm following protein binding from a bulk concentration of 0.35 μM. Color is protein coverage (left) and order (right). Membrane tension is fixed to that prior to protein exposure and the volume enclosed between the membrane and the substrate is fixed.

**File Name:** Supplementary Movie 14

**Description:** Time sequence of the reshaping of lipid tubes and buds formed through pSLB destretch, before and after incubation with 0.25 μM Amphiphysin. The pSLB composition is DOPC:DOPS:DOPA 3:2:1, supplemented with 0.5 % mol LissRhod-DPPE. Images show the fluorescent lipid (left) and fluorescent Amphiphysin (right). Insets show magnifications of the regions marked with squares.

**File Name:** Supplementary Movie 15

**Description:** Time sequence of the reshaping of lipid tubes and buds formed through pSLB destretch, before and after incubation with 0.35 μM Amphiphysin. The pSLB composition is DOPC:DOPS:DOPA 3:2:1, supplemented with 0.5 % mol LissRhod-DPPE. mages show the fluorescent lipid (left) and fluorescent Amphiphysin (right). Insets show magnifications of the regions marked with squares.

**File Name:** Supplementary Movie 16

**Description:** Time sequence of the reshaping of three lipid tubes formed through pSLB destretch, after incubation with 0.3 μM non-fluorescent Amphiphysin. The pSLB composition is DOPC:DOPS:DOPA 3:2:1, supplemented with 0.5 % mol LissRhod-DPPE. Images show the pSLB fluorescent lipid. Insets show magnifications of the regions marked with squares.

**File Name:** Supplementary Movie 17

**Description:** Numerical simulation of the reshaping of three buds of different initial diameter (0.5, 1 and 1.5 μm) following protein binding from a protein bulk concentration of 0.35 μM.. Color is protein coverage (left) and order (right). Membrane tension is fixed to that prior to protein exposure and the volume enclosed between the membrane and the substrate is fixed.

**File Name:** Supplementary Movie 18

**Description:** Numerical simulation of the reshaping of three tubes of initial diameter approximately of 0.6 μm and different initial lengths (2, 3 and 5 μm) following protein binding from a protein bulk concentration of 0.35 μM. Color is protein coverage (left) and order (right). Membrane tension is fixed to that prior to protein exposure and the volume enclosed between the membrane and the substrate is fixed.

**File Name:** Supplementary Movie 19

**Description:** Time sequence of the reshaping of caps formed through pSLB destretch followed by a hypo-osmotic shock, before and after incubation with 1 μM Amphiphysin. The pSLB composition is DOPC:DOPS:DOPA 3:2:1, supplemented with 0.5 % mol LissRhod-DPPE. Images show the fluorescent lipid (left) and fluorescent Amphiphysin (right). Insets show magnifications of the regions marked with squares.

**File Name:** Supplementary Movie 20

**Description:** Time sequence of the reshaping of caps formed through pSLB destretch followed by a hypo-osmotic shock, before and after incubation of 3 μM Amphiphysin concentration at the first indicated injection, and 5 μM at the second one. The pSLB composition is DOPC:DOPS:DOPA 3:2:1, supplemented with 0.5 % mol LissRhod-DPPE. Images show the fluorescent lipid (left) and fluorescent Amphiphysin (right). Insets show magnifications of the regions marked with squares.

**File Name:** Supplementary Movie 21

**Description:** Time sequence of human dermal fibroblasts co-transfected with GFP-Mem (left) and mCherry-Amphiphysin (right) before, during and after stretching. Images show the fluorescent membrane (left) and Amphiphysin (right) markers (right). Insets show magnifications of the regions marked with squares.

**File Name:** Supplementary Software 1

**Description:** The program computes the chemo-mechanical response of lipid membrane exposed to N-BAR proteins using Matlab2016b
