# Supplementary material for: Dynamic mechanochemical feedback between curved membranes and BAR protein self-organization
Source: Nat Commun. 2021 Nov 12;12:6550. doi: 10.1038/s41467-021-26591-3 (PMC8589976; doi:10.1038/s41467-021-26591-3)
Supplement: Supplementary file 25 — Supplementary software 1 [file 41467_2021_26591_MOESM25_ESM.zip › Supplementary Software 1/Interpolation_Geometry/codegen/mex/evaluate_BSp/html/_coder_evaluate_BSp_api_c.html]

RTW Report - \_coder\_evaluate\_BSp\_api.c


|  |
| --- |
| File: \_coder\_evaluate\_BSp\_api.c  ```     1   /*     2    * Academic License - for use in teaching, academic research, and meeting     3    * course requirements at degree granting institutions only.  Not for     4    * government, commercial, or other organizational use.     5    *     6    * _coder_evaluate_BSp_api.c     7    *     8    * Code generation for function '_coder_evaluate_BSp_api'     9    *    10    */    11       12   /* Include files */    13   #include "rt_nonfinite.h"    14   #include "evaluate_BSp.h"    15   #include "_coder_evaluate_BSp_api.h"    16   #include "evaluate_BSp_emxutil.h"    17   #include "evaluate_BSp_data.h"    18       19   /* Variable Definitions */    20   static emlrtRTEInfo f_emlrtRTEI = { 1, /* lineNo */    21     1,                                   /* colNo */    22     "_coder_evaluate_BSp_api",           /* fName */    23     ""                                   /* pName */    24   };    25       26   /* Function Declarations */    27   static void b_emlrt_marshallIn(const emlrtStack *sp, const mxArray *u, const    28     emlrtMsgIdentifier *parentId, real_T **y_data, int32_T y_size[2]);    29   static const mxArray *b_emlrt_marshallOut(const real_T u);    30   static real_T c_emlrt_marshallIn(const emlrtStack *sp, const mxArray *p, const    31     char_T *identifier);    32   static real_T d_emlrt_marshallIn(const emlrtStack *sp, const mxArray *u, const    33     emlrtMsgIdentifier *parentId);    34   static void e_emlrt_marshallIn(const emlrtStack *sp, const mxArray *src, const    35     emlrtMsgIdentifier *msgId, real_T **ret_data, int32_T ret_size[2]);    36   static void emlrt_marshallIn(const emlrtStack *sp, const mxArray *U, const    37     char_T *identifier, real_T **y_data, int32_T y_size[2]);    38   static const mxArray *emlrt_marshallOut(const emxArray_real_T *u);    39   static real_T f_emlrt_marshallIn(const emlrtStack *sp, const mxArray *src, const    40     emlrtMsgIdentifier *msgId);    41       42   /* Function Definitions */    43   static void b_emlrt_marshallIn(const emlrtStack *sp, const mxArray *u, const    44     emlrtMsgIdentifier *parentId, real_T **y_data, int32_T y_size[2])    45   {    46     e_emlrt_marshallIn(sp, emlrtAlias(u), parentId, y_data, y_size);    47     emlrtDestroyArray(&u);    48   }    49       50   static const mxArray *b_emlrt_marshallOut(const real_T u)    51   {    52     const mxArray *y;    53     const mxArray *m1;    54     y = NULL;    55     m1 = emlrtCreateDoubleScalar(u);    56     emlrtAssign(&y, m1);    57     return y;    58   }    59       60   static real_T c_emlrt_marshallIn(const emlrtStack *sp, const mxArray *p, const    61     char_T *identifier)    62   {    63     real_T y;    64     emlrtMsgIdentifier thisId;    65     thisId.fIdentifier = identifier;    66     thisId.fParent = NULL;    67     thisId.bParentIsCell = false;    68     y = d_emlrt_marshallIn(sp, emlrtAlias(p), &thisId);    69     emlrtDestroyArray(&p);    70     return y;    71   }    72       73   static real_T d_emlrt_marshallIn(const emlrtStack *sp, const mxArray *u, const    74     emlrtMsgIdentifier *parentId)    75   {    76     real_T y;    77     y = f_emlrt_marshallIn(sp, emlrtAlias(u), parentId);    78     emlrtDestroyArray(&u);    79     return y;    80   }    81       82   static void e_emlrt_marshallIn(const emlrtStack *sp, const mxArray *src, const    83     emlrtMsgIdentifier *msgId, real_T **ret_data, int32_T ret_size[2])    84   {    85     static const int32_T dims[2] = { 1, 157 };    86       87     boolean_T bv0[2] = { false, true };    88       89     int32_T iv1[2];    90     emlrtCheckVsBuiltInR2012b(sp, msgId, src, "double", false, 2U, dims, &bv0[0],    91       iv1);    92     ret_size[0] = iv1[0];    93     ret_size[1] = iv1[1];    94     *ret_data = (real_T *)mxGetData(src);    95     emlrtDestroyArray(&src);    96   }    97       98   static void emlrt_marshallIn(const emlrtStack *sp, const mxArray *U, const    99     char_T *identifier, real_T **y_data, int32_T y_size[2])   100   {   101     emlrtMsgIdentifier thisId;   102     thisId.fIdentifier = identifier;   103     thisId.fParent = NULL;   104     thisId.bParentIsCell = false;   105     b_emlrt_marshallIn(sp, emlrtAlias(U), &thisId, y_data, y_size);   106     emlrtDestroyArray(&U);   107   }   108      109   static const mxArray *emlrt_marshallOut(const emxArray_real_T *u)   110   {   111     const mxArray *y;   112     const mxArray *m0;   113     static const int32_T iv0[2] = { 0, 0 };   114      115     y = NULL;   116     m0 = emlrtCreateNumericArray(2, iv0, mxDOUBLE_CLASS, mxREAL);   117     mxSetData((mxArray *)m0, (void *)&u->data[0]);   118     emlrtSetDimensions((mxArray *)m0, u->size, 2);   119     emlrtAssign(&y, m0);   120     return y;   121   }   122      123   static real_T f_emlrt_marshallIn(const emlrtStack *sp, const mxArray *src, const   124     emlrtMsgIdentifier *msgId)   125   {   126     real_T ret;   127     static const int32_T dims = 0;   128     emlrtCheckBuiltInR2012b(sp, msgId, src, "double", false, 0U, &dims);   129     ret = *(real_T *)mxGetData(src);   130     emlrtDestroyArray(&src);   131     return ret;   132   }   133      134   void evaluate_BSp_api(const mxArray * const prhs[4], const mxArray *plhs[2])   135   {   136     emxArray_real_T *ders;   137     real_T (*U_data)[157];   138     int32_T U_size[2];   139     real_T p;   140     real_T de;   141     real_T x;   142     real_T sp;   143     emlrtStack st = { NULL,              /* site */   144       NULL,                              /* tls */   145       NULL                               /* prev */   146     };   147      148     st.tls = emlrtRootTLSGlobal;   149     emlrtHeapReferenceStackEnterFcnR2012b(&st);   150     emxInit_real_T(&st, &ders, 2, &f_emlrtRTEI, true);   151      152     /* Marshall function inputs */   153     emlrt_marshallIn(&st, emlrtAlias(prhs[0]), "U", (real_T **)&U_data, U_size);   154     p = c_emlrt_marshallIn(&st, emlrtAliasP(prhs[1]), "p");   155     de = c_emlrt_marshallIn(&st, emlrtAliasP(prhs[2]), "de");   156     x = c_emlrt_marshallIn(&st, emlrtAliasP(prhs[3]), "x");   157      158     /* Invoke the target function */   159     evaluate_BSp(&st, *U_data, U_size, p, de, x, ders, &sp);   160      161     /* Marshall function outputs */   162     plhs[0] = emlrt_marshallOut(ders);   163     plhs[1] = b_emlrt_marshallOut(sp);   164     ders->canFreeData = false;   165     emxFree_real_T(&ders);   166     emlrtHeapReferenceStackLeaveFcnR2012b(&st);   167   }   168      169   /* End of code generation (_coder_evaluate_BSp_api.c) */   170 ``` |
