# Supplementary material for: Dynamic mechanochemical feedback between curved membranes and BAR protein self-organization
Source: Nat Commun. 2021 Nov 12;12:6550. doi: 10.1038/s41467-021-26591-3 (PMC8589976; doi:10.1038/s41467-021-26591-3)
Supplement: Supplementary file 25 — Supplementary software 1 [file 41467_2021_26591_MOESM25_ESM.zip › Supplementary Software 1/Interpolation_Geometry/codegen/mex/evaluate_BSp/html/resources/rtwmsg.html]

Block-to-Code Highlighting Message


# Block-to-Code Highlighting Message

For '%s', you cannot trace code from the model because there is no traceability information.

'%s' is a virtual block. Code is not generated for virtual blocks.

'%s' is reduced during model compilation. Code is not generated for reduced blocks.

Code for '%s' is from reusable function and cannot be traced.

'%s' is outside of source subsystem and cannot be traced.

'%s' contains illegal character (single quote) that disables model-to-code navigation. Remove single quote from block name and rebuild model.

'%s' is a masked subsystem and cannot be traced at the subsystem block level. Traceability information is available for individual blocks under the mask.
