# Supplementary material for: Dynamic mechanochemical feedback between curved membranes and BAR protein self-organization
Source: Nat Commun. 2021 Nov 12;12:6550. doi: 10.1038/s41467-021-26591-3 (PMC8589976; doi:10.1038/s41467-021-26591-3)
Supplement: Supplementary file 25 — Supplementary software 1 [file 41467_2021_26591_MOESM25_ESM.zip › Supplementary Software 1/Interpolation_Geometry/codegen/mex/evaluate_BSp/html/_coder_evaluate_BSp_info_c.html]

RTW Report - \_coder\_evaluate\_BSp\_info.c


|  |
| --- |
| File: \_coder\_evaluate\_BSp\_info.c  ```     1   /*     2    * Academic License - for use in teaching, academic research, and meeting     3    * course requirements at degree granting institutions only.  Not for     4    * government, commercial, or other organizational use.     5    *     6    * _coder_evaluate_BSp_info.c     7    *     8    * Code generation for function '_coder_evaluate_BSp_info'     9    *    10    */    11       12   /* Include files */    13   #include "rt_nonfinite.h"    14   #include "evaluate_BSp.h"    15   #include "_coder_evaluate_BSp_info.h"    16       17   /* Function Definitions */    18   mxArray *emlrtMexFcnProperties(void)    19   {    20     mxArray *xResult;    21     mxArray *xEntryPoints;    22     const char * fldNames[4] = { "Name", "NumberOfInputs", "NumberOfOutputs",    23       "ConstantInputs" };    24       25     mxArray *xInputs;    26     const char * b_fldNames[4] = { "Version", "ResolvedFunctions", "EntryPoints",    27       "CoverageInfo" };    28       29     xEntryPoints = emlrtCreateStructMatrix(1, 1, 4, fldNames);    30     xInputs = emlrtCreateLogicalMatrix(1, 4);    31     emlrtSetField(xEntryPoints, 0, "Name", mxCreateString("evaluate_BSp"));    32     emlrtSetField(xEntryPoints, 0, "NumberOfInputs", mxCreateDoubleScalar(4.0));    33     emlrtSetField(xEntryPoints, 0, "NumberOfOutputs", mxCreateDoubleScalar(2.0));    34     emlrtSetField(xEntryPoints, 0, "ConstantInputs", xInputs);    35     xResult = emlrtCreateStructMatrix(1, 1, 4, b_fldNames);    36     emlrtSetField(xResult, 0, "Version", mxCreateString("9.1.0.441655 (R2016b)"));    37     emlrtSetField(xResult, 0, "ResolvedFunctions", (mxArray *)    38                   emlrtMexFcnResolvedFunctionsInfo());    39     emlrtSetField(xResult, 0, "EntryPoints", xEntryPoints);    40     return xResult;    41   }    42       43   const mxArray *emlrtMexFcnResolvedFunctionsInfo(void)    44   {    45     const mxArray *nameCaptureInfo;    46     const char * data[12] = {    47       "789ced9acf6fe34414c75d54ca72d8058116c4811f7b435aa94e9ba69baeb4bb8d93a6493749f3b33fb24291634f92a9c71ec77652a7a7de40e2c06185800377"    48       "8e4820017f0407a4e53fe0ca9d2333f9d17a87a8f6da699a9246b29217e7cdfbbe97f974de4cca2da4b31c79dc21d73f2b1cb7449e6f91eb356ef0787d682f90",    49       "ebade1f3e0fd45eef6d0fe8a5c12d62c605b839b9aa8026ef490b10a3551b3ca3d1d70063031ea02b97fa7011128431564b0c3484162a849c7ad3383dea2afe3"    50       "2d2029a58eca192df32c0c879c463f9f2fb8f37c16c7e4b3e7c8e7eda1fd6ceb331e6143ee8a323078194b1636444d36790b9f9c403e014cc5c23a1fc732a8e5",    51       "8458914f93bc0d1d23d18258ab6d03ac02cbe8f1a02ba28e68819a50d297d5819e948b9ebb8c9ebbfdba121dcb9006d144b48c80d6b45ac3fc42178c377a38c7"    52       "1bd5c576d121307ed47e96ce1c90d260dde2b3b1722626f0c5d5d0ca7a9dd405a33ab679a0a2fe75bfaf98bf3f92cc0f24d31a8ce2471df197c6c45f70c47ff3",    53       "2c9fd377ff7efa67ccbf7fd0f893f3779b07ef712fd79fda525caaca8578215d3984ed84d5dda844844a7230dea78ef116c68cc7399efd7c7ed638cabbe8f990"    54       "d1436d8623ad8350cc34615353816671c1783a75d1b3c3f851db3f4f2f4b9f00572f7efa6d7eb97a50505abd5c37928c285b38b3bb9334570fc5d474b8fad645",    55       "afc8e8a5f6e4e6cd3dac01d168d6e8fb35d1c9c2b8ef639cbe5b8c3e6a4353c2a8a36a5efcdf60fca98d70134a22eac7d75dfc3719ff4daff541b0ceaba285c4"    56       "3a0f1079c18f542fab01e6e1af9fff30bf1c659231a503c3590944760ef605bb685a6b1b89e970340bf3c43b1f641c03dad78d8f81ea207c9cfe31cf7ce8e1b0",    57       "b18abaa8d0aa6aad43d39451b31d9991feadc4e8a5f604d719ada3d62ca303cef4b8f56f1f317aa84d42d548885a031b0863bd86bbc068207c5c93e8663058ff"    58       "e6569f32e357f65a1f274783725d90c3a0930bc0d737c207f3cb178edbed42235b8db50c9c30b632d6fe71b61a9f0e5f3fbae83d61f452fb32e6cfbd8b3f506b",    59       "01a403e35cf745bc2c0cdf3bd7bd48d6ae06f4f2fdbccfe44b6d66ff053519d864cbe7691d7bc88cf7f055ead7803690754c22f3547ff0fd12f7f53c73965f2d"    60       "874ef23dedc06e1f857b3ba9a372a95e9dd27ee9aae6c9a64bdcdb4c5c6a43932c7bc08092458f3dfdf126b544c3cb795d9c891ff79db743f3b000379cf8e364",    61       "3bacf44aed2644f1725c29b6856659a96c4d693f745dd723b7fdd012a39bda647855b483f1e5f677e53113f7b19f7a0df6517db5a3734c9ff372aefb3c64af57"    62       "41e86837548142a87c7cb222aea1fc94d69f79e30a6ad7892ba8dd70e59fab483aabe84a0ed8562a5b6ae6b79fe6b74348b83907bf39afb839afb80cdeaef2bc",    63       "c26dfe4cfbf75c3fe7e8aa21c32e9481dff54dc69d3a02def6959358a7b06ef223cdcb7a206e7ef9feaf2be5e6bb4971eb879be3f09ed1cbe52a92826331a359"    64       "ea0851797f4adcccc23cf1fa3bd17008dffddfabf0f188f17fe433ef51da2a17888fe74b85f95857c6fdbf58f4b8d0a81cacad3f4875517123118d667045e266",    65       "838fcb9a276efdd9c74c5c6ab3e7d1a6d081c84a6bb9c109d844b83975f1cf30fe19aff518dbd7b22904edcfb89feffcfe622e381ab7ce24105c11a3896672ad"    66       "bed189cabb9aba2e4ceb9c615639fa84894b6d8623531211b0f5385675d2f81112a6c15196f1cf7aadc7588efe9342c0f5887b3ecffb9cba7d982b364465af90",    67       "7d9adb2902ab142aedfecf3972abd33b4c5c6a331c9111cff3b8ecfeed09e3ffc46b1dc6f243a4934a04e8dfbe9c97fe6d1c2fd98a153a4cefc734ad528479b3"    68       "906b9ae5f6d6e5f3f22f3549e20d", "" };    69       70     nameCaptureInfo = NULL;    71     emlrtNameCaptureMxArrayR2016a(data, 12808U, &nameCaptureInfo);    72     return nameCaptureInfo;    73   }    74       75   /* End of code generation (_coder_evaluate_BSp_info.c) */    76 ``` |
