# Supplementary material for: Dynamic mechanochemical feedback between curved membranes and BAR protein self-organization
Source: Nat Commun. 2021 Nov 12;12:6550. doi: 10.1038/s41467-021-26591-3 (PMC8589976; doi:10.1038/s41467-021-26591-3)
Supplement: Supplementary file 25 — Supplementary software 1 [file 41467_2021_26591_MOESM25_ESM.zip › Supplementary Software 1/Interpolation_Geometry/codegen/mex/evaluate_BSp/html/_coder_evaluate_BSp_mex_c.html]

RTW Report - \_coder\_evaluate\_BSp\_mex.c


|  |
| --- |
| File: \_coder\_evaluate\_BSp\_mex.c  ```     1   /*     2    * Academic License - for use in teaching, academic research, and meeting     3    * course requirements at degree granting institutions only.  Not for     4    * government, commercial, or other organizational use.     5    *     6    * _coder_evaluate_BSp_mex.c     7    *     8    * Code generation for function '_coder_evaluate_BSp_mex'     9    *    10    */    11       12   /* Include files */    13   #include "evaluate_BSp.h"    14   #include "_coder_evaluate_BSp_mex.h"    15   #include "evaluate_BSp_terminate.h"    16   #include "_coder_evaluate_BSp_api.h"    17   #include "evaluate_BSp_initialize.h"    18   #include "evaluate_BSp_data.h"    19       20   /* Function Declarations */    21   static void evaluate_BSp_mexFunction(int32_T nlhs, mxArray *plhs[2], int32_T    22     nrhs, const mxArray *prhs[4]);    23       24   /* Function Definitions */    25   static void evaluate_BSp_mexFunction(int32_T nlhs, mxArray *plhs[2], int32_T    26     nrhs, const mxArray *prhs[4])    27   {    28     int32_T n;    29     const mxArray *inputs[4];    30     const mxArray *outputs[2];    31     int32_T b_nlhs;    32     emlrtStack st = { NULL,              /* site */    33       NULL,                              /* tls */    34       NULL                               /* prev */    35     };    36       37     st.tls = emlrtRootTLSGlobal;    38       39     /* Check for proper number of arguments. */    40     if (nrhs != 4) {    41       emlrtErrMsgIdAndTxt(&st, "EMLRT:runTime:WrongNumberOfInputs", 5, 12, 4, 4,    42                           12, "evaluate_BSp");    43     }    44       45     if (nlhs > 2) {    46       emlrtErrMsgIdAndTxt(&st, "EMLRT:runTime:TooManyOutputArguments", 3, 4, 12,    47                           "evaluate_BSp");    48     }    49       50     /* Temporary copy for mex inputs. */    51     for (n = 0; n < nrhs; n++) {    52       inputs[n] = prhs[n];    53       if (*emlrtBreakCheckR2012bFlagVar != 0) {    54         emlrtBreakCheckR2012b(&st);    55       }    56     }    57       58     /* Call the function. */    59     evaluate_BSp_api(inputs, outputs);    60       61     /* Copy over outputs to the caller. */    62     if (nlhs < 1) {    63       b_nlhs = 1;    64     } else {    65       b_nlhs = nlhs;    66     }    67       68     emlrtReturnArrays(b_nlhs, plhs, outputs);    69       70     /* Module termination. */    71     evaluate_BSp_terminate();    72   }    73       74   void mexFunction(int32_T nlhs, mxArray *plhs[], int32_T nrhs, const mxArray    75                    *prhs[])    76   {    77     mexAtExit(evaluate_BSp_atexit);    78       79     /* Initialize the memory manager. */    80     /* Module initialization. */    81     evaluate_BSp_initialize();    82       83     /* Dispatch the entry-point. */    84     evaluate_BSp_mexFunction(nlhs, plhs, nrhs, prhs);    85   }    86       87   emlrtCTX mexFunctionCreateRootTLS(void)    88   {    89     emlrtCreateRootTLS(&emlrtRootTLSGlobal, &emlrtContextGlobal, NULL, 1);    90     return emlrtRootTLSGlobal;    91   }    92       93   /* End of code generation (_coder_evaluate_BSp_mex.c) */    94 ``` |
