# Supplementary material for: Dynamic mechanochemical feedback between curved membranes and BAR protein self-organization
Source: Nat Commun. 2021 Nov 12;12:6550. doi: 10.1038/s41467-021-26591-3 (PMC8589976; doi:10.1038/s41467-021-26591-3)
Supplement: Supplementary file 25 — Supplementary software 1 [file 41467_2021_26591_MOESM25_ESM.zip › Supplementary Software 1/Interpolation_Geometry/codegen/mex/evaluate_BSp/html/evaluate_BSp_buildlog1.html]

Code Generation Report For 'evaluate\_BSp'


MATLAB Coder Build Log

|  |  |
| --- | --- |
| Build Parameters | |
| Build directory | /lordvader/doctorands/tozzi/Desktop/Code\_NBAR/Interpolation\_Geometry/codegen/mex/evaluate\_BSp |
| Make wrapper | sh evaluate\_BSp\_mex.sh |

  

|  |
| --- |
| Build Log |
| ```     1   /usr/bin/gcc -c -ansi -fexceptions -fPIC -fno-omit-frame-pointer -pthread -D_GNU_SOURCE -DMATLAB_MEX_FILE   -O -DNDEBUG    -I "/opt/MATLAB/R2016b/simulink/include" -I "/opt/MATLAB/R2016b/toolbox/shared/simtargets" -I "/lordvader/doctorands/tozzi/Desktop/Code_NBAR/Interpolation_Geometry" -I "/lordvader/doctorands/tozzi/Desktop/Code_NBAR/Interpolation_Geometry/codegen/mex/evaluate_BSp" -I "./interface" -I "/opt/MATLAB/R2016b/extern/include" -I "." "evaluate_BSp_data.c"     2   /usr/bin/gcc -c -ansi -fexceptions -fPIC -fno-omit-frame-pointer -pthread -D_GNU_SOURCE -DMATLAB_MEX_FILE   -O -DNDEBUG    -I "/opt/MATLAB/R2016b/simulink/include" -I "/opt/MATLAB/R2016b/toolbox/shared/simtargets" -I "/lordvader/doctorands/tozzi/Desktop/Code_NBAR/Interpolation_Geometry" -I "/lordvader/doctorands/tozzi/Desktop/Code_NBAR/Interpolation_Geometry/codegen/mex/evaluate_BSp" -I "./interface" -I "/opt/MATLAB/R2016b/extern/include" -I "." "evaluate_BSp_initialize.c"     3   /usr/bin/gcc -c -ansi -fexceptions -fPIC -fno-omit-frame-pointer -pthread -D_GNU_SOURCE -DMATLAB_MEX_FILE   -O -DNDEBUG    -I "/opt/MATLAB/R2016b/simulink/include" -I "/opt/MATLAB/R2016b/toolbox/shared/simtargets" -I "/lordvader/doctorands/tozzi/Desktop/Code_NBAR/Interpolation_Geometry" -I "/lordvader/doctorands/tozzi/Desktop/Code_NBAR/Interpolation_Geometry/codegen/mex/evaluate_BSp" -I "./interface" -I "/opt/MATLAB/R2016b/extern/include" -I "." "evaluate_BSp_terminate.c"     4   /usr/bin/gcc -c -ansi -fexceptions -fPIC -fno-omit-frame-pointer -pthread -D_GNU_SOURCE -DMATLAB_MEX_FILE   -O -DNDEBUG    -I "/opt/MATLAB/R2016b/simulink/include" -I "/opt/MATLAB/R2016b/toolbox/shared/simtargets" -I "/lordvader/doctorands/tozzi/Desktop/Code_NBAR/Interpolation_Geometry" -I "/lordvader/doctorands/tozzi/Desktop/Code_NBAR/Interpolation_Geometry/codegen/mex/evaluate_BSp" -I "./interface" -I "/opt/MATLAB/R2016b/extern/include" -I "." "evaluate_BSp.c"     5   /usr/bin/gcc -c -ansi -fexceptions -fPIC -fno-omit-frame-pointer -pthread -D_GNU_SOURCE -DMATLAB_MEX_FILE   -O -DNDEBUG    -I "/opt/MATLAB/R2016b/simulink/include" -I "/opt/MATLAB/R2016b/toolbox/shared/simtargets" -I "/lordvader/doctorands/tozzi/Desktop/Code_NBAR/Interpolation_Geometry" -I "/lordvader/doctorands/tozzi/Desktop/Code_NBAR/Interpolation_Geometry/codegen/mex/evaluate_BSp" -I "./interface" -I "/opt/MATLAB/R2016b/extern/include" -I "." "interface/_coder_evaluate_BSp_info.c"     6   /usr/bin/gcc -c -ansi -fexceptions -fPIC -fno-omit-frame-pointer -pthread -D_GNU_SOURCE -DMATLAB_MEX_FILE   -O -DNDEBUG    -I "/opt/MATLAB/R2016b/simulink/include" -I "/opt/MATLAB/R2016b/toolbox/shared/simtargets" -I "/lordvader/doctorands/tozzi/Desktop/Code_NBAR/Interpolation_Geometry" -I "/lordvader/doctorands/tozzi/Desktop/Code_NBAR/Interpolation_Geometry/codegen/mex/evaluate_BSp" -I "./interface" -I "/opt/MATLAB/R2016b/extern/include" -I "." "interface/_coder_evaluate_BSp_api.c"     7   /usr/bin/gcc -c -ansi -fexceptions -fPIC -fno-omit-frame-pointer -pthread -D_GNU_SOURCE -DMATLAB_MEX_FILE   -O -DNDEBUG    -I "/opt/MATLAB/R2016b/simulink/include" -I "/opt/MATLAB/R2016b/toolbox/shared/simtargets" -I "/lordvader/doctorands/tozzi/Desktop/Code_NBAR/Interpolation_Geometry" -I "/lordvader/doctorands/tozzi/Desktop/Code_NBAR/Interpolation_Geometry/codegen/mex/evaluate_BSp" -I "./interface" -I "/opt/MATLAB/R2016b/extern/include" -I "." "interface/_coder_evaluate_BSp_mex.c"     8   /usr/bin/gcc -c -ansi -fexceptions -fPIC -fno-omit-frame-pointer -pthread -D_GNU_SOURCE -DMATLAB_MEX_FILE   -O -DNDEBUG    -I "/opt/MATLAB/R2016b/simulink/include" -I "/opt/MATLAB/R2016b/toolbox/shared/simtargets" -I "/lordvader/doctorands/tozzi/Desktop/Code_NBAR/Interpolation_Geometry" -I "/lordvader/doctorands/tozzi/Desktop/Code_NBAR/Interpolation_Geometry/codegen/mex/evaluate_BSp" -I "./interface" -I "/opt/MATLAB/R2016b/extern/include" -I "." "evaluate_BSp_emxutil.c"     9   /usr/bin/gcc -Wl,--version-script,evaluate_BSp_mex_mex.map evaluate_BSp_data.o evaluate_BSp_initialize.o evaluate_BSp_terminate.o evaluate_BSp.o _coder_evaluate_BSp_info.o _coder_evaluate_BSp_api.o _coder_evaluate_BSp_mex.o evaluate_BSp_emxutil.o -pthread -Wl,--no-undefined -Wl,-rpath-link,/opt/MATLAB/R2016b/bin/glnxa64 -shared -L/opt/MATLAB/R2016b/bin/glnxa64 -lmx -lmex -lmat -lm -lstdc++    -o evaluate_BSp_mex.mexa64    -lemlrt -lcovrt -lut -lmwmathutil ``` |
