# Supplementary material for: Dynamic mechanochemical feedback between curved membranes and BAR protein self-organization
Source: Nat Commun. 2021 Nov 12;12:6550. doi: 10.1038/s41467-021-26591-3 (PMC8589976; doi:10.1038/s41467-021-26591-3)
Supplement: Supplementary file 25 — Supplementary software 1 [file 41467_2021_26591_MOESM25_ESM.zip › Supplementary Software 1/Interpolation_Geometry/codegen/mex/evaluate_BSp/html/evaluate_BSp_buildlog2.html]

Code Generation Report For 'evaluate\_BSp'


MATLAB Coder Build Log

|  |  |
| --- | --- |
| Build Parameters | |
| Build directory | /lordvader/doctorands/tozzi/Desktop/Code\_NBAR/Interpolation\_Geometry/codegen/mex/evaluate\_BSp |

  

|  |
| --- |
| Build Log |
|  |
