# Supplementary material for: Dynamic mechanochemical feedback between curved membranes and BAR protein self-organization
Source: Nat Commun. 2021 Nov 12;12:6550. doi: 10.1038/s41467-021-26591-3 (PMC8589976; doi:10.1038/s41467-021-26591-3)
Supplement: Supplementary file 25 — Supplementary software 1 [file 41467_2021_26591_MOESM25_ESM.zip › Supplementary Software 1/Interpolation_Geometry/codegen/mex/evaluate_BSp/html/evaluate_BSp_c.html]

RTW Report - evaluate\_BSp.c


|  |
| --- |
| File: evaluate\_BSp.c  ```     1   /*     2    * Academic License - for use in teaching, academic research, and meeting     3    * course requirements at degree granting institutions only.  Not for     4    * government, commercial, or other organizational use.     5    *     6    * evaluate_BSp.c     7    *     8    * Code generation for function 'evaluate_BSp'     9    *    10    */    11       12   /* Include files */    13   #include "rt_nonfinite.h"    14   #include "evaluate_BSp.h"    15   #include "evaluate_BSp_emxutil.h"    16   #include "evaluate_BSp_data.h"    17       18   /* Variable Definitions */    19   static emlrtRSInfo emlrtRSI = { 16,    /* lineNo */    20     "evaluate_BSp",                      /* fcnName */    21     "/lordvader/doctorands/tozzi/Desktop/Code_NBAR/Interpolation_Geometry/evaluate_BSp.m"/* pathName */    22   };    23       24   static emlrtRSInfo b_emlrtRSI = { 12,  /* lineNo */    25     "nullAssignment",                    /* fcnName */    26     "/opt/MATLAB/R2016b/toolbox/eml/eml/+coder/+internal/nullAssignment.m"/* pathName */    27   };    28       29   static emlrtRSInfo c_emlrtRSI = { 16,  /* lineNo */    30     "nullAssignment",                    /* fcnName */    31     "/opt/MATLAB/R2016b/toolbox/eml/eml/+coder/+internal/nullAssignment.m"/* pathName */    32   };    33       34   static emlrtRTEInfo emlrtRTEI = { 1,   /* lineNo */    35     20,                                  /* colNo */    36     "evaluate_BSp",                      /* fName */    37     "/lordvader/doctorands/tozzi/Desktop/Code_NBAR/Interpolation_Geometry/evaluate_BSp.m"/* pName */    38   };    39       40   static emlrtRTEInfo b_emlrtRTEI = { 21,/* lineNo */    41     1,                                   /* colNo */    42     "evaluate_BSp",                      /* fName */    43     "/lordvader/doctorands/tozzi/Desktop/Code_NBAR/Interpolation_Geometry/evaluate_BSp.m"/* pName */    44   };    45       46   static emlrtRTEInfo c_emlrtRTEI = { 22,/* lineNo */    47     1,                                   /* colNo */    48     "evaluate_BSp",                      /* fName */    49     "/lordvader/doctorands/tozzi/Desktop/Code_NBAR/Interpolation_Geometry/evaluate_BSp.m"/* pName */    50   };    51       52   static emlrtRTEInfo d_emlrtRTEI = { 23,/* lineNo */    53     1,                                   /* colNo */    54     "evaluate_BSp",                      /* fName */    55     "/lordvader/doctorands/tozzi/Desktop/Code_NBAR/Interpolation_Geometry/evaluate_BSp.m"/* pName */    56   };    57       58   static emlrtRTEInfo e_emlrtRTEI = { 27,/* lineNo */    59     1,                                   /* colNo */    60     "evaluate_BSp",                      /* fName */    61     "/lordvader/doctorands/tozzi/Desktop/Code_NBAR/Interpolation_Geometry/evaluate_BSp.m"/* pName */    62   };    63       64   static emlrtRTEInfo g_emlrtRTEI = { 29,/* lineNo */    65     9,                                   /* colNo */    66     "evaluate_BSp",                      /* fName */    67     "/lordvader/doctorands/tozzi/Desktop/Code_NBAR/Interpolation_Geometry/evaluate_BSp.m"/* pName */    68   };    69       70   static emlrtBCInfo emlrtBCI = { -1,    /* iFirst */    71     -1,                                  /* iLast */    72     24,                                  /* lineNo */    73     8,                                   /* colNo */    74     "Nshape",                            /* aName */    75     "evaluate_BSp",                      /* fName */    76     "/lordvader/doctorands/tozzi/Desktop/Code_NBAR/Interpolation_Geometry/evaluate_BSp.m",/* pName */    77     0                                    /* checkKind */    78   };    79       80   static emlrtBCInfo b_emlrtBCI = { -1,  /* iFirst */    81     -1,                                  /* iLast */    82     24,                                  /* lineNo */    83     10,                                  /* colNo */    84     "Nshape",                            /* aName */    85     "evaluate_BSp",                      /* fName */    86     "/lordvader/doctorands/tozzi/Desktop/Code_NBAR/Interpolation_Geometry/evaluate_BSp.m",/* pName */    87     0                                    /* checkKind */    88   };    89       90   static emlrtBCInfo c_emlrtBCI = { -1,  /* iFirst */    91     -1,                                  /* iLast */    92     37,                                  /* lineNo */    93     23,                                  /* colNo */    94     "Nshape",                            /* aName */    95     "evaluate_BSp",                      /* fName */    96     "/lordvader/doctorands/tozzi/Desktop/Code_NBAR/Interpolation_Geometry/evaluate_BSp.m",/* pName */    97     0                                    /* checkKind */    98   };    99      100   static emlrtBCInfo d_emlrtBCI = { -1,  /* iFirst */   101     -1,                                  /* iLast */   102     37,                                  /* lineNo */   103     26,                                  /* colNo */   104     "Nshape",                            /* aName */   105     "evaluate_BSp",                      /* fName */   106     "/lordvader/doctorands/tozzi/Desktop/Code_NBAR/Interpolation_Geometry/evaluate_BSp.m",/* pName */   107     0                                    /* checkKind */   108   };   109      110   static emlrtBCInfo e_emlrtBCI = { -1,  /* iFirst */   111     -1,                                  /* iLast */   112     37,                                  /* lineNo */   113     36,                                  /* colNo */   114     "Nshape",                            /* aName */   115     "evaluate_BSp",                      /* fName */   116     "/lordvader/doctorands/tozzi/Desktop/Code_NBAR/Interpolation_Geometry/evaluate_BSp.m",/* pName */   117     0                                    /* checkKind */   118   };   119      120   static emlrtBCInfo f_emlrtBCI = { -1,  /* iFirst */   121     -1,                                  /* iLast */   122     37,                                  /* lineNo */   123     40,                                  /* colNo */   124     "Nshape",                            /* aName */   125     "evaluate_BSp",                      /* fName */   126     "/lordvader/doctorands/tozzi/Desktop/Code_NBAR/Interpolation_Geometry/evaluate_BSp.m",/* pName */   127     0                                    /* checkKind */   128   };   129      130   static emlrtRTEInfo h_emlrtRTEI = { 46,/* lineNo */   131     9,                                   /* colNo */   132     "evaluate_BSp",                      /* fName */   133     "/lordvader/doctorands/tozzi/Desktop/Code_NBAR/Interpolation_Geometry/evaluate_BSp.m"/* pName */   134   };   135      136   static emlrtRTEInfo i_emlrtRTEI = { 50,/* lineNo */   137     9,                                   /* colNo */   138     "evaluate_BSp",                      /* fName */   139     "/lordvader/doctorands/tozzi/Desktop/Code_NBAR/Interpolation_Geometry/evaluate_BSp.m"/* pName */   140   };   141      142   static emlrtRTEInfo j_emlrtRTEI = { 55,/* lineNo */   143     13,                                  /* colNo */   144     "evaluate_BSp",                      /* fName */   145     "/lordvader/doctorands/tozzi/Desktop/Code_NBAR/Interpolation_Geometry/evaluate_BSp.m"/* pName */   146   };   147      148   static emlrtBCInfo g_emlrtBCI = { -1,  /* iFirst */   149     -1,                                  /* iLast */   150     54,                                  /* lineNo */   151     9,                                   /* colNo */   152     "a",                                 /* aName */   153     "evaluate_BSp",                      /* fName */   154     "/lordvader/doctorands/tozzi/Desktop/Code_NBAR/Interpolation_Geometry/evaluate_BSp.m",/* pName */   155     0                                    /* checkKind */   156   };   157      158   static emlrtBCInfo h_emlrtBCI = { -1,  /* iFirst */   159     -1,                                  /* iLast */   160     63,                                  /* lineNo */   161     28,                                  /* colNo */   162     "a",                                 /* aName */   163     "evaluate_BSp",                      /* fName */   164     "/lordvader/doctorands/tozzi/Desktop/Code_NBAR/Interpolation_Geometry/evaluate_BSp.m",/* pName */   165     0                                    /* checkKind */   166   };   167      168   static emlrtBCInfo i_emlrtBCI = { -1,  /* iFirst */   169     -1,                                  /* iLast */   170     63,                                  /* lineNo */   171     38,                                  /* colNo */   172     "Nshape",                            /* aName */   173     "evaluate_BSp",                      /* fName */   174     "/lordvader/doctorands/tozzi/Desktop/Code_NBAR/Interpolation_Geometry/evaluate_BSp.m",/* pName */   175     0                                    /* checkKind */   176   };   177      178   static emlrtBCInfo j_emlrtBCI = { -1,  /* iFirst */   179     -1,                                  /* iLast */   180     63,                                  /* lineNo */   181     44,                                  /* colNo */   182     "Nshape",                            /* aName */   183     "evaluate_BSp",                      /* fName */   184     "/lordvader/doctorands/tozzi/Desktop/Code_NBAR/Interpolation_Geometry/evaluate_BSp.m",/* pName */   185     0                                    /* checkKind */   186   };   187      188   static emlrtBCInfo k_emlrtBCI = { -1,  /* iFirst */   189     -1,                                  /* iLast */   190     63,                                  /* lineNo */   191     18,                                  /* colNo */   192     "a",                                 /* aName */   193     "evaluate_BSp",                      /* fName */   194     "/lordvader/doctorands/tozzi/Desktop/Code_NBAR/Interpolation_Geometry/evaluate_BSp.m",/* pName */   195     0                                    /* checkKind */   196   };   197      198   static emlrtBCInfo l_emlrtBCI = { -1,  /* iFirst */   199     -1,                                  /* iLast */   200     64,                                  /* lineNo */   201     22,                                  /* colNo */   202     "a",                                 /* aName */   203     "evaluate_BSp",                      /* fName */   204     "/lordvader/doctorands/tozzi/Desktop/Code_NBAR/Interpolation_Geometry/evaluate_BSp.m",/* pName */   205     0                                    /* checkKind */   206   };   207      208   static emlrtRTEInfo k_emlrtRTEI = { 76,/* lineNo */   209     17,                                  /* colNo */   210     "evaluate_BSp",                      /* fName */   211     "/lordvader/doctorands/tozzi/Desktop/Code_NBAR/Interpolation_Geometry/evaluate_BSp.m"/* pName */   212   };   213      214   static emlrtBCInfo m_emlrtBCI = { -1,  /* iFirst */   215     -1,                                  /* iLast */   216     78,                                  /* lineNo */   217     30,                                  /* colNo */   218     "a",                                 /* aName */   219     "evaluate_BSp",                      /* fName */   220     "/lordvader/doctorands/tozzi/Desktop/Code_NBAR/Interpolation_Geometry/evaluate_BSp.m",/* pName */   221     0                                    /* checkKind */   222   };   223      224   static emlrtBCInfo n_emlrtBCI = { -1,  /* iFirst */   225     -1,                                  /* iLast */   226     78,                                  /* lineNo */   227     39,                                  /* colNo */   228     "a",                                 /* aName */   229     "evaluate_BSp",                      /* fName */   230     "/lordvader/doctorands/tozzi/Desktop/Code_NBAR/Interpolation_Geometry/evaluate_BSp.m",/* pName */   231     0                                    /* checkKind */   232   };   233      234   static emlrtBCInfo o_emlrtBCI = { -1,  /* iFirst */   235     -1,                                  /* iLast */   236     82,                                  /* lineNo */   237     30,                                  /* colNo */   238     "a",                                 /* aName */   239     "evaluate_BSp",                      /* fName */   240     "/lordvader/doctorands/tozzi/Desktop/Code_NBAR/Interpolation_Geometry/evaluate_BSp.m",/* pName */   241     0                                    /* checkKind */   242   };   243      244   static emlrtBCInfo p_emlrtBCI = { -1,  /* iFirst */   245     -1,                                  /* iLast */   246     82,                                  /* lineNo */   247     43,                                  /* colNo */   248     "Nshape",                            /* aName */   249     "evaluate_BSp",                      /* fName */   250     "/lordvader/doctorands/tozzi/Desktop/Code_NBAR/Interpolation_Geometry/evaluate_BSp.m",/* pName */   251     0                                    /* checkKind */   252   };   253      254   static emlrtBCInfo q_emlrtBCI = { -1,  /* iFirst */   255     -1,                                  /* iLast */   256     82,                                  /* lineNo */   257     49,                                  /* colNo */   258     "Nshape",                            /* aName */   259     "evaluate_BSp",                      /* fName */   260     "/lordvader/doctorands/tozzi/Desktop/Code_NBAR/Interpolation_Geometry/evaluate_BSp.m",/* pName */   261     0                                    /* checkKind */   262   };   263      264   static emlrtRTEInfo l_emlrtRTEI = { 94,/* lineNo */   265     10,                                  /* colNo */   266     "evaluate_BSp",                      /* fName */   267     "/lordvader/doctorands/tozzi/Desktop/Code_NBAR/Interpolation_Geometry/evaluate_BSp.m"/* pName */   268   };   269      270   static emlrtRTEInfo m_emlrtRTEI = { 96,/* lineNo */   271     14,                                  /* colNo */   272     "evaluate_BSp",                      /* fName */   273     "/lordvader/doctorands/tozzi/Desktop/Code_NBAR/Interpolation_Geometry/evaluate_BSp.m"/* pName */   274   };   275      276   static emlrtBCInfo r_emlrtBCI = { -1,  /* iFirst */   277     -1,                                  /* iLast */   278     48,                                  /* lineNo */   279     10,                                  /* colNo */   280     "ders",                              /* aName */   281     "evaluate_BSp",                      /* fName */   282     "/lordvader/doctorands/tozzi/Desktop/Code_NBAR/Interpolation_Geometry/evaluate_BSp.m",/* pName */   283     0                                    /* checkKind */   284   };   285      286   static emlrtRTEInfo n_emlrtRTEI = { 83,/* lineNo */   287     27,                                  /* colNo */   288     "nullAssignment",                    /* fName */   289     "/opt/MATLAB/R2016b/toolbox/eml/eml/+coder/+internal/nullAssignment.m"/* pName */   290   };   291      292   static emlrtRTEInfo o_emlrtRTEI = { 182,/* lineNo */   293     9,                                   /* colNo */   294     "nullAssignment",                    /* fName */   295     "/opt/MATLAB/R2016b/toolbox/eml/eml/+coder/+internal/nullAssignment.m"/* pName */   296   };   297      298   static emlrtDCInfo emlrtDCI = { 21,    /* lineNo */   299     16,                                  /* colNo */   300     "evaluate_BSp",                      /* fName */   301     "/lordvader/doctorands/tozzi/Desktop/Code_NBAR/Interpolation_Geometry/evaluate_BSp.m",/* pName */   302     1                                    /* checkKind */   303   };   304      305   static emlrtDCInfo b_emlrtDCI = { 21,  /* lineNo */   306     16,                                  /* colNo */   307     "evaluate_BSp",                      /* fName */   308     "/lordvader/doctorands/tozzi/Desktop/Code_NBAR/Interpolation_Geometry/evaluate_BSp.m",/* pName */   309     4                                    /* checkKind */   310   };   311      312   static emlrtDCInfo c_emlrtDCI = { 21,  /* lineNo */   313     19,                                  /* colNo */   314     "evaluate_BSp",                      /* fName */   315     "/lordvader/doctorands/tozzi/Desktop/Code_NBAR/Interpolation_Geometry/evaluate_BSp.m",/* pName */   316     1                                    /* checkKind */   317   };   318      319   static emlrtDCInfo d_emlrtDCI = { 21,  /* lineNo */   320     19,                                  /* colNo */   321     "evaluate_BSp",                      /* fName */   322     "/lordvader/doctorands/tozzi/Desktop/Code_NBAR/Interpolation_Geometry/evaluate_BSp.m",/* pName */   323     4                                    /* checkKind */   324   };   325      326   static emlrtDCInfo e_emlrtDCI = { 22,  /* lineNo */   327     16,                                  /* colNo */   328     "evaluate_BSp",                      /* fName */   329     "/lordvader/doctorands/tozzi/Desktop/Code_NBAR/Interpolation_Geometry/evaluate_BSp.m",/* pName */   330     1                                    /* checkKind */   331   };   332      333   static emlrtDCInfo f_emlrtDCI = { 22,  /* lineNo */   334     16,                                  /* colNo */   335     "evaluate_BSp",                      /* fName */   336     "/lordvader/doctorands/tozzi/Desktop/Code_NBAR/Interpolation_Geometry/evaluate_BSp.m",/* pName */   337     4                                    /* checkKind */   338   };   339      340   static emlrtDCInfo g_emlrtDCI = { 23,  /* lineNo */   341     17,                                  /* colNo */   342     "evaluate_BSp",                      /* fName */   343     "/lordvader/doctorands/tozzi/Desktop/Code_NBAR/Interpolation_Geometry/evaluate_BSp.m",/* pName */   344     1                                    /* checkKind */   345   };   346      347   static emlrtDCInfo h_emlrtDCI = { 26,  /* lineNo */   348     13,                                  /* colNo */   349     "evaluate_BSp",                      /* fName */   350     "/lordvader/doctorands/tozzi/Desktop/Code_NBAR/Interpolation_Geometry/evaluate_BSp.m",/* pName */   351     1                                    /* checkKind */   352   };   353      354   static emlrtDCInfo i_emlrtDCI = { 26,  /* lineNo */   355     13,                                  /* colNo */   356     "evaluate_BSp",                      /* fName */   357     "/lordvader/doctorands/tozzi/Desktop/Code_NBAR/Interpolation_Geometry/evaluate_BSp.m",/* pName */   358     4                                    /* checkKind */   359   };   360      361   static emlrtDCInfo j_emlrtDCI = { 26,  /* lineNo */   362     18,                                  /* colNo */   363     "evaluate_BSp",                      /* fName */   364     "/lordvader/doctorands/tozzi/Desktop/Code_NBAR/Interpolation_Geometry/evaluate_BSp.m",/* pName */   365     1                                    /* checkKind */   366   };   367      368   static emlrtDCInfo k_emlrtDCI = { 27,  /* lineNo */   369     13,                                  /* colNo */   370     "evaluate_BSp",                      /* fName */   371     "/lordvader/doctorands/tozzi/Desktop/Code_NBAR/Interpolation_Geometry/evaluate_BSp.m",/* pName */   372     1                                    /* checkKind */   373   };   374      375   static emlrtDCInfo l_emlrtDCI = { 27,  /* lineNo */   376     13,                                  /* colNo */   377     "evaluate_BSp",                      /* fName */   378     "/lordvader/doctorands/tozzi/Desktop/Code_NBAR/Interpolation_Geometry/evaluate_BSp.m",/* pName */   379     4                                    /* checkKind */   380   };   381      382   static emlrtBCInfo s_emlrtBCI = { -1,  /* iFirst */   383     -1,                                  /* iLast */   384     12,                                  /* lineNo */   385     7,                                   /* colNo */   386     "U",                                 /* aName */   387     "evaluate_BSp",                      /* fName */   388     "/lordvader/doctorands/tozzi/Desktop/Code_NBAR/Interpolation_Geometry/evaluate_BSp.m",/* pName */   389     0                                    /* checkKind */   390   };   391      392   static emlrtDCInfo m_emlrtDCI = { 12,  /* lineNo */   393     7,                                   /* colNo */   394     "evaluate_BSp",                      /* fName */   395     "/lordvader/doctorands/tozzi/Desktop/Code_NBAR/Interpolation_Geometry/evaluate_BSp.m",/* pName */   396     1                                    /* checkKind */   397   };   398      399   static emlrtBCInfo t_emlrtBCI = { -1,  /* iFirst */   400     -1,                                  /* iLast */   401     12,                                  /* lineNo */   402     15,                                  /* colNo */   403     "U",                                 /* aName */   404     "evaluate_BSp",                      /* fName */   405     "/lordvader/doctorands/tozzi/Desktop/Code_NBAR/Interpolation_Geometry/evaluate_BSp.m",/* pName */   406     0                                    /* checkKind */   407   };   408      409   static emlrtDCInfo n_emlrtDCI = { 12,  /* lineNo */   410     15,                                  /* colNo */   411     "evaluate_BSp",                      /* fName */   412     "/lordvader/doctorands/tozzi/Desktop/Code_NBAR/Interpolation_Geometry/evaluate_BSp.m",/* pName */   413     1                                    /* checkKind */   414   };   415      416   static emlrtBCInfo u_emlrtBCI = { -1,  /* iFirst */   417     -1,                                  /* iLast */   418     98,                                  /* lineNo */   419     29,                                  /* colNo */   420     "ders",                              /* aName */   421     "evaluate_BSp",                      /* fName */   422     "/lordvader/doctorands/tozzi/Desktop/Code_NBAR/Interpolation_Geometry/evaluate_BSp.m",/* pName */   423     0                                    /* checkKind */   424   };   425      426   static emlrtBCInfo v_emlrtBCI = { -1,  /* iFirst */   427     -1,                                  /* iLast */   428     98,                                  /* lineNo */   429     33,                                  /* colNo */   430     "ders",                              /* aName */   431     "evaluate_BSp",                      /* fName */   432     "/lordvader/doctorands/tozzi/Desktop/Code_NBAR/Interpolation_Geometry/evaluate_BSp.m",/* pName */   433     0                                    /* checkKind */   434   };   435      436   static emlrtBCInfo w_emlrtBCI = { -1,  /* iFirst */   437     -1,                                  /* iLast */   438     98,                                  /* lineNo */   439     14,                                  /* colNo */   440     "ders",                              /* aName */   441     "evaluate_BSp",                      /* fName */   442     "/lordvader/doctorands/tozzi/Desktop/Code_NBAR/Interpolation_Geometry/evaluate_BSp.m",/* pName */   443     0                                    /* checkKind */   444   };   445      446   static emlrtBCInfo x_emlrtBCI = { -1,  /* iFirst */   447     -1,                                  /* iLast */   448     98,                                  /* lineNo */   449     18,                                  /* colNo */   450     "ders",                              /* aName */   451     "evaluate_BSp",                      /* fName */   452     "/lordvader/doctorands/tozzi/Desktop/Code_NBAR/Interpolation_Geometry/evaluate_BSp.m",/* pName */   453     0                                    /* checkKind */   454   };   455      456   static emlrtBCInfo y_emlrtBCI = { -1,  /* iFirst */   457     -1,                                  /* iLast */   458     64,                                  /* lineNo */   459     32,                                  /* colNo */   460     "Nshape",                            /* aName */   461     "evaluate_BSp",                      /* fName */   462     "/lordvader/doctorands/tozzi/Desktop/Code_NBAR/Interpolation_Geometry/evaluate_BSp.m",/* pName */   463     0                                    /* checkKind */   464   };   465      466   static emlrtBCInfo ab_emlrtBCI = { -1, /* iFirst */   467     -1,                                  /* iLast */   468     64,                                  /* lineNo */   469     36,                                  /* colNo */   470     "Nshape",                            /* aName */   471     "evaluate_BSp",                      /* fName */   472     "/lordvader/doctorands/tozzi/Desktop/Code_NBAR/Interpolation_Geometry/evaluate_BSp.m",/* pName */   473     0                                    /* checkKind */   474   };   475      476   static emlrtBCInfo bb_emlrtBCI = { -1, /* iFirst */   477     -1,                                  /* iLast */   478     82,                                  /* lineNo */   479     18,                                  /* colNo */   480     "a",                                 /* aName */   481     "evaluate_BSp",                      /* fName */   482     "/lordvader/doctorands/tozzi/Desktop/Code_NBAR/Interpolation_Geometry/evaluate_BSp.m",/* pName */   483     0                                    /* checkKind */   484   };   485      486   static emlrtBCInfo cb_emlrtBCI = { -1, /* iFirst */   487     -1,                                  /* iLast */   488     83,                                  /* lineNo */   489     24,                                  /* colNo */   490     "a",                                 /* aName */   491     "evaluate_BSp",                      /* fName */   492     "/lordvader/doctorands/tozzi/Desktop/Code_NBAR/Interpolation_Geometry/evaluate_BSp.m",/* pName */   493     0                                    /* checkKind */   494   };   495      496   static emlrtBCInfo db_emlrtBCI = { -1, /* iFirst */   497     -1,                                  /* iLast */   498     83,                                  /* lineNo */   499     35,                                  /* colNo */   500     "Nshape",                            /* aName */   501     "evaluate_BSp",                      /* fName */   502     "/lordvader/doctorands/tozzi/Desktop/Code_NBAR/Interpolation_Geometry/evaluate_BSp.m",/* pName */   503     0                                    /* checkKind */   504   };   505      506   static emlrtBCInfo eb_emlrtBCI = { -1, /* iFirst */   507     -1,                                  /* iLast */   508     83,                                  /* lineNo */   509     38,                                  /* colNo */   510     "Nshape",                            /* aName */   511     "evaluate_BSp",                      /* fName */   512     "/lordvader/doctorands/tozzi/Desktop/Code_NBAR/Interpolation_Geometry/evaluate_BSp.m",/* pName */   513     0                                    /* checkKind */   514   };   515      516   static emlrtBCInfo fb_emlrtBCI = { -1, /* iFirst */   517     -1,                                  /* iLast */   518     85,                                  /* lineNo */   519     14,                                  /* colNo */   520     "ders",                              /* aName */   521     "evaluate_BSp",                      /* fName */   522     "/lordvader/doctorands/tozzi/Desktop/Code_NBAR/Interpolation_Geometry/evaluate_BSp.m",/* pName */   523     0                                    /* checkKind */   524   };   525      526   static emlrtBCInfo gb_emlrtBCI = { -1, /* iFirst */   527     -1,                                  /* iLast */   528     85,                                  /* lineNo */   529     17,                                  /* colNo */   530     "ders",                              /* aName */   531     "evaluate_BSp",                      /* fName */   532     "/lordvader/doctorands/tozzi/Desktop/Code_NBAR/Interpolation_Geometry/evaluate_BSp.m",/* pName */   533     0                                    /* checkKind */   534   };   535      536   static emlrtBCInfo hb_emlrtBCI = { -1, /* iFirst */   537     -1,                                  /* iLast */   538     78,                                  /* lineNo */   539     53,                                  /* colNo */   540     "Nshape",                            /* aName */   541     "evaluate_BSp",                      /* fName */   542     "/lordvader/doctorands/tozzi/Desktop/Code_NBAR/Interpolation_Geometry/evaluate_BSp.m",/* pName */   543     0                                    /* checkKind */   544   };   545      546   static emlrtBCInfo ib_emlrtBCI = { -1, /* iFirst */   547     -1,                                  /* iLast */   548     78,                                  /* lineNo */   549     59,                                  /* colNo */   550     "Nshape",                            /* aName */   551     "evaluate_BSp",                      /* fName */   552     "/lordvader/doctorands/tozzi/Desktop/Code_NBAR/Interpolation_Geometry/evaluate_BSp.m",/* pName */   553     0                                    /* checkKind */   554   };   555      556   static emlrtBCInfo jb_emlrtBCI = { -1, /* iFirst */   557     -1,                                  /* iLast */   558     78,                                  /* lineNo */   559     18,                                  /* colNo */   560     "a",                                 /* aName */   561     "evaluate_BSp",                      /* fName */   562     "/lordvader/doctorands/tozzi/Desktop/Code_NBAR/Interpolation_Geometry/evaluate_BSp.m",/* pName */   563     0                                    /* checkKind */   564   };   565      566   static emlrtBCInfo kb_emlrtBCI = { -1, /* iFirst */   567     -1,                                  /* iLast */   568     79,                                  /* lineNo */   569     24,                                  /* colNo */   570     "a",                                 /* aName */   571     "evaluate_BSp",                      /* fName */   572     "/lordvader/doctorands/tozzi/Desktop/Code_NBAR/Interpolation_Geometry/evaluate_BSp.m",/* pName */   573     0                                    /* checkKind */   574   };   575      576   static emlrtBCInfo lb_emlrtBCI = { -1, /* iFirst */   577     -1,                                  /* iLast */   578     79,                                  /* lineNo */   579     35,                                  /* colNo */   580     "Nshape",                            /* aName */   581     "evaluate_BSp",                      /* fName */   582     "/lordvader/doctorands/tozzi/Desktop/Code_NBAR/Interpolation_Geometry/evaluate_BSp.m",/* pName */   583     0                                    /* checkKind */   584   };   585      586   static emlrtBCInfo mb_emlrtBCI = { -1, /* iFirst */   587     -1,                                  /* iLast */   588     79,                                  /* lineNo */   589     41,                                  /* colNo */   590     "Nshape",                            /* aName */   591     "evaluate_BSp",                      /* fName */   592     "/lordvader/doctorands/tozzi/Desktop/Code_NBAR/Interpolation_Geometry/evaluate_BSp.m",/* pName */   593     0                                    /* checkKind */   594   };   595      596   static emlrtBCInfo nb_emlrtBCI = { -1, /* iFirst */   597     -1,                                  /* iLast */   598     48,                                  /* lineNo */   599     25,                                  /* colNo */   600     "Nshape",                            /* aName */   601     "evaluate_BSp",                      /* fName */   602     "/lordvader/doctorands/tozzi/Desktop/Code_NBAR/Interpolation_Geometry/evaluate_BSp.m",/* pName */   603     0                                    /* checkKind */   604   };   605      606   static emlrtBCInfo ob_emlrtBCI = { -1, /* iFirst */   607     -1,                                  /* iLast */   608     48,                                  /* lineNo */   609     28,                                  /* colNo */   610     "Nshape",                            /* aName */   611     "evaluate_BSp",                      /* fName */   612     "/lordvader/doctorands/tozzi/Desktop/Code_NBAR/Interpolation_Geometry/evaluate_BSp.m",/* pName */   613     0                                    /* checkKind */   614   };   615      616   static emlrtBCInfo pb_emlrtBCI = { -1, /* iFirst */   617     -1,                                  /* iLast */   618     48,                                  /* lineNo */   619     12,                                  /* colNo */   620     "ders",                              /* aName */   621     "evaluate_BSp",                      /* fName */   622     "/lordvader/doctorands/tozzi/Desktop/Code_NBAR/Interpolation_Geometry/evaluate_BSp.m",/* pName */   623     0                                    /* checkKind */   624   };   625      626   static emlrtBCInfo qb_emlrtBCI = { -1, /* iFirst */   627     -1,                                  /* iLast */   628     30,                                  /* lineNo */   629     18,                                  /* colNo */   630     "U",                                 /* aName */   631     "evaluate_BSp",                      /* fName */   632     "/lordvader/doctorands/tozzi/Desktop/Code_NBAR/Interpolation_Geometry/evaluate_BSp.m",/* pName */   633     0                                    /* checkKind */   634   };   635      636   static emlrtDCInfo o_emlrtDCI = { 30,  /* lineNo */   637     18,                                  /* colNo */   638     "evaluate_BSp",                      /* fName */   639     "/lordvader/doctorands/tozzi/Desktop/Code_NBAR/Interpolation_Geometry/evaluate_BSp.m",/* pName */   640     1                                    /* checkKind */   641   };   642      643   static emlrtBCInfo rb_emlrtBCI = { -1, /* iFirst */   644     -1,                                  /* iLast */   645     30,                                  /* lineNo */   646     5,                                   /* colNo */   647     "left",                              /* aName */   648     "evaluate_BSp",                      /* fName */   649     "/lordvader/doctorands/tozzi/Desktop/Code_NBAR/Interpolation_Geometry/evaluate_BSp.m",/* pName */   650     0                                    /* checkKind */   651   };   652      653   static emlrtBCInfo sb_emlrtBCI = { -1, /* iFirst */   654     -1,                                  /* iLast */   655     31,                                  /* lineNo */   656     17,                                  /* colNo */   657     "U",                                 /* aName */   658     "evaluate_BSp",                      /* fName */   659     "/lordvader/doctorands/tozzi/Desktop/Code_NBAR/Interpolation_Geometry/evaluate_BSp.m",/* pName */   660     0                                    /* checkKind */   661   };   662      663   static emlrtDCInfo p_emlrtDCI = { 31,  /* lineNo */   664     17,                                  /* colNo */   665     "evaluate_BSp",                      /* fName */   666     "/lordvader/doctorands/tozzi/Desktop/Code_NBAR/Interpolation_Geometry/evaluate_BSp.m",/* pName */   667     1                                    /* checkKind */   668   };   669      670   static emlrtBCInfo tb_emlrtBCI = { -1, /* iFirst */   671     -1,                                  /* iLast */   672     31,                                  /* lineNo */   673     5,                                   /* colNo */   674     "right",                             /* aName */   675     "evaluate_BSp",                      /* fName */   676     "/lordvader/doctorands/tozzi/Desktop/Code_NBAR/Interpolation_Geometry/evaluate_BSp.m",/* pName */   677     0                                    /* checkKind */   678   };   679      680   static emlrtBCInfo ub_emlrtBCI = { -1, /* iFirst */   681     -1,                                  /* iLast */   682     42,                                  /* lineNo */   683     12,                                  /* colNo */   684     "Nshape",                            /* aName */   685     "evaluate_BSp",                      /* fName */   686     "/lordvader/doctorands/tozzi/Desktop/Code_NBAR/Interpolation_Geometry/evaluate_BSp.m",/* pName */   687     0                                    /* checkKind */   688   };   689      690   static emlrtBCInfo vb_emlrtBCI = { -1, /* iFirst */   691     -1,                                  /* iLast */   692     42,                                  /* lineNo */   693     16,                                  /* colNo */   694     "Nshape",                            /* aName */   695     "evaluate_BSp",                      /* fName */   696     "/lordvader/doctorands/tozzi/Desktop/Code_NBAR/Interpolation_Geometry/evaluate_BSp.m",/* pName */   697     0                                    /* checkKind */   698   };   699      700   static emlrtBCInfo wb_emlrtBCI = { -1, /* iFirst */   701     -1,                                  /* iLast */   702     36,                                  /* lineNo */   703     34,                                  /* colNo */   704     "right",                             /* aName */   705     "evaluate_BSp",                      /* fName */   706     "/lordvader/doctorands/tozzi/Desktop/Code_NBAR/Interpolation_Geometry/evaluate_BSp.m",/* pName */   707     0                                    /* checkKind */   708   };   709      710   static emlrtBCInfo xb_emlrtBCI = { -1, /* iFirst */   711     -1,                                  /* iLast */   712     36,                                  /* lineNo */   713     46,                                  /* colNo */   714     "left",                              /* aName */   715     "evaluate_BSp",                      /* fName */   716     "/lordvader/doctorands/tozzi/Desktop/Code_NBAR/Interpolation_Geometry/evaluate_BSp.m",/* pName */   717     0                                    /* checkKind */   718   };   719      720   static emlrtBCInfo yb_emlrtBCI = { -1, /* iFirst */   721     -1,                                  /* iLast */   722     36,                                  /* lineNo */   723     16,                                  /* colNo */   724     "Nshape",                            /* aName */   725     "evaluate_BSp",                      /* fName */   726     "/lordvader/doctorands/tozzi/Desktop/Code_NBAR/Interpolation_Geometry/evaluate_BSp.m",/* pName */   727     0                                    /* checkKind */   728   };   729      730   static emlrtBCInfo ac_emlrtBCI = { -1, /* iFirst */   731     -1,                                  /* iLast */   732     36,                                  /* lineNo */   733     20,                                  /* colNo */   734     "Nshape",                            /* aName */   735     "evaluate_BSp",                      /* fName */   736     "/lordvader/doctorands/tozzi/Desktop/Code_NBAR/Interpolation_Geometry/evaluate_BSp.m",/* pName */   737     0                                    /* checkKind */   738   };   739      740   static emlrtBCInfo bc_emlrtBCI = { -1, /* iFirst */   741     -1,                                  /* iLast */   742     38,                                  /* lineNo */   743     40,                                  /* colNo */   744     "right",                             /* aName */   745     "evaluate_BSp",                      /* fName */   746     "/lordvader/doctorands/tozzi/Desktop/Code_NBAR/Interpolation_Geometry/evaluate_BSp.m",/* pName */   747     0                                    /* checkKind */   748   };   749      750   static emlrtBCInfo cc_emlrtBCI = { -1, /* iFirst */   751     -1,                                  /* iLast */   752     38,                                  /* lineNo */   753     16,                                  /* colNo */   754     "Nshape",                            /* aName */   755     "evaluate_BSp",                      /* fName */   756     "/lordvader/doctorands/tozzi/Desktop/Code_NBAR/Interpolation_Geometry/evaluate_BSp.m",/* pName */   757     0                                    /* checkKind */   758   };   759      760   static emlrtBCInfo dc_emlrtBCI = { -1, /* iFirst */   761     -1,                                  /* iLast */   762     38,                                  /* lineNo */   763     19,                                  /* colNo */   764     "Nshape",                            /* aName */   765     "evaluate_BSp",                      /* fName */   766     "/lordvader/doctorands/tozzi/Desktop/Code_NBAR/Interpolation_Geometry/evaluate_BSp.m",/* pName */   767     0                                    /* checkKind */   768   };   769      770   static emlrtBCInfo ec_emlrtBCI = { -1, /* iFirst */   771     -1,                                  /* iLast */   772     39,                                  /* lineNo */   773     17,                                  /* colNo */   774     "left",                              /* aName */   775     "evaluate_BSp",                      /* fName */   776     "/lordvader/doctorands/tozzi/Desktop/Code_NBAR/Interpolation_Geometry/evaluate_BSp.m",/* pName */   777     0                                    /* checkKind */   778   };   779      780   /* Function Definitions */   781   void evaluate_BSp(const emlrtStack *sp, const real_T U_data[], const int32_T   782                     U_size[2], real_T p, real_T de, real_T x, emxArray_real_T   783                     *ders, real_T *b_sp)   784   {   785     real_T n;   786     int32_T i0;   787     int32_T i1;   788     int32_T nxout;   789     emxArray_real_T *Nshape;   790     boolean_T b_data[157];   791     boolean_T idx_data[157];   792     int32_T k;   793     real_T temp;   794     emxArray_real_T *left;   795     emxArray_real_T *right;   796     emxArray_real_T *a;   797     real_T s1;   798     real_T s2;   799     uint32_T r;   800     int32_T ja;   801     real_T d;   802     real_T rk;   803     real_T pk;   804     real_T b_j1;   805     int32_T i2;   806     int32_T i3;   807     int32_T i4;   808     emlrtStack st;   809     emlrtStack b_st;   810     st.prev = sp;   811     st.tls = sp->tls;   812     b_st.prev = &st;   813     b_st.tls = st.tls;   814     emlrtHeapReferenceStackEnterFcnR2012b(sp);   815      816     /*  */   817     /* This function evaluate the values of shape functions and derivatives  */   818     /* at a given point */   819     /*  */   820     n = (((real_T)U_size[1] - 1.0) - p) - 1.0;   821      822     /*  */   823     /* Computing the span */   824     if (n + 1.0 != (int32_T)muDoubleScalarFloor(n + 1.0)) {   825       emlrtIntegerCheckR2012b(n + 1.0, &m_emlrtDCI, sp);   826     }   827      828     i0 = (int32_T)(n + 1.0);   829     if (!((i0 >= 1) && (i0 <= U_size[1]))) {   830       emlrtDynamicBoundsCheckR2012b(i0, 1, U_size[1], &s_emlrtBCI, sp);   831     }   832      833     if (n + 1.0 != (int32_T)muDoubleScalarFloor(n + 1.0)) {   834       emlrtIntegerCheckR2012b(n + 1.0, &n_emlrtDCI, sp);   835     }   836      837     i1 = (int32_T)(n + 1.0);   838     if (!((i1 >= 1) && (i1 <= U_size[1]))) {   839       emlrtDynamicBoundsCheckR2012b(i1, 1, U_size[1], &t_emlrtBCI, sp);   840     }   841      842     if (x >= U_data[i0 - 1] - U_data[i1 - 1] * 1.0E-9) {   843       *b_sp = n + 1.0;   844     } else {   845       nxout = U_size[0] * U_size[1];   846       for (i0 = 0; i0 < nxout; i0++) {   847         b_data[i0] = (x >= U_data[i0]);   848       }   849      850       st.site = &emlrtRSI;   851       nxout = U_size[1];   852       for (i0 = 0; i0 < nxout; i0++) {   853         idx_data[i0] = !b_data[i0];   854       }   855      856       b_st.site = &b_emlrtRSI;   857       k = U_size[1];   858       while ((k >= 1) && (!idx_data[k - 1])) {   859         k--;   860       }   861      862       if (!(k <= U_size[1])) {   863         emlrtErrorWithMessageIdR2012b(&b_st, &n_emlrtRTEI,   864           "MATLAB:subsdeldimmismatch", 0);   865       }   866      867       b_st.site = &c_emlrtRSI;   868       nxout = 0;   869       for (k = 1; k <= U_size[1]; k++) {   870         nxout += idx_data[k - 1];   871       }   872      873       nxout = U_size[1] - nxout;   874       if (!(nxout <= U_size[1])) {   875         emlrtErrorWithMessageIdR2012b(&b_st, &o_emlrtRTEI,   876           "Coder:builtins:AssertionFailed", 0);   877       }   878      879       if (1 > nxout) {   880         *b_sp = 0.0;   881       } else {   882         *b_sp = nxout;   883       }   884     }   885      886     emxInit_real_T(sp, &Nshape, 2, &b_emlrtRTEI, true);   887      888     /*  */   889     /* Computing Shape functions */   890     i0 = Nshape->size[0] * Nshape->size[1];   891     if (!(p + 1.0 >= 0.0)) {   892       emlrtNonNegativeCheckR2012b(p + 1.0, &b_emlrtDCI, sp);   893     }   894      895     n = p + 1.0;   896     if (n != (int32_T)muDoubleScalarFloor(n)) {   897       emlrtIntegerCheckR2012b(n, &emlrtDCI, sp);   898     }   899      900     Nshape->size[0] = (int32_T)n;   901     if (!(p + 1.0 >= 0.0)) {   902       emlrtNonNegativeCheckR2012b(p + 1.0, &d_emlrtDCI, sp);   903     }   904      905     n = p + 1.0;   906     if (n != (int32_T)muDoubleScalarFloor(n)) {   907       emlrtIntegerCheckR2012b(n, &c_emlrtDCI, sp);   908     }   909      910     Nshape->size[1] = (int32_T)n;   911     emxEnsureCapacity(sp, (emxArray__common *)Nshape, i0, (int32_T)sizeof(real_T),   912                       &emlrtRTEI);   913     if (!(p + 1.0 >= 0.0)) {   914       emlrtNonNegativeCheckR2012b(p + 1.0, &b_emlrtDCI, sp);   915     }   916      917     n = p + 1.0;   918     if (n != (int32_T)muDoubleScalarFloor(n)) {   919       emlrtIntegerCheckR2012b(n, &emlrtDCI, sp);   920     }   921      922     if (!(p + 1.0 >= 0.0)) {   923       emlrtNonNegativeCheckR2012b(p + 1.0, &d_emlrtDCI, sp);   924     }   925      926     temp = p + 1.0;   927     if (temp != (int32_T)muDoubleScalarFloor(temp)) {   928       emlrtIntegerCheckR2012b(temp, &c_emlrtDCI, sp);   929     }   930      931     nxout = (int32_T)n * (int32_T)temp;   932     for (i0 = 0; i0 < nxout; i0++) {   933       Nshape->data[i0] = 0.0;   934     }   935      936     emxInit_real_T(sp, &left, 2, &c_emlrtRTEI, true);   937     i0 = left->size[0] * left->size[1];   938     left->size[0] = 1;   939     if (!(p >= 0.0)) {   940       emlrtNonNegativeCheckR2012b(p, &f_emlrtDCI, sp);   941     }   942      943     if (p != (int32_T)muDoubleScalarFloor(p)) {   944       emlrtIntegerCheckR2012b(p, &e_emlrtDCI, sp);   945     }   946      947     left->size[1] = (int32_T)p;   948     emxEnsureCapacity(sp, (emxArray__common *)left, i0, (int32_T)sizeof(real_T),   949                       &emlrtRTEI);   950     if (!(p >= 0.0)) {   951       emlrtNonNegativeCheckR2012b(p, &f_emlrtDCI, sp);   952     }   953      954     if (p != (int32_T)muDoubleScalarFloor(p)) {   955       emlrtIntegerCheckR2012b(p, &e_emlrtDCI, sp);   956     }   957      958     nxout = (int32_T)p;   959     for (i0 = 0; i0 < nxout; i0++) {   960       left->data[i0] = 0.0;   961     }   962      963     emxInit_real_T(sp, &right, 2, &d_emlrtRTEI, true);   964     i0 = right->size[0] * right->size[1];   965     right->size[0] = 1;   966     if (p != (int32_T)muDoubleScalarFloor(p)) {   967       emlrtIntegerCheckR2012b(p, &g_emlrtDCI, sp);   968     }   969      970     right->size[1] = (int32_T)p;   971     emxEnsureCapacity(sp, (emxArray__common *)right, i0, (int32_T)sizeof(real_T),   972                       &emlrtRTEI);   973     if (p != (int32_T)muDoubleScalarFloor(p)) {   974       emlrtIntegerCheckR2012b(p, &g_emlrtDCI, sp);   975     }   976      977     nxout = (int32_T)p;   978     for (i0 = 0; i0 < nxout; i0++) {   979       right->data[i0] = 0.0;   980     }   981      982     i0 = (int32_T)(p + 1.0);   983     if (!(1 <= i0)) {   984       emlrtDynamicBoundsCheckR2012b(1, 1, i0, &emlrtBCI, sp);   985     }   986      987     i0 = (int32_T)(p + 1.0);   988     if (!(1 <= i0)) {   989       emlrtDynamicBoundsCheckR2012b(1, 1, i0, &b_emlrtBCI, sp);   990     }   991      992     Nshape->data[0] = 1.0;   993     i0 = ders->size[0] * ders->size[1];   994     if (!(de + 1.0 >= 0.0)) {   995       emlrtNonNegativeCheckR2012b(de + 1.0, &i_emlrtDCI, sp);   996     }   997      998     n = de + 1.0;   999     if (n != (int32_T)muDoubleScalarFloor(n)) {  1000       emlrtIntegerCheckR2012b(n, &h_emlrtDCI, sp);  1001     }  1002     1003     ders->size[0] = (int32_T)n;  1004     if (p + 1.0 != (int32_T)muDoubleScalarFloor(p + 1.0)) {  1005       emlrtIntegerCheckR2012b(p + 1.0, &j_emlrtDCI, sp);  1006     }  1007     1008     ders->size[1] = (int32_T)(p + 1.0);  1009     emxEnsureCapacity(sp, (emxArray__common *)ders, i0, (int32_T)sizeof(real_T),  1010                       &emlrtRTEI);  1011     if (!(de + 1.0 >= 0.0)) {  1012       emlrtNonNegativeCheckR2012b(de + 1.0, &i_emlrtDCI, sp);  1013     }  1014     1015     n = de + 1.0;  1016     if (n != (int32_T)muDoubleScalarFloor(n)) {  1017       emlrtIntegerCheckR2012b(n, &h_emlrtDCI, sp);  1018     }  1019     1020     if (p + 1.0 != (int32_T)muDoubleScalarFloor(p + 1.0)) {  1021       emlrtIntegerCheckR2012b(p + 1.0, &j_emlrtDCI, sp);  1022     }  1023     1024     nxout = (int32_T)n * (int32_T)(p + 1.0);  1025     for (i0 = 0; i0 < nxout; i0++) {  1026       ders->data[i0] = 0.0;  1027     }  1028     1029     emxInit_real_T(sp, &a, 2, &e_emlrtRTEI, true);  1030     i0 = a->size[0] * a->size[1];  1031     a->size[0] = 2;  1032     if (!(de + 1.0 >= 0.0)) {  1033       emlrtNonNegativeCheckR2012b(de + 1.0, &l_emlrtDCI, sp);  1034     }  1035     1036     n = de + 1.0;  1037     if (n != (int32_T)muDoubleScalarFloor(n)) {  1038       emlrtIntegerCheckR2012b(n, &k_emlrtDCI, sp);  1039     }  1040     1041     a->size[1] = (int32_T)n;  1042     emxEnsureCapacity(sp, (emxArray__common *)a, i0, (int32_T)sizeof(real_T),  1043                       &emlrtRTEI);  1044     if (!(de + 1.0 >= 0.0)) {  1045       emlrtNonNegativeCheckR2012b(de + 1.0, &l_emlrtDCI, sp);  1046     }  1047     1048     n = de + 1.0;  1049     if (n != (int32_T)muDoubleScalarFloor(n)) {  1050       emlrtIntegerCheckR2012b(n, &k_emlrtDCI, sp);  1051     }  1052     1053     nxout = (int32_T)n << 1;  1054     for (i0 = 0; i0 < nxout; i0++) {  1055       a->data[i0] = 0.0;  1056     }  1057     1058     /*  */  1059     emlrtForLoopVectorCheckR2012b(1.0, 1.0, p, mxDOUBLE_CLASS, (int32_T)p,  1060       &g_emlrtRTEI, sp);  1061     nxout = 0;  1062     while (nxout <= (int32_T)p - 1) {  1063       n = (*b_sp + 1.0) - (1.0 + (real_T)nxout);  1064       if (n != (int32_T)muDoubleScalarFloor(n)) {  1065         emlrtIntegerCheckR2012b(n, &o_emlrtDCI, sp);  1066       }  1067     1068       i0 = (int32_T)n;  1069       if (!((i0 >= 1) && (i0 <= U_size[1]))) {  1070         emlrtDynamicBoundsCheckR2012b(i0, 1, U_size[1], &qb_emlrtBCI, sp);  1071       }  1072     1073       i1 = left->size[1];  1074       if (!((nxout + 1 >= 1) && (nxout + 1 <= i1))) {  1075         emlrtDynamicBoundsCheckR2012b(nxout + 1, 1, i1, &rb_emlrtBCI, sp);  1076       }  1077     1078       left->data[nxout] = x - U_data[i0 - 1];  1079       n = *b_sp + (1.0 + (real_T)nxout);  1080       if (n != (int32_T)muDoubleScalarFloor(n)) {  1081         emlrtIntegerCheckR2012b(n, &p_emlrtDCI, sp);  1082       }  1083     1084       i0 = (int32_T)n;  1085       if (!((i0 >= 1) && (i0 <= U_size[1]))) {  1086         emlrtDynamicBoundsCheckR2012b(i0, 1, U_size[1], &sb_emlrtBCI, sp);  1087       }  1088     1089       i1 = right->size[1];  1090       if (!((nxout + 1 >= 1) && (nxout + 1 <= i1))) {  1091         emlrtDynamicBoundsCheckR2012b(nxout + 1, 1, i1, &tb_emlrtBCI, sp);  1092       }  1093     1094       right->data[nxout] = U_data[i0 - 1] - x;  1095       n = 0.0;  1096       r = 0U;  1097       while (r < 1.0 + (real_T)nxout) {  1098         i0 = right->size[1];  1099         i1 = (int32_T)(r + 1U);  1100         if (!((i1 >= 1) && (i1 <= i0))) {  1101           emlrtDynamicBoundsCheckR2012b(i1, 1, i0, &wb_emlrtBCI, sp);  1102         }  1103     1104         i0 = left->size[1];  1105         i2 = (int32_T)((1.0 + (real_T)nxout) - (real_T)r);  1106         if (!((i2 >= 1) && (i2 <= i0))) {  1107           emlrtDynamicBoundsCheckR2012b(i2, 1, i0, &xb_emlrtBCI, sp);  1108         }  1109     1110         i0 = Nshape->size[0];  1111         i3 = (int32_T)((1.0 + (real_T)nxout) + 1.0);  1112         if (!((i3 >= 1) && (i3 <= i0))) {  1113           emlrtDynamicBoundsCheckR2012b(i3, 1, i0, &yb_emlrtBCI, sp);  1114         }  1115     1116         i0 = Nshape->size[1];  1117         i4 = (int32_T)(r + 1U);  1118         if (!((i4 >= 1) && (i4 <= i0))) {  1119           emlrtDynamicBoundsCheckR2012b(i4, 1, i0, &ac_emlrtBCI, sp);  1120         }  1121     1122         Nshape->data[(i3 + Nshape->size[0] * (i4 - 1)) - 1] = right->data  1123           [right->size[0] * (i1 - 1)] + left->data[left->size[0] * (i2 - 1)];  1124         i0 = Nshape->size[0];  1125         i1 = (int32_T)((real_T)r + 1.0);  1126         if (!((i1 >= 1) && (i1 <= i0))) {  1127           emlrtDynamicBoundsCheckR2012b(i1, 1, i0, &c_emlrtBCI, sp);  1128         }  1129     1130         i0 = Nshape->size[1];  1131         i1 = nxout + 1;  1132         if (!((i1 >= 1) && (i1 <= i0))) {  1133           emlrtDynamicBoundsCheckR2012b(i1, 1, i0, &d_emlrtBCI, sp);  1134         }  1135     1136         i0 = Nshape->size[0];  1137         i1 = (int32_T)((1.0 + (real_T)nxout) + 1.0);  1138         if (!((i1 >= 1) && (i1 <= i0))) {  1139           emlrtDynamicBoundsCheckR2012b(i1, 1, i0, &e_emlrtBCI, sp);  1140         }  1141     1142         i0 = Nshape->size[1];  1143         i1 = (int32_T)((real_T)r + 1.0);  1144         if (!((i1 >= 1) && (i1 <= i0))) {  1145           emlrtDynamicBoundsCheckR2012b(i1, 1, i0, &f_emlrtBCI, sp);  1146         }  1147     1148         temp = Nshape->data[((int32_T)((real_T)r + 1.0) + Nshape->size[0] * nxout)  1149           - 1] / Nshape->data[((int32_T)((1.0 + (real_T)nxout) + 1.0) +  1150           Nshape->size[0] * ((int32_T)((real_T)r + 1.0) - 1)) - 1];  1151         i0 = right->size[1];  1152         i1 = (int32_T)(r + 1U);  1153         if (!((i1 >= 1) && (i1 <= i0))) {  1154           emlrtDynamicBoundsCheckR2012b(i1, 1, i0, &bc_emlrtBCI, sp);  1155         }  1156     1157         i0 = Nshape->size[0];  1158         i2 = (int32_T)(r + 1U);  1159         if (!((i2 >= 1) && (i2 <= i0))) {  1160           emlrtDynamicBoundsCheckR2012b(i2, 1, i0, &cc_emlrtBCI, sp);  1161         }  1162     1163         i0 = Nshape->size[1];  1164         i3 = (int32_T)((1.0 + (real_T)nxout) + 1.0);  1165         if (!((i3 >= 1) && (i3 <= i0))) {  1166           emlrtDynamicBoundsCheckR2012b(i3, 1, i0, &dc_emlrtBCI, sp);  1167         }  1168     1169         Nshape->data[(i2 + Nshape->size[0] * (i3 - 1)) - 1] = n + right->  1170           data[right->size[0] * (i1 - 1)] * temp;  1171         i0 = left->size[1];  1172         i1 = (int32_T)((1.0 + (real_T)nxout) - (real_T)r);  1173         if (!((i1 >= 1) && (i1 <= i0))) {  1174           emlrtDynamicBoundsCheckR2012b(i1, 1, i0, &ec_emlrtBCI, sp);  1175         }  1176     1177         n = left->data[i1 - 1] * temp;  1178         r++;  1179         if (*emlrtBreakCheckR2012bFlagVar != 0) {  1180           emlrtBreakCheckR2012b(sp);  1181         }  1182       }  1183     1184       i0 = Nshape->size[0];  1185       i1 = (int32_T)((1.0 + (real_T)nxout) + 1.0);  1186       if (!((i1 >= 1) && (i1 <= i0))) {  1187         emlrtDynamicBoundsCheckR2012b(i1, 1, i0, &ub_emlrtBCI, sp);  1188       }  1189     1190       i0 = Nshape->size[1];  1191       i2 = (int32_T)((1.0 + (real_T)nxout) + 1.0);  1192       if (!((i2 >= 1) && (i2 <= i0))) {  1193         emlrtDynamicBoundsCheckR2012b(i2, 1, i0, &vb_emlrtBCI, sp);  1194       }  1195     1196       Nshape->data[(i1 + Nshape->size[0] * (i2 - 1)) - 1] = n;  1197       nxout++;  1198       if (*emlrtBreakCheckR2012bFlagVar != 0) {  1199         emlrtBreakCheckR2012b(sp);  1200       }  1201     }  1202     1203     emxFree_real_T(&right);  1204     emxFree_real_T(&left);  1205     1206     /*  */  1207     /* Computation of derivatives */  1208     emlrtForLoopVectorCheckR2012b(0.0, 1.0, p, mxDOUBLE_CLASS, (int32_T)(p + 1.0),  1209       &h_emlrtRTEI, sp);  1210     nxout = 1;  1211     while (nxout - 1 <= (int32_T)(p + 1.0) - 1) {  1212       i0 = ders->size[0];  1213       if (!(1 <= i0)) {  1214         emlrtDynamicBoundsCheckR2012b(1, 1, i0, &r_emlrtBCI, sp);  1215       }  1216     1217       i0 = Nshape->size[0];  1218       if (!((nxout >= 1) && (nxout <= i0))) {  1219         emlrtDynamicBoundsCheckR2012b(nxout, 1, i0, &nb_emlrtBCI, sp);  1220       }  1221     1222       i0 = Nshape->size[1];  1223       i1 = (int32_T)(p + 1.0);  1224       if (!((i1 >= 1) && (i1 <= i0))) {  1225         emlrtDynamicBoundsCheckR2012b(i1, 1, i0, &ob_emlrtBCI, sp);  1226       }  1227     1228       i0 = ders->size[1];  1229       if (!((nxout >= 1) && (nxout <= i0))) {  1230         emlrtDynamicBoundsCheckR2012b(nxout, 1, i0, &pb_emlrtBCI, sp);  1231       }  1232     1233       ders->data[ders->size[0] * (nxout - 1)] = Nshape->data[(nxout + Nshape->  1234         size[0] * (i1 - 1)) - 1];  1235       nxout++;  1236       if (*emlrtBreakCheckR2012bFlagVar != 0) {  1237         emlrtBreakCheckR2012b(sp);  1238       }  1239     }  1240     1241     emlrtForLoopVectorCheckR2012b(0.0, 1.0, p, mxDOUBLE_CLASS, (int32_T)(p + 1.0),  1242       &i_emlrtRTEI, sp);  1243     nxout = 0;  1244     while (nxout <= (int32_T)(p + 1.0) - 1) {  1245       s1 = 1.0;  1246       s2 = 2.0;  1247       i0 = a->size[1];  1248       if (!(1 <= i0)) {  1249         emlrtDynamicBoundsCheckR2012b(1, 1, i0, &g_emlrtBCI, sp);  1250       }  1251     1252       a->data[0] = 1.0;  1253       emlrtForLoopVectorCheckR2012b(1.0, 1.0, de, mxDOUBLE_CLASS, (int32_T)de,  1254         &j_emlrtRTEI, sp);  1255       k = 0;  1256       while (k <= (int32_T)de - 1) {  1257         d = 0.0;  1258         rk = (real_T)nxout - (1.0 + (real_T)k);  1259         pk = p - (1.0 + (real_T)k);  1260         if (nxout >= 1.0 + (real_T)k) {  1261           i0 = a->size[1];  1262           if (!(1 <= i0)) {  1263             emlrtDynamicBoundsCheckR2012b(1, 1, i0, &k_emlrtBCI, sp);  1264           }  1265     1266           i0 = a->size[1];  1267           if (!(1 <= i0)) {  1268             emlrtDynamicBoundsCheckR2012b(1, 1, i0, &h_emlrtBCI, sp);  1269           }  1270     1271           i0 = Nshape->size[0];  1272           i1 = (int32_T)((pk + 1.0) + 1.0);  1273           if (!((i1 >= 1) && (i1 <= i0))) {  1274             emlrtDynamicBoundsCheckR2012b(i1, 1, i0, &i_emlrtBCI, sp);  1275           }  1276     1277           i0 = Nshape->size[1];  1278           i1 = (int32_T)(rk + 1.0);  1279           if (!((i1 >= 1) && (i1 <= i0))) {  1280             emlrtDynamicBoundsCheckR2012b(i1, 1, i0, &j_emlrtBCI, sp);  1281           }  1282     1283           a->data[(int32_T)s2 - 1] = a->data[(int32_T)s1 - 1] / Nshape->data  1284             [((int32_T)((pk + 1.0) + 1.0) + Nshape->size[0] * ((int32_T)(rk + 1.0)  1285                - 1)) - 1];  1286           i0 = a->size[1];  1287           if (!(1 <= i0)) {  1288             emlrtDynamicBoundsCheckR2012b(1, 1, i0, &l_emlrtBCI, sp);  1289           }  1290     1291           i0 = Nshape->size[0];  1292           i1 = (int32_T)(rk + 1.0);  1293           if (!((i1 >= 1) && (i1 <= i0))) {  1294             emlrtDynamicBoundsCheckR2012b(i1, 1, i0, &y_emlrtBCI, sp);  1295           }  1296     1297           i0 = Nshape->size[1];  1298           i2 = (int32_T)(pk + 1.0);  1299           if (!((i2 >= 1) && (i2 <= i0))) {  1300             emlrtDynamicBoundsCheckR2012b(i2, 1, i0, &ab_emlrtBCI, sp);  1301           }  1302     1303           d = a->data[(int32_T)s2 - 1] * Nshape->data[(i1 + Nshape->size[0] * (i2  1304             - 1)) - 1];  1305         }  1306     1307         if (rk >= -1.0) {  1308           b_j1 = 1.0;  1309         } else {  1310           b_j1 = -rk;  1311         }  1312     1313         if ((real_T)nxout - 1.0 <= pk) {  1314           n = (1.0 + (real_T)k) - 1.0;  1315         } else {  1316           n = p - (real_T)nxout;  1317         }  1318     1319         i0 = (int32_T)(n + (1.0 - b_j1));  1320         emlrtForLoopVectorCheckR2012b(b_j1, 1.0, n, mxDOUBLE_CLASS, i0,  1321           &k_emlrtRTEI, sp);  1322         ja = 0;  1323         while (ja <= i0 - 1) {  1324           n = b_j1 + (real_T)ja;  1325           i1 = a->size[1];  1326           i2 = (int32_T)(n + 1.0);  1327           if (!((i2 >= 1) && (i2 <= i1))) {  1328             emlrtDynamicBoundsCheckR2012b(i2, 1, i1, &m_emlrtBCI, sp);  1329           }  1330     1331           i1 = a->size[1];  1332           i2 = (int32_T)((n + 1.0) - 1.0);  1333           if (!((i2 >= 1) && (i2 <= i1))) {  1334             emlrtDynamicBoundsCheckR2012b(i2, 1, i1, &n_emlrtBCI, sp);  1335           }  1336     1337           i1 = Nshape->size[0];  1338           i2 = (int32_T)((pk + 1.0) + 1.0);  1339           if (!((i2 >= 1) && (i2 <= i1))) {  1340             emlrtDynamicBoundsCheckR2012b(i2, 1, i1, &hb_emlrtBCI, sp);  1341           }  1342     1343           i1 = Nshape->size[1];  1344           i3 = (int32_T)((rk + 1.0) + n);  1345           if (!((i3 >= 1) && (i3 <= i1))) {  1346             emlrtDynamicBoundsCheckR2012b(i3, 1, i1, &ib_emlrtBCI, sp);  1347           }  1348     1349           temp = Nshape->data[(i2 + Nshape->size[0] * (i3 - 1)) - 1];  1350           i1 = a->size[1];  1351           i2 = (int32_T)(n + 1.0);  1352           if (!((i2 >= 1) && (i2 <= i1))) {  1353             emlrtDynamicBoundsCheckR2012b(i2, 1, i1, &jb_emlrtBCI, sp);  1354           }  1355     1356           a->data[((int32_T)s2 + a->size[0] * (i2 - 1)) - 1] = (a->data[((int32_T)  1357             s1 + a->size[0] * ((int32_T)(n + 1.0) - 1)) - 1] - a->data[((int32_T)  1358             s1 + a->size[0] * ((int32_T)((n + 1.0) - 1.0) - 1)) - 1]) / temp;  1359           i1 = a->size[1];  1360           i2 = (int32_T)(n + 1.0);  1361           if (!((i2 >= 1) && (i2 <= i1))) {  1362             emlrtDynamicBoundsCheckR2012b(i2, 1, i1, &kb_emlrtBCI, sp);  1363           }  1364     1365           i1 = Nshape->size[0];  1366           i3 = (int32_T)((rk + 1.0) + n);  1367           if (!((i3 >= 1) && (i3 <= i1))) {  1368             emlrtDynamicBoundsCheckR2012b(i3, 1, i1, &lb_emlrtBCI, sp);  1369           }  1370     1371           i1 = Nshape->size[1];  1372           i4 = (int32_T)(pk + 1.0);  1373           if (!((i4 >= 1) && (i4 <= i1))) {  1374             emlrtDynamicBoundsCheckR2012b(i4, 1, i1, &mb_emlrtBCI, sp);  1375           }  1376     1377           d += a->data[((int32_T)s2 + a->size[0] * (i2 - 1)) - 1] * Nshape->data  1378             [(i3 + Nshape->size[0] * (i4 - 1)) - 1];  1379           ja++;  1380           if (*emlrtBreakCheckR2012bFlagVar != 0) {  1381             emlrtBreakCheckR2012b(sp);  1382           }  1383         }  1384     1385         if (nxout <= pk) {  1386           i0 = a->size[1];  1387           i1 = (int32_T)(((1.0 + (real_T)k) + 1.0) - 1.0);  1388           if (!((i1 >= 1) && (i1 <= i0))) {  1389             emlrtDynamicBoundsCheckR2012b(i1, 1, i0, &o_emlrtBCI, sp);  1390           }  1391     1392           i0 = Nshape->size[0];  1393           i1 = (int32_T)((pk + 1.0) + 1.0);  1394           if (!((i1 >= 1) && (i1 <= i0))) {  1395             emlrtDynamicBoundsCheckR2012b(i1, 1, i0, &p_emlrtBCI, sp);  1396           }  1397     1398           i0 = Nshape->size[1];  1399           i1 = nxout + 1;  1400           if (!((i1 >= 1) && (i1 <= i0))) {  1401             emlrtDynamicBoundsCheckR2012b(i1, 1, i0, &q_emlrtBCI, sp);  1402           }  1403     1404           i0 = a->size[1];  1405           i1 = (int32_T)((1.0 + (real_T)k) + 1.0);  1406           if (!((i1 >= 1) && (i1 <= i0))) {  1407             emlrtDynamicBoundsCheckR2012b(i1, 1, i0, &bb_emlrtBCI, sp);  1408           }  1409     1410           a->data[((int32_T)s2 + a->size[0] * (i1 - 1)) - 1] = -a->data[((int32_T)  1411             s1 + a->size[0] * ((int32_T)(((1.0 + (real_T)k) + 1.0) - 1.0) - 1)) -  1412             1] / Nshape->data[((int32_T)((pk + 1.0) + 1.0) + Nshape->size[0] *  1413                                nxout) - 1];  1414           i0 = a->size[1];  1415           i1 = (int32_T)((1.0 + (real_T)k) + 1.0);  1416           if (!((i1 >= 1) && (i1 <= i0))) {  1417             emlrtDynamicBoundsCheckR2012b(i1, 1, i0, &cb_emlrtBCI, sp);  1418           }  1419     1420           i0 = Nshape->size[0];  1421           if (!((nxout + 1 >= 1) && (nxout + 1 <= i0))) {  1422             emlrtDynamicBoundsCheckR2012b(nxout + 1, 1, i0, &db_emlrtBCI, sp);  1423           }  1424     1425           i0 = Nshape->size[1];  1426           i2 = (int32_T)(pk + 1.0);  1427           if (!((i2 >= 1) && (i2 <= i0))) {  1428             emlrtDynamicBoundsCheckR2012b(i2, 1, i0, &eb_emlrtBCI, sp);  1429           }  1430     1431           d += a->data[((int32_T)s2 + a->size[0] * (i1 - 1)) - 1] * Nshape->  1432             data[nxout + Nshape->size[0] * (i2 - 1)];  1433         }  1434     1435         i0 = ders->size[0];  1436         i1 = (int32_T)((1.0 + (real_T)k) + 1.0);  1437         if (!((i1 >= 1) && (i1 <= i0))) {  1438           emlrtDynamicBoundsCheckR2012b(i1, 1, i0, &fb_emlrtBCI, sp);  1439         }  1440     1441         i0 = ders->size[1];  1442         if (!((nxout + 1 >= 1) && (nxout + 1 <= i0))) {  1443           emlrtDynamicBoundsCheckR2012b(nxout + 1, 1, i0, &gb_emlrtBCI, sp);  1444         }  1445     1446         ders->data[(i1 + ders->size[0] * nxout) - 1] = d;  1447         n = s1 - 1.0;  1448         s1 = s2;  1449         s2 = n + 1.0;  1450         k++;  1451         if (*emlrtBreakCheckR2012bFlagVar != 0) {  1452           emlrtBreakCheckR2012b(sp);  1453         }  1454       }  1455     1456       nxout++;  1457       if (*emlrtBreakCheckR2012bFlagVar != 0) {  1458         emlrtBreakCheckR2012b(sp);  1459       }  1460     }  1461     1462     emxFree_real_T(&a);  1463     emxFree_real_T(&Nshape);  1464     1465     /*  */  1466     /* Multiplying the derivatives by correction factor */  1467     n = p;  1468     emlrtForLoopVectorCheckR2012b(1.0, 1.0, de, mxDOUBLE_CLASS, (int32_T)de,  1469       &l_emlrtRTEI, sp);  1470     nxout = 0;  1471     while (nxout <= (int32_T)de - 1) {  1472       emlrtForLoopVectorCheckR2012b(0.0, 1.0, p, mxDOUBLE_CLASS, (int32_T)(p + 1.0),  1473         &m_emlrtRTEI, sp);  1474       ja = 1;  1475       while (ja - 1 <= (int32_T)(p + 1.0) - 1) {  1476         i0 = ders->size[0];  1477         i1 = (int32_T)((1.0 + (real_T)nxout) + 1.0);  1478         if (!((i1 >= 1) && (i1 <= i0))) {  1479           emlrtDynamicBoundsCheckR2012b(i1, 1, i0, &u_emlrtBCI, sp);  1480         }  1481     1482         i0 = ders->size[1];  1483         if (!((ja >= 1) && (ja <= i0))) {  1484           emlrtDynamicBoundsCheckR2012b(ja, 1, i0, &v_emlrtBCI, sp);  1485         }  1486     1487         i0 = ders->size[0];  1488         i2 = (int32_T)((1.0 + (real_T)nxout) + 1.0);  1489         if (!((i2 >= 1) && (i2 <= i0))) {  1490           emlrtDynamicBoundsCheckR2012b(i2, 1, i0, &w_emlrtBCI, sp);  1491         }  1492     1493         i0 = ders->size[1];  1494         if (!((ja >= 1) && (ja <= i0))) {  1495           emlrtDynamicBoundsCheckR2012b(ja, 1, i0, &x_emlrtBCI, sp);  1496         }  1497     1498         ders->data[(i2 + ders->size[0] * (ja - 1)) - 1] = ders->data[(i1 +  1499           ders->size[0] * (ja - 1)) - 1] * n;  1500         ja++;  1501         if (*emlrtBreakCheckR2012bFlagVar != 0) {  1502           emlrtBreakCheckR2012b(sp);  1503         }  1504       }  1505     1506       n *= p - (1.0 + (real_T)nxout);  1507       nxout++;  1508       if (*emlrtBreakCheckR2012bFlagVar != 0) {  1509         emlrtBreakCheckR2012b(sp);  1510       }  1511     }  1512     1513     emlrtHeapReferenceStackLeaveFcnR2012b(sp);  1514   }  1515     1516   /* End of code generation (evaluate_BSp.c) */  1517 ``` |
