# Supplementary material for: Dynamic mechanochemical feedback between curved membranes and BAR protein self-organization
Source: Nat Commun. 2021 Nov 12;12:6550. doi: 10.1038/s41467-021-26591-3 (PMC8589976; doi:10.1038/s41467-021-26591-3)
Supplement: Supplementary file 25 — Supplementary software 1 [file 41467_2021_26591_MOESM25_ESM.zip › Supplementary Software 1/Interpolation_Geometry/codegen/mex/evaluate_BSp/html/evaluate_BSp_data_c.html]

RTW Report - evaluate\_BSp\_data.c


|  |
| --- |
| File: evaluate\_BSp\_data.c  ```     1   /*     2    * Academic License - for use in teaching, academic research, and meeting     3    * course requirements at degree granting institutions only.  Not for     4    * government, commercial, or other organizational use.     5    *     6    * evaluate_BSp_data.c     7    *     8    * Code generation for function 'evaluate_BSp_data'     9    *    10    */    11       12   /* Include files */    13   #include "rt_nonfinite.h"    14   #include "evaluate_BSp.h"    15   #include "evaluate_BSp_data.h"    16       17   /* Variable Definitions */    18   emlrtCTX emlrtRootTLSGlobal = NULL;    19   const volatile char_T *emlrtBreakCheckR2012bFlagVar = NULL;    20   emlrtContext emlrtContextGlobal = { true,/* bFirstTime */    21     false,                               /* bInitialized */    22     131435U,                             /* fVersionInfo */    23     NULL,                                /* fErrorFunction */    24     "evaluate_BSp",                      /* fFunctionName */    25     NULL,                                /* fRTCallStack */    26     false,                               /* bDebugMode */    27     { 2045744189U, 2170104910U, 2743257031U, 4284093946U },/* fSigWrd */    28     NULL                                 /* fSigMem */    29   };    30       31   /* End of code generation (evaluate_BSp_data.c) */    32 ``` |
