# Supplementary material for: Dynamic mechanochemical feedback between curved membranes and BAR protein self-organization
Source: Nat Commun. 2021 Nov 12;12:6550. doi: 10.1038/s41467-021-26591-3 (PMC8589976; doi:10.1038/s41467-021-26591-3)
Supplement: Supplementary file 25 — Supplementary software 1 [file 41467_2021_26591_MOESM25_ESM.zip › Supplementary Software 1/Interpolation_Geometry/codegen/mex/evaluate_BSp/html/evaluate_BSp_emxutil_c.html]

RTW Report - evaluate\_BSp\_emxutil.c


|  |
| --- |
| File: evaluate\_BSp\_emxutil.c  ```     1   /*     2    * Academic License - for use in teaching, academic research, and meeting     3    * course requirements at degree granting institutions only.  Not for     4    * government, commercial, or other organizational use.     5    *     6    * evaluate_BSp_emxutil.c     7    *     8    * Code generation for function 'evaluate_BSp_emxutil'     9    *    10    */    11       12   /* Include files */    13   #include "rt_nonfinite.h"    14   #include "evaluate_BSp.h"    15   #include "evaluate_BSp_emxutil.h"    16       17   /* Function Definitions */    18   void emxEnsureCapacity(const emlrtStack *sp, emxArray__common *emxArray, int32_T    19     oldNumel, int32_T elementSize, const emlrtRTEInfo *srcLocation)    20   {    21     int32_T newNumel;    22     int32_T i;    23     void *newData;    24     if (oldNumel < 0) {    25       oldNumel = 0;    26     }    27       28     newNumel = 1;    29     for (i = 0; i < emxArray->numDimensions; i++) {    30       newNumel = (int32_T)emlrtSizeMulR2012b((uint32_T)newNumel, (uint32_T)    31         emxArray->size[i], srcLocation, sp);    32     }    33       34     if (newNumel > emxArray->allocatedSize) {    35       i = emxArray->allocatedSize;    36       if (i < 16) {    37         i = 16;    38       }    39       40       while (i < newNumel) {    41         if (i > 1073741823) {    42           i = MAX_int32_T;    43         } else {    44           i <<= 1;    45         }    46       }    47       48       newData = emlrtCallocMex((uint32_T)i, (uint32_T)elementSize);    49       if (newData == NULL) {    50         emlrtHeapAllocationErrorR2012b(srcLocation, sp);    51       }    52       53       if (emxArray->data != NULL) {    54         memcpy(newData, emxArray->data, (uint32_T)(elementSize * oldNumel));    55         if (emxArray->canFreeData) {    56           emlrtFreeMex(emxArray->data);    57         }    58       }    59       60       emxArray->data = newData;    61       emxArray->allocatedSize = i;    62       emxArray->canFreeData = true;    63     }    64   }    65       66   void emxFree_real_T(emxArray_real_T **pEmxArray)    67   {    68     if (*pEmxArray != (emxArray_real_T *)NULL) {    69       if (((*pEmxArray)->data != (real_T *)NULL) && (*pEmxArray)->canFreeData) {    70         emlrtFreeMex((void *)(*pEmxArray)->data);    71       }    72       73       emlrtFreeMex((void *)(*pEmxArray)->size);    74       emlrtFreeMex((void *)*pEmxArray);    75       *pEmxArray = (emxArray_real_T *)NULL;    76     }    77   }    78       79   void emxInit_real_T(const emlrtStack *sp, emxArray_real_T **pEmxArray, int32_T    80                       numDimensions, const emlrtRTEInfo *srcLocation, boolean_T    81                       doPush)    82   {    83     emxArray_real_T *emxArray;    84     int32_T i;    85     *pEmxArray = (emxArray_real_T *)emlrtMallocMex(sizeof(emxArray_real_T));    86     if ((void *)*pEmxArray == NULL) {    87       emlrtHeapAllocationErrorR2012b(srcLocation, sp);    88     }    89       90     if (doPush) {    91       emlrtPushHeapReferenceStackR2012b(sp, (void *)pEmxArray, (void (*)(void *))    92         emxFree_real_T);    93     }    94       95     emxArray = *pEmxArray;    96     emxArray->data = (real_T *)NULL;    97     emxArray->numDimensions = numDimensions;    98     emxArray->size = (int32_T *)emlrtMallocMex((uint32_T)(sizeof(int32_T)    99       * numDimensions));   100     if ((void *)emxArray->size == NULL) {   101       emlrtHeapAllocationErrorR2012b(srcLocation, sp);   102     }   103      104     emxArray->allocatedSize = 0;   105     emxArray->canFreeData = true;   106     for (i = 0; i < numDimensions; i++) {   107       emxArray->size[i] = 0;   108     }   109   }   110      111   /* End of code generation (evaluate_BSp_emxutil.c) */   112 ``` |
