# Supplementary material for: Dynamic mechanochemical feedback between curved membranes and BAR protein self-organization
Source: Nat Commun. 2021 Nov 12;12:6550. doi: 10.1038/s41467-021-26591-3 (PMC8589976; doi:10.1038/s41467-021-26591-3)
Supplement: Supplementary file 25 — Supplementary software 1 [file 41467_2021_26591_MOESM25_ESM.zip › Supplementary Software 1/Interpolation_Geometry/codegen/mex/evaluate_BSp/html/evaluate_BSp_initialize_h.html]

RTW Report - evaluate\_BSp\_initialize.h


|  |
| --- |
| File: evaluate\_BSp\_initialize.h  ```     1   /*     2    * Academic License - for use in teaching, academic research, and meeting     3    * course requirements at degree granting institutions only.  Not for     4    * government, commercial, or other organizational use.     5    *     6    * evaluate_BSp_initialize.h     7    *     8    * Code generation for function 'evaluate_BSp_initialize'     9    *    10    */    11       12   #ifndef EVALUATE_BSP_INITIALIZE_H    13   #define EVALUATE_BSP_INITIALIZE_H    14       15   /* Include files */    16   #include <math.h>    17   #include <stdio.h>    18   #include <stdlib.h>    19   #include <string.h>    20   #include "mwmathutil.h"    21   #include "tmwtypes.h"    22   #include "mex.h"    23   #include "emlrt.h"    24   #include "rtwtypes.h"    25   #include "evaluate_BSp_types.h"    26       27   /* Function Declarations */    28   extern void evaluate_BSp_initialize(void);    29       30   #endif    31       32   /* End of code generation (evaluate_BSp_initialize.h) */    33 ``` |
