# Supplementary material for: Dynamic mechanochemical feedback between curved membranes and BAR protein self-organization
Source: Nat Commun. 2021 Nov 12;12:6550. doi: 10.1038/s41467-021-26591-3 (PMC8589976; doi:10.1038/s41467-021-26591-3)
Supplement: Supplementary file 25 — Supplementary software 1 [file 41467_2021_26591_MOESM25_ESM.zip › Supplementary Software 1/Interpolation_Geometry/codegen/mex/evaluate_BSp/html/evaluate_BSp_terminate_c.html]

RTW Report - evaluate\_BSp\_terminate.c


|  |
| --- |
| File: evaluate\_BSp\_terminate.c  ```     1   /*     2    * Academic License - for use in teaching, academic research, and meeting     3    * course requirements at degree granting institutions only.  Not for     4    * government, commercial, or other organizational use.     5    *     6    * evaluate_BSp_terminate.c     7    *     8    * Code generation for function 'evaluate_BSp_terminate'     9    *    10    */    11       12   /* Include files */    13   #include "rt_nonfinite.h"    14   #include "evaluate_BSp.h"    15   #include "evaluate_BSp_terminate.h"    16   #include "_coder_evaluate_BSp_mex.h"    17   #include "evaluate_BSp_data.h"    18       19   /* Function Definitions */    20   void evaluate_BSp_atexit(void)    21   {    22     emlrtStack st = { NULL,              /* site */    23       NULL,                              /* tls */    24       NULL                               /* prev */    25     };    26       27     mexFunctionCreateRootTLS();    28     st.tls = emlrtRootTLSGlobal;    29     emlrtEnterRtStackR2012b(&st);    30     emlrtLeaveRtStackR2012b(&st);    31     emlrtDestroyRootTLS(&emlrtRootTLSGlobal);    32   }    33       34   void evaluate_BSp_terminate(void)    35   {    36     emlrtStack st = { NULL,              /* site */    37       NULL,                              /* tls */    38       NULL                               /* prev */    39     };    40       41     st.tls = emlrtRootTLSGlobal;    42     emlrtLeaveRtStackR2012b(&st);    43     emlrtDestroyRootTLS(&emlrtRootTLSGlobal);    44   }    45       46   /* End of code generation (evaluate_BSp_terminate.c) */    47 ``` |
