# Supplementary material for: Dynamic mechanochemical feedback between curved membranes and BAR protein self-organization
Source: Nat Commun. 2021 Nov 12;12:6550. doi: 10.1038/s41467-021-26591-3 (PMC8589976; doi:10.1038/s41467-021-26591-3)
Supplement: Supplementary file 25 — Supplementary software 1 [file 41467_2021_26591_MOESM25_ESM.zip › Supplementary Software 1/Interpolation_Geometry/codegen/mex/evaluate_BSp/html/evaluate_BSp_types_h.html]

RTW Report - evaluate\_BSp\_types.h


|  |
| --- |
| File: evaluate\_BSp\_types.h  ```     1   /*     2    * Academic License - for use in teaching, academic research, and meeting     3    * course requirements at degree granting institutions only.  Not for     4    * government, commercial, or other organizational use.     5    *     6    * evaluate_BSp_types.h     7    *     8    * Code generation for function 'evaluate_BSp'     9    *    10    */    11       12   #ifndef EVALUATE_BSP_TYPES_H    13   #define EVALUATE_BSP_TYPES_H    14       15   /* Include files */    16   #include "rtwtypes.h"    17       18   /* Type Definitions */    19   #ifndef struct_emxArray__common    20   #define struct_emxArray__common    21       22   struct emxArray__common    23   {    24     void *data;    25     int32_T *size;    26     int32_T allocatedSize;    27     int32_T numDimensions;    28     boolean_T canFreeData;    29   };    30       31   #endif                                 /*struct_emxArray__common*/    32       33   #ifndef typedef_emxArray__common    34   #define typedef_emxArray__common    35       36   typedef struct emxArray__common emxArray__common;    37       38   #endif                                 /*typedef_emxArray__common*/    39       40   #ifndef struct_emxArray_real_T    41   #define struct_emxArray_real_T    42       43   struct emxArray_real_T    44   {    45     real_T *data;    46     int32_T *size;    47     int32_T allocatedSize;    48     int32_T numDimensions;    49     boolean_T canFreeData;    50   };    51       52   #endif                                 /*struct_emxArray_real_T*/    53       54   #ifndef typedef_emxArray_real_T    55   #define typedef_emxArray_real_T    56       57   typedef struct emxArray_real_T emxArray_real_T;    58       59   #endif                                 /*typedef_emxArray_real_T*/    60   #endif    61       62   /* End of code generation (evaluate_BSp_types.h) */    63 ``` |
