# Supplementary material for: Dynamic mechanochemical feedback between curved membranes and BAR protein self-organization
Source: Nat Commun. 2021 Nov 12;12:6550. doi: 10.1038/s41467-021-26591-3 (PMC8589976; doi:10.1038/s41467-021-26591-3)
Supplement: Supplementary file 25 — Supplementary software 1 [file 41467_2021_26591_MOESM25_ESM.zip › Supplementary Software 1/Interpolation_Geometry/codegen/mex/evaluate_BSp/html/evaluate_BSp1_mcode.html]

```
  1function [ders,sp]=evaluate_BSp(U,p,de,x)
  2%
  3%This function evaluate the values of shape functions and derivatives 
  4%at a given point
  5%
  6m = length(U)-1;
  7n = m-p-1;
  8mm = m+1;
  9pm = p+1;
 10%
 11%Computing the span
 12if x>=U(n+1)-(U(n+1)*1e-9)
 13    sp = n+1;
 14else
 15    b=x>=U;
 16    b(b==0)=[];
 17    sp=(length(b));
 18end
 19%
 20%Computing Shape functions
 21Nshape = zeros(pm,pm);
 22left = zeros(1,p);
 23right = zeros(1,p);
 24Nshape(1,1) = 1;
 25
 26ders= zeros(de+1,p+1); 
 27a = zeros(2,de+1);
 28%
 29for j = 1:p
 30    left(j) = (x-U(sp+1-j));
 31    right(j) = (U(sp+j)-x);
 32    saved =0;
 33    r=0;
 34    while r<j;
 35        rm = r+1;
 36        Nshape(j+1,rm) = right(1,r+1)+left(1,j-r);
 37        temp = Nshape(rm,j)/Nshape(j+1,rm);
 38        Nshape(rm,j+1) = saved+right(1,r+1)*temp;
 39        saved = left(j-r)*temp;
 40        r=rm;
 41    end
 42    Nshape(j+1,j+1) = saved;
 43end
 44%
 45%Computation of derivatives
 46for j = 0:1:p
 47    jm = j+1;
 48    ders(1,jm) = Nshape(jm,pm);
 49end
 50for r = 0:1:p
 51    rm = r+1;
 52    s1 = 1;
 53    s2 = 2;
 54    a(1,1) = 1;
 55    for k = 1:de
 56        km = k+1;
 57        d = 0;
 58        rk = r-k;
 59        rkm = rk+1;
 60        pk = p-k;
 61        pkm = pk+1;
 62        if r >= k
 63            a(s2,1) = a(s1,1)/Nshape(pkm+1,rkm);
 64            d = a(s2,1)*Nshape(rkm,pkm);
 65        end
 66        if rk >= -1
 67            j1 = 1;
 68        else
 69            j1 = -rk;
 70        end
 71        if (r-1) <= pk
 72            j2 = k-1;
 73        else
 74            j2 = p-r;
 75        end
 76        for l = j1:j2
 77            lm = l+1;
 78            a(s2,lm) = (a(s1,lm)-a(s1,lm-1))/Nshape(pkm+1,rkm+l);
 79            d = d+a(s2,lm)*Nshape(rkm+l,pkm);
 80        end
 81        if r<= pk
 82            a(s2,km) = -a(s1,km-1)/Nshape(pkm+1,rm);
 83            d = d+a(s2,km)*Nshape(rm,pkm);
 84        end
 85        ders(km,rm) = d;
 86        j = s1-1;
 87        s1 = s2;
 88        s2 = j+1;
 89    end
 90end
 91%
 92%Multiplying the derivatives by correction factor
 93ra = p;
 94for ka = 1:de
 95    kam = ka+1;
 96    for ja = 0:p
 97        jam = ja+1;
 98        ders(kam,jam)= ders(kam,jam)*ra;
 99    end
100    ra = ra*(p-ka);
101end
102
103
104
```
