# Supplementary material for: Dynamic mechanochemical feedback between curved membranes and BAR protein self-organization
Source: Nat Commun. 2021 Nov 12;12:6550. doi: 10.1038/s41467-021-26591-3 (PMC8589976; doi:10.1038/s41467-021-26591-3)
Supplement: Supplementary file 25 — Supplementary software 1 [file 41467_2021_26591_MOESM25_ESM.zip › Supplementary Software 1/Interpolation_Geometry/codegen/mex/evaluate_BSp/html/evaluate_BSp1_watch.html]

|  | Order | Variable | Type | Size | Class | Complex |
| --- | --- | --- | --- | --- | --- | --- |
|  | 1 | ders | Output | **:**? x **:**? | double | No |
|  | 2 | sp | Output | 1 x 1 | double | No |
|  | 3 | U | Input | 1 x **:**157 | double | No |
|  | 4 | p | Input | 1 x 1 | double | No |
|  | 5 | de | Input | 1 x 1 | double | No |
|  | 6 | x | Input | 1 x 1 | double | No |
|  | 7 | m | Local | 1 x 1 | double | No |
|  | 8 | n | Local | 1 x 1 | double | No |
|  | 9 | pm | Local | 1 x 1 | double | No |
|  | 10 | b | Local | 1 x **:**157 | logical | - |
|  | 11 | Nshape | Local | **:**? x **:**? | double | No |
|  | 12 | left | Local | 1 x **:**? | double | No |
|  | 13 | right | Local | 1 x **:**? | double | No |
|  | 14 | a | Local | 2 x **:**? | double | No |
|  | 15 | j | Local | 1 x 1 | double | No |
|  | 16 | saved | Local | 1 x 1 | double | No |
|  | 17 | r | Local | 1 x 1 | double | No |
|  | 18 | rm | Local | 1 x 1 | double | No |
|  | 19 | temp | Local | 1 x 1 | double | No |
|  | 20 | jm | Local | 1 x 1 | double | No |
|  | 21 | s1 | Local | 1 x 1 | double | No |
|  | 22 | s2 | Local | 1 x 1 | double | No |
|  | 23 | k | Local | 1 x 1 | double | No |
|  | 24 | km | Local | 1 x 1 | double | No |
|  | 25 | d | Local | 1 x 1 | double | No |
|  | 26 | rk | Local | 1 x 1 | double | No |
|  | 27 | rkm | Local | 1 x 1 | double | No |
|  | 28 | pk | Local | 1 x 1 | double | No |
|  | 29 | pkm | Local | 1 x 1 | double | No |
|  | 30 | j1 | Local | 1 x 1 | double | No |
|  | 31 | j2 | Local | 1 x 1 | double | No |
|  | 32 | l | Local | 1 x 1 | double | No |
|  | 33 | lm | Local | 1 x 1 | double | No |
|  | 34 | ra | Local | 1 x 1 | double | No |
|  | 35 | ka | Local | 1 x 1 | double | No |
|  | 36 | kam | Local | 1 x 1 | double | No |
|  | 37 | ja | Local | 1 x 1 | double | No |
|  | 38 | jam | Local | 1 x 1 | double | No |
