# Supplementary material for: Dynamic mechanochemical feedback between curved membranes and BAR protein self-organization
Source: Nat Commun. 2021 Nov 12;12:6550. doi: 10.1038/s41467-021-26591-3 (PMC8589976; doi:10.1038/s41467-021-26591-3)
Supplement: Supplementary file 25 — Supplementary software 1 [file 41467_2021_26591_MOESM25_ESM.zip › Supplementary Software 1/Interpolation_Geometry/codegen/mex/evaluate_BSp/html/index.html]

Code Generation Report


- MATLAB code
- Call stack
- C code

Filter

|  |  |
| --- | --- |
| Filter functions and methods | |
| Filter by: | Size Complexity Class |
| Filter: | 1 x 1 1 x **:**157 1 x **:**? 2 x **:**? **:**? x **:**?  Complex values Real values  double logical |

Functions

1. evaluate\_BSp

1. evaluate\_BSp

1. evaluate\_BSp


Target Source Files

|  |
| --- |
| evaluate\_BSp.c |
| evaluate\_BSp.h |
| evaluate\_BSp\_data.c |
| evaluate\_BSp\_data.h |
| evaluate\_BSp\_emxutil.c |
| evaluate\_BSp\_emxutil.h |
| evaluate\_BSp\_initialize.c |
| evaluate\_BSp\_initialize.h |
| evaluate\_BSp\_terminate.c |
| evaluate\_BSp\_terminate.h |
| evaluate\_BSp\_types.h |
| rt\_nonfinite.h |
| rtwtypes.h |

Interface Source Files

|  |
| --- |
| \_coder\_evaluate\_BSp\_api.c |
| \_coder\_evaluate\_BSp\_api.h |
| \_coder\_evaluate\_BSp\_info.c |
| \_coder\_evaluate\_BSp\_info.h |
| \_coder\_evaluate\_BSp\_mex.c |
| \_coder\_evaluate\_BSp\_mex.h |

|  |  |
| --- | --- |
| Size | **:**? x **:**? |
| Class | double |
| Complex | No |

|  |  |
| --- | --- |
| Size | 1 x 1 |
| Class | double |
| Complex | No |

|  |  |
| --- | --- |
| Size | 1 x **:**157 |
| Class | double |
| Complex | No |

|  |  |
| --- | --- |
| Size | 1 x 1 |
| Class | logical |

|  |  |
| --- | --- |
| Size | 1 x **:**157 |
| Class | logical |

|  |  |
| --- | --- |
| Size | 1 x **:**? |
| Class | double |
| Complex | No |

|  |  |
| --- | --- |
| Size | 2 x **:**? |
| Class | double |
| Complex | No |

|  |  |
| --- | --- |
| Size | 1 x 1 |
| Class | double |
| Complex | No |

|  |  |
| --- | --- |
| Size | 1 x 2 |
| Class | cell |

|  |  |
| --- | --- |
| Size | 1 x **:**? |
| Class | logical |

|  |  |
| --- | --- |
| Size | 1 x 23 |
| Class | char |

|  |  |
| --- | --- |
| Size | 1 x 5 |
| Class | char |

|  |  |
| --- | --- |
| Size | 1 x **:**157 |
| Class | double |
| Complex | No |

- Summary
- All Messages (0)
- Variables
- Variables
- Target Build Log

|  |  |
| --- | --- |
| C source code generated on: | 16-Jan-2020 10:27:42 |
| Coding target: | MEX Function |
| Number of errors: | 0 |
| Number of warnings: | 0 |
| Number of notices: | 0 |

|  |
| --- |
| **Tell Us What You Think** |
| We value your feedback. Please take a few minutes to answer this short questionnaire regarding the Code Generation Report. |
| >>Provide Feedback |

Code generation successful. Click here to view summary.

Loading Variables table ...

|  |  |
| --- | --- |
| Build Parameters | |
| Build directory | /lordvader/doctorands/tozzi/Desktop/Code\_NBAR/Interpolation\_Geometry/codegen/mex/evaluate\_BSp |
| Make wrapper | sh evaluate\_BSp\_mex.sh |

  

|  |
| --- |
| Build Log |
| ```     1   /usr/bin/gcc -c -ansi -fexceptions -fPIC -fno-omit-frame-pointer -pthread -D_GNU_SOURCE -DMATLAB_MEX_FILE   -O -DNDEBUG    -I "/opt/MATLAB/R2016b/simulink/include" -I "/opt/MATLAB/R2016b/toolbox/shared/simtargets" -I "/lordvader/doctorands/tozzi/Desktop/Code_NBAR/Interpolation_Geometry" -I "/lordvader/doctorands/tozzi/Desktop/Code_NBAR/Interpolation_Geometry/codegen/mex/evaluate_BSp" -I "./interface" -I "/opt/MATLAB/R2016b/extern/include" -I "." "evaluate_BSp_data.c"     2   /usr/bin/gcc -c -ansi -fexceptions -fPIC -fno-omit-frame-pointer -pthread -D_GNU_SOURCE -DMATLAB_MEX_FILE   -O -DNDEBUG    -I "/opt/MATLAB/R2016b/simulink/include" -I "/opt/MATLAB/R2016b/toolbox/shared/simtargets" -I "/lordvader/doctorands/tozzi/Desktop/Code_NBAR/Interpolation_Geometry" -I "/lordvader/doctorands/tozzi/Desktop/Code_NBAR/Interpolation_Geometry/codegen/mex/evaluate_BSp" -I "./interface" -I "/opt/MATLAB/R2016b/extern/include" -I "." "evaluate_BSp_initialize.c"     3   /usr/bin/gcc -c -ansi -fexceptions -fPIC -fno-omit-frame-pointer -pthread -D_GNU_SOURCE -DMATLAB_MEX_FILE   -O -DNDEBUG    -I "/opt/MATLAB/R2016b/simulink/include" -I "/opt/MATLAB/R2016b/toolbox/shared/simtargets" -I "/lordvader/doctorands/tozzi/Desktop/Code_NBAR/Interpolation_Geometry" -I "/lordvader/doctorands/tozzi/Desktop/Code_NBAR/Interpolation_Geometry/codegen/mex/evaluate_BSp" -I "./interface" -I "/opt/MATLAB/R2016b/extern/include" -I "." "evaluate_BSp_terminate.c"     4   /usr/bin/gcc -c -ansi -fexceptions -fPIC -fno-omit-frame-pointer -pthread -D_GNU_SOURCE -DMATLAB_MEX_FILE   -O -DNDEBUG    -I "/opt/MATLAB/R2016b/simulink/include" -I "/opt/MATLAB/R2016b/toolbox/shared/simtargets" -I "/lordvader/doctorands/tozzi/Desktop/Code_NBAR/Interpolation_Geometry" -I "/lordvader/doctorands/tozzi/Desktop/Code_NBAR/Interpolation_Geometry/codegen/mex/evaluate_BSp" -I "./interface" -I "/opt/MATLAB/R2016b/extern/include" -I "." "evaluate_BSp.c"     5   /usr/bin/gcc -c -ansi -fexceptions -fPIC -fno-omit-frame-pointer -pthread -D_GNU_SOURCE -DMATLAB_MEX_FILE   -O -DNDEBUG    -I "/opt/MATLAB/R2016b/simulink/include" -I "/opt/MATLAB/R2016b/toolbox/shared/simtargets" -I "/lordvader/doctorands/tozzi/Desktop/Code_NBAR/Interpolation_Geometry" -I "/lordvader/doctorands/tozzi/Desktop/Code_NBAR/Interpolation_Geometry/codegen/mex/evaluate_BSp" -I "./interface" -I "/opt/MATLAB/R2016b/extern/include" -I "." "interface/_coder_evaluate_BSp_info.c"     6   /usr/bin/gcc -c -ansi -fexceptions -fPIC -fno-omit-frame-pointer -pthread -D_GNU_SOURCE -DMATLAB_MEX_FILE   -O -DNDEBUG    -I "/opt/MATLAB/R2016b/simulink/include" -I "/opt/MATLAB/R2016b/toolbox/shared/simtargets" -I "/lordvader/doctorands/tozzi/Desktop/Code_NBAR/Interpolation_Geometry" -I "/lordvader/doctorands/tozzi/Desktop/Code_NBAR/Interpolation_Geometry/codegen/mex/evaluate_BSp" -I "./interface" -I "/opt/MATLAB/R2016b/extern/include" -I "." "interface/_coder_evaluate_BSp_api.c"     7   /usr/bin/gcc -c -ansi -fexceptions -fPIC -fno-omit-frame-pointer -pthread -D_GNU_SOURCE -DMATLAB_MEX_FILE   -O -DNDEBUG    -I "/opt/MATLAB/R2016b/simulink/include" -I "/opt/MATLAB/R2016b/toolbox/shared/simtargets" -I "/lordvader/doctorands/tozzi/Desktop/Code_NBAR/Interpolation_Geometry" -I "/lordvader/doctorands/tozzi/Desktop/Code_NBAR/Interpolation_Geometry/codegen/mex/evaluate_BSp" -I "./interface" -I "/opt/MATLAB/R2016b/extern/include" -I "." "interface/_coder_evaluate_BSp_mex.c"     8   /usr/bin/gcc -c -ansi -fexceptions -fPIC -fno-omit-frame-pointer -pthread -D_GNU_SOURCE -DMATLAB_MEX_FILE   -O -DNDEBUG    -I "/opt/MATLAB/R2016b/simulink/include" -I "/opt/MATLAB/R2016b/toolbox/shared/simtargets" -I "/lordvader/doctorands/tozzi/Desktop/Code_NBAR/Interpolation_Geometry" -I "/lordvader/doctorands/tozzi/Desktop/Code_NBAR/Interpolation_Geometry/codegen/mex/evaluate_BSp" -I "./interface" -I "/opt/MATLAB/R2016b/extern/include" -I "." "evaluate_BSp_emxutil.c"     9   /usr/bin/gcc -Wl,--version-script,evaluate_BSp_mex_mex.map evaluate_BSp_data.o evaluate_BSp_initialize.o evaluate_BSp_terminate.o evaluate_BSp.o _coder_evaluate_BSp_info.o _coder_evaluate_BSp_api.o _coder_evaluate_BSp_mex.o evaluate_BSp_emxutil.o -pthread -Wl,--no-undefined -Wl,-rpath-link,/opt/MATLAB/R2016b/bin/glnxa64 -shared -L/opt/MATLAB/R2016b/bin/glnxa64 -lmx -lmex -lmat -lm -lstdc++    -o evaluate_BSp_mex.mexa64    -lemlrt -lcovrt -lut -lmwmathutil ``` |

Calls:

Select a function call:
