# Supplementary material for: Dynamic mechanochemical feedback between curved membranes and BAR protein self-organization
Source: Nat Commun. 2021 Nov 12;12:6550. doi: 10.1038/s41467-021-26591-3 (PMC8589976; doi:10.1038/s41467-021-26591-3)
Supplement: Supplementary file 25 — Supplementary software 1 [file 41467_2021_26591_MOESM25_ESM.zip › Supplementary Software 1/Interpolation_Geometry/codegen/mex/evaluate_BSp/html/mex_warning.html]

Code Generation Report


|  |
| --- |
| The C code that is generated for a MEX file contains extra checks and MATLAB interfacing logic that is not suitable for production code. To generate production code that is more readable and usable outside MATLAB, change the output type to library or executable.  See MEX source code |
