# Supplementary material for: Dynamic mechanochemical feedback between curved membranes and BAR protein self-organization
Source: Nat Commun. 2021 Nov 12;12:6550. doi: 10.1038/s41467-021-26591-3 (PMC8589976; doi:10.1038/s41467-021-26591-3)
Supplement: Supplementary file 25 — Supplementary software 1 [file 41467_2021_26591_MOESM25_ESM.zip › Supplementary Software 1/Interpolation_Geometry/codegen/mex/evaluate_BSp/html/rt_nonfinite_h.html]

RTW Report - rt\_nonfinite.h


|  |
| --- |
| File: rt\_nonfinite.h  ```     1   /*     2    * Academic License - for use in teaching, academic research, and meeting     3    * course requirements at degree granting institutions only.  Not for     4    * government, commercial, or other organizational use.     5    *     6    * rt_nonfinite.h     7    *     8    * Code generation for function 'evaluate_BSp'     9    *    10    */    11       12   #ifndef RT_NONFINITE_H    13   #define RT_NONFINITE_H    14   #define rtInf      	mxGetInf()    15   #define rtMinusInf 	(-mxGetInf())    16   #define rtNaN      	mxGetNaN()    17   #define rtIsNaN(X) 	mxIsNaN(X)    18   #define rtIsInf(X) 	mxIsInf(X)    19   #define rtIsNaNF(X)	mxIsNaN(X)    20   #define rtIsInfF(X)	mxIsInf(X)    21   #endif    22   /* End of code generation (rt_nonfinite.h) */    23 ``` |
