# Supplementary material for: Dynamic mechanochemical feedback between curved membranes and BAR protein self-organization
Source: Nat Commun. 2021 Nov 12;12:6550. doi: 10.1038/s41467-021-26591-3 (PMC8589976; doi:10.1038/s41467-021-26591-3)
Supplement: Supplementary file 25 — Supplementary software 1 [file 41467_2021_26591_MOESM25_ESM.zip › Supplementary Software 1/Interpolation_Geometry/codegen/mex/evaluate_BSp/html/rtwtypes_h.html]

RTW Report - rtwtypes.h


|  |
| --- |
| File: rtwtypes.h  ```     1   /*     2    * Academic License - for use in teaching, academic research, and meeting     3    * course requirements at degree granting institutions only.  Not for     4    * government, commercial, or other organizational use.     5    *     6    * rtwtypes.h     7    *     8    * Code generation for function 'evaluate_BSp'     9    *    10    */    11       12   #ifndef RTWTYPES_H    13   #define RTWTYPES_H    14   #include "tmwtypes.h"    15   /*     16    * TRUE/FALSE definitions    17    */    18   #ifndef TRUE    19   #define TRUE (1U)    20   #endif     21   #ifndef FALSE    22   #define FALSE (0U)    23   #endif     24   #endif    25   /* End of code generation (rtwtypes.h) */    26 ``` |
