# Supplementary material for: Dynamic mechanochemical feedback between curved membranes and BAR protein self-organization
Source: Nat Commun. 2021 Nov 12;12:6550. doi: 10.1038/s41467-021-26591-3 (PMC8589976; doi:10.1038/s41467-021-26591-3)
Supplement: Supplementary file 26 — Reporting Summary [file 41467_2021_26591_MOESM26_ESM.pdf]

nature portfolio

Corresponding author(s): DBPR-NCOMMS-20-43949A

Last updated by author(s): Sep 17, 2021

Reporting Summary

Nature Portfolio wishes to improve the reproducibility of the work that we publish. This form provides structure for consistency and transparency in reporting. For further information on Nature Portfolio policies, see our Editorial Policies and the Editorial Policy Checklist.

Statistics

For all statistical analyses, confirm that the following items are present in the figure legend, table legend, main text, or Methods section.

n/a	Confirmed	
☐	☒	The exact sample size (*n*) for each experimental group/condition, given as a discrete number and unit of measurement
☐	☒	A statement on whether measurements were taken from distinct samples or whether the same sample was measured repeatedly
☐	☒	The statistical test(s) used AND whether they are one- or two-sided<br>*Only common tests should be described solely by name; describe more complex techniques in the Methods section.*
☒	☐	A description of all covariates tested
☐	☒	A description of any assumptions or corrections, such as tests of normality and adjustment for multiple comparisons
☐	☒	A full description of the statistical parameters including central tendency (e.g. means) or other basic estimates (e.g. regression coefficient) AND variation (e.g. standard deviation) or associated estimates of uncertainty (e.g. confidence intervals)
☒	☐	For null hypothesis testing, the test statistic (e.g. *F*, *t*, *r*) with confidence intervals, effect sizes, degrees of freedom and *P* value noted<br>*Give P values as exact values whenever suitable.*
☒	☐	For Bayesian analysis, information on the choice of priors and Markov chain Monte Carlo settings
☒	☐	For hierarchical and complex designs, identification of the appropriate level for tests and full reporting of outcomes
☒	☐	Estimates of effect sizes (e.g. Cohen's *d*, Pearson's *r*), indicating how they were calculated

Our web collection on statistics for biologists contains articles on many of the points above.

Software and code

Policy information about availability of computer code

Data collection	Lipid bilayer images were recorded with the Metamorph 7.7.11.0 or Micromanager-1.4 softwares. Live cell images were recorded with the Metamorph 7.7.11.0 software. Electron microscopy images were recorded with the analySIS 3.2 software. Labview 17.0 was used to control a home-made vacuum controller box. Monte Carlo simulations were generated using Matlab 2019b. Dynamical simulations were generated using Matlab2015a and 2016b.
Data analysis	Lipid bilayer images were analysed with ImageJ or Cell Profiler. Live cell and electron microscopy images were analysed with ImageJ. Data statistics tests were performed with Graphpad Prism 8.

For manuscripts utilizing custom algorithms or software that are central to the research but not yet described in published literature, software must be made available to editors and reviewers. We strongly encourage code deposition in a community repository (e.g. GitHub). See the Nature Portfolio guidelines for submitting code & software for further information.

Data

Policy information about availability of data

All manuscripts must include a data availability statement. This statement should provide the following information, where applicable:

- Accession codes, unique identifiers, or web links for publicly available datasets
- A description of any restrictions on data availability
- For clinical datasets or third party data, please ensure that the statement adheres to our policy

The authors declare that all quantifications supporting the findings of this study are available within the paper and its Supplementary information files, and are provided as a Source data file. Other data, such as raw and processed microscopy images, as well as all any other data generated in this study, are available from the corresponding authors on request.

Field-specific reporting

Please select the one below that is the best fit for your research. If you are not sure, read the appropriate sections before making your selection.

- [x] Life sciences
- [ ] Behavioural & social sciences
- [ ] Ecological, evolutionary & environmental sciences

For a reference copy of the document with all sections, see nature.com/documents/nr-reporting-summary-flat.pdf

Life sciences study design

All studies must disclose on these points even when the disclosure is negative.

Sample size	No statistical methods were used to determine sample size before execution of the experiments. Sample sizes for number of cells were determined based on previous experience by our group and others on similar experiments.
Data exclusions	No data was excluded from the analyses.
Replication	Cell experiments were replicated 3 times, no replication was excluded from the statistical analysis (all attempts at replication were successful).
Randomization	Cells were measured at random within each condition. 2 transfected cell per sample were chosen (the only criteria was sufficient fluorescence intensity) and stretched; 2 to 3 samples per day were measured in 3 different days.
Blinding	Blinding was not considered to be necessary due to the clear effects observed.

Reporting for specific materials, systems and methods

We require information from authors about some types of materials, experimental systems and methods used in many studies. Here, indicate whether each material, system or method listed is relevant to your study. If you are not sure if a list item applies to your research, read the appropriate section before selecting a response.

Materials & experimental systems

n/a	Involved in the study	
x		Antibodies
	x	Eukaryotic cell lines
x		Palaeontology and archaeology
x		Animals and other organisms
x		Human research participants
x		Clinical data
x		Dual use research of concern

Methods

n/a	Involved in the study	
x		ChIP-seq
x		Flow cytometry
x		MRI-based neuroimaging

Eukaryotic cell lines

Policy information about cell lines

Cell line source(s)	Normal Human Dermal Fibroblasts
Authentication	NHDF-Ad, Lonza, CC-2511
Mycoplasma contamination	Cell cultures were routinely checked for mycoplasma. Cell lines tested negative for micoplasma contamination.
Commonly misidentified lines (See ICLAC register)	No commonly misidentified lines were used.
