# Supplementary material for: Dynamic mechanochemical feedback between curved membranes and BAR protein self-organization
Source: Nat Commun. 2021 Nov 12;12:6550. doi: 10.1038/s41467-021-26591-3 (PMC8589976; doi:10.1038/s41467-021-26591-3)
Supplement: Supplementary file 25 — Supplementary software 1 [file 41467_2021_26591_MOESM25_ESM.zip › Supplementary Software 1/readme_code.pdf]

Supplementary Software 1

Contents

1 Code organization **2**

2 Instructions for use **2**

2.1 ”MainScript.m” 2

2.2 ”PPMainScript.m” 3

1 Code organization

The code computes the chemo-mechanical response of lipid membrane exposed to N-BAR proteins.
To reproduce the code download and uncompress the ”Code” folder, the typical install time in a ”normal” desktop computer happens in few minutes.

The code is organized as follows:

- ”MainScript.m” is the main script used to simulate the dynamics of the system
- ”PPMainScript.m” is the script to post-process the output files
- ”input” folder: input files used in ”MainScript.m” with different preexisting membrane templates
- ”Functionals” folder: custom functions
- ”Interpolation Geometry” folder: auxiliary calculations and structure for B-spline bases
- ”Matrices” folder: Residual and Jacobian matrices for the Newton Raphson method used to solve the binding-diffusion of proteins
- ”Reparametrization” folder: custom functions used to reparametrize the geometry
- ”output” folder: output of ”MainScript.m”

2 Instructions for use

2.1 ”MainScript.m”

The ”MainScript.m” run with MATLAB2016b and it allows to compute the chemo-mechanical interaction between a lipid membrane and N-BAR proteins. The expected output is the dynamical reshaping of lipid membranes due to proteins binding and diffusion. The expected run time on a ”normal” desktop computer is several hours.

The logical steps for the whole process are listed below:

1. Load input data (pregenerated membrane templates, saved in the ”input” folder)
2. Definition of state variables, initial conditions, boundary conditions and parameters
3. Definition of solver options and B-spline order
4. Loop to solve the problem
   - 4.1. Newton-Raphson method to solve proteins binding and diffusion
   - 4.2. ”fminunc” used to solve protein order
   - 4.3. ”fminunc” used to solve protein orientation
   - 4.4. ”fmincon” used to solve membrane shape
   - 4.5. adaptive time step
   - 4.6. reparametrization depending on the speed of the curve
   - 4.7. Visualization of state variables after the iteration
5. Save output file

2.2 ”PPMainScript.m”

The ”PPMainScript.m” run with MATLAB2015a and it allows to post-process the output files. The logical steps for the whole process are listed below:

1. Load output data (saved in the ”output” folder)
2. Print the evolution of the system
3. Print protein coverage
4. Print protein order
5. Print membrane shape
